# Climate-driven upward spread of forest fires in European mountain regions

**DOI:** 10.1038/s41467-026-72551-0

**Published:** 2026-04-30

**Authors:** Mirela Beloiu, Tomoki Loeillot, Verena C. Griess, Dimitris Poursanidis, Fanny Petibon

**Affiliations:** 1 Department of Environmental System Sciences, Institute of Terrestrial Ecosystems, ETH Zurich, Zurich, Switzerland; 2 Foundation for Research and Technology Hellas, Institute of Applied and Computational Mathematics, Vassilika Vouton, Heraklion, Greece

**Keywords:** Fire ecology, Climate-change ecology, Climate-change impacts, Forest ecology

## Abstract

Forest fires are an emerging threat to mountain regions yet changes in their elevational distribution and underlying drivers remain largely unknown. Here, we integrate satellite-derived burned area data, with climate, forest cover, and human footprint data into statistical models to assess fire activity and its drivers across eight European mountain regions. The analysis focuses on fires ≥800 m of elevation and burned area ≥30 ha for the period 2000-2025. We find that forest fires have spread upwards (~72 m per decade) and become more frequent after 2015, with fire occurrence increasing by 30% per year below 1400 m and 12% above that elevation. Changes in burned area are primarily driven by climate, including vapor pressure deficit, soil moisture, and precipitation. Fires above 1400 m are linked to extreme atmospheric aridity, indicating that climate change is enabling fire in ecosystems that have been historically buffered from burning. Further warming will increase fire susceptibility in European mountain regions, highlighting the need to protect mountain ecosystem functions and services through effective fire management.

## Abstract (web summary)

This study shows that forest fires are increasingly reaching higher elevations in European mountains, driven by drier climatic conditions and accelerating rapidly since 2015, thereby affecting ecosystems that were historically less exposed to fire.

## Introduction

Fire is a natural forest disturbance that shapes ecosystem dynamics^1^, regulates biogeochemical cycles^2^, and drives species selection^3^. Yet, wildfires have intensified worldwide, exacerbated by anthropogenic climate^4^ and land-use change^5^. Between 2017 and 2021, more than 27 million hectares of forest burned globally, including the Amazon rainforest^6^, the boreal forests of Siberia^7^, and the fire-prone areas of British Columbia^8^, California^9^, and Australia^10^. These events reflect widespread shifts in fire regimes, with longer fire seasons^11^, expanding burned area^12^, and more frequent extreme wildfire events marked by elevated fire intensity and severe impacts^10,13^. The consequences of such fires include reduced aboveground carbon sequestration^14^, biodiversity loss^15^, raw material shortages^16^, and threats to infrastructure and human health^17^. Europe has not been spared, for instance, improved fire management, including suppression and prevention policies, reduced burned areas in the late 20th century^18,19^, although prolonged fire exclusion may have increased fuel loads and connectivity, potentially limiting long-term effectiveness^20^. However, recent record-breaking fire seasons have reversed this trend, such as in 2017 (1.2 million ha, of which ~500,000 ha burned in Portugal and >120 lives were lost^21^), 2021 (500,566 ha), and 2022 (837,212 ha)^22,23^. While fires continue to be most frequent in the Mediterranean region, projections suggest that central and northern Europe will be affected due to high fuel loads that become more available under warming and drought conditions^24–26^. Although elevational increases in fire activity have been reported in some regions, it remains unclear whether current fire activity already signals a sustained upslope expansion into less-adapted European mountain systems, where even modest increases in fire activity could trigger disproportionate ecological and societal impacts.

Mountain forests are of particular concern, as they have long been buffered from fire by cool temperatures, persistent snow cover, lower human activity, and high moisture content^27^. Yet, these ecosystems are warming faster than the global average^28^, increasing the likelihood of fire at higher elevations^29,30^. High-elevation fires can destabilize soils, reduce water resources, and alter biodiversity^31,32^. In addition, the increasing frequency of fires at high elevations can have cascading effects on forest function and carbon storage. Fire activity is generally shaped by the interplay of ignitions, fuel, topography, and climate^33^. Most ignitions in Europe are human-caused, intensified by greater access and wildland–urban expansion^34,35^, but large fires require not only ignition but also conducive climatic conditions, including drought, heat, and wind^35^. However, large-scale analyses of how fires are advancing into Europe’s mountain forests remain missing. This gap is especially concerning, as most studies have concentrated on southern Europe and the French Alps^24,36^, while the vulnerability of temperate and high-elevation forests that are less adapted to fire remains largely unresolved.

Here, we assess whether forest fire occurrence has shifted to higher elevations in eight European mountain regions between 2000 and 2025. In addition, we identify the main environmental and anthropogenic drivers of fire activity across these landscapes. To address these questions, we combine remote sensing records of large fires (>30 ha) with climatic time series, fuel type, and human footprint data analyzed using statistical models. This approach allows us to test whether recent fire activity reflects a systematic upslope expansion and to disentangle the relative contributions of climate, vegetation, and human influence in enabling fire to spread into mountain ecosystems historically buffered from burning.

## Results

### Spatial, temporal, and elevational patterns of fire activity

**Fig. 1:**
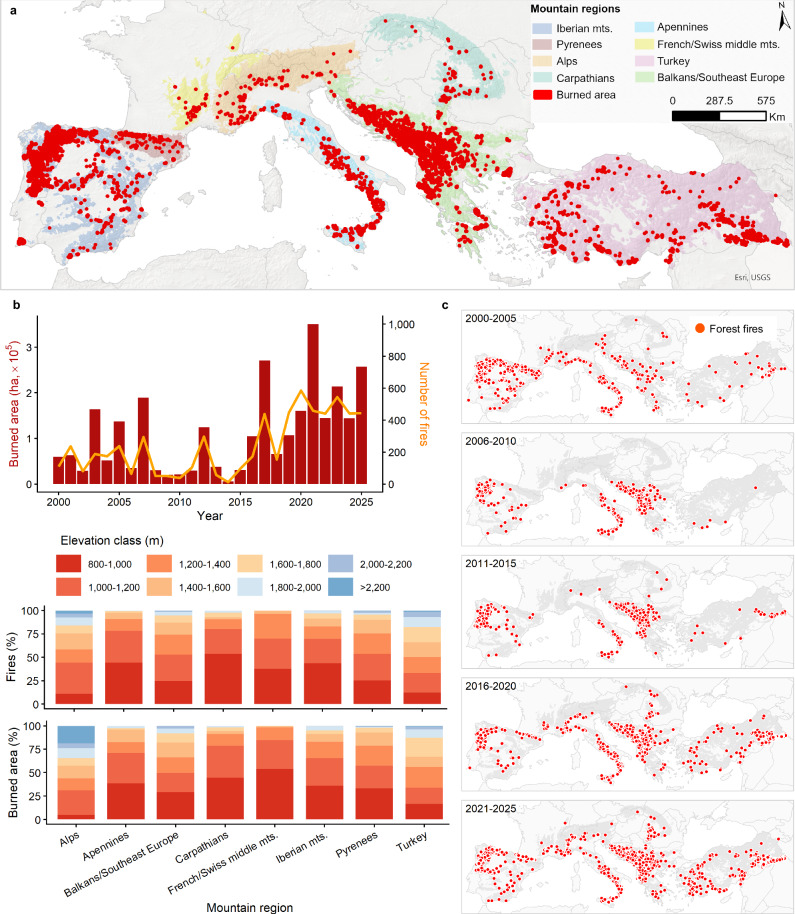
Spatial distribution and temporal evolution of forest fires across European mountain regions. **a** Spatial distribution of forest fires (no. = 6225) that occurred above 800 m elevation within eight European mountain regions from 2000 to 2025. **b** Evolution of burned area and number of fires within mountain regions of Europe and percentage of fire occurrences and burned area (ha) for each mountain region and elevation class. **c** Spatial distribution of forest fires across European mountain regions in five time periods (2000–2005, 2006–2010, 2011–2015, 2016–2020, and 2021–2025), illustrating the expansion and clustering of fire occurrences over time. Annual fire distributions are shown in Fig. S1. The extent of mountain regions was defined following Kapos et al.^59^, the country borders and the digital elevation model^71^ were used to enhance the visualization.

From 2000 to 2025, 6225 fires burned nearly 2.85 million hectares across the eight European mountain regions. Southern mountain ranges, including the Balkans, Iberian Mountains, and Turkey, dominated fire activity, accounting for 83% of all fire occurrences, with the highest activity in the Balkans (2773 fires; 45%), Iberian Mountains (1477; 24%), and Turkey (887; 14%) (Fig. 1a, Table S1). Fire activity showed marked peaks in 2005, 2007, 2012, 2017, 2020, and 2023, with the latter alone recording 545 fires (Fig. 1b, Table S2). Burned area followed a similar trajectory, with several record-breaking years in the past decade, underscoring the post-2015 surge. Most fires (75%) occurred between 800 and 1400 m, but 25% burned above 1400 m, in line with burned area trajectories (Table S3). Fires were concentrated at low elevations in the French/Swiss middle mountains (96%), Apennines (91%), Carpathians (91%), Iberian mountains (83%), as well as in the Balkans/Southeast Europe (74%) and the Pyrenees (76%) (Table S4). In contrast, high-elevation fires (>1400 m) accounted for nearly half of all events in Turkey (50%) and represented a substantial share in the Alps (42%) (Fig. 1b, Tables S3–6). A spatial clustering of fires across European mountain regions after 2015 is visible in Fig. 1c, with annual fire distributions shown for each year in Fig. S1.

### Upslope shift and increase in fire occurrence since 2015

**Fig. 2:**
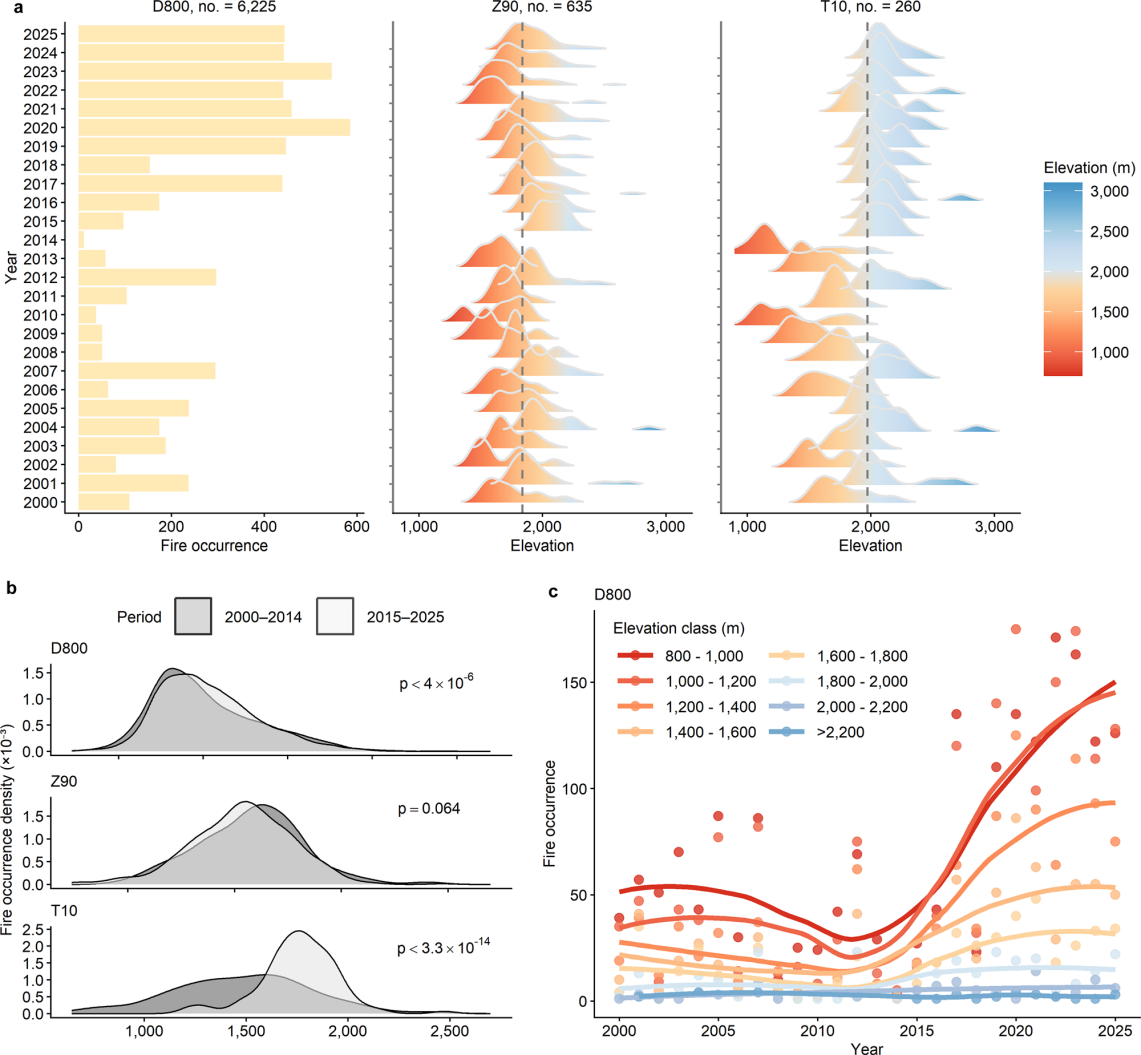
Elevation and temporal patterns of fire activity in European mountain regions from 2000 to 2025. **a** Annual fire occurrences above 800 m (D800), and elevation distributions for the 90th percentile fire activity (Z90) and the top ten fire activity (T10), showing a progressive shift toward higher elevations. **b** Fire occurrence density by mean elevation for the D800, Z90, and T10 datasets, comparing the periods 2000–2014 and 2015–2025. Kolmogorov–Smirnov test *p*-value shows significant distributional changes, with post-2015 fires occurring at higher elevation, indicating an upward shift in fire occurrence. **c** Temporal trends in fire occurrence by elevation class, with the strongest increases at lower elevations (800–1000 m), and smaller but consistent increases at higher elevations (>2000 m). Curves represent smoothed trends modeled using a loess function.

Fire activity intensified sharply after 2015, with annual counts consistently exceeding earlier maximums (Fig. 2a). After 2015, fire occurrence increased by ~9.5 fires yr^−^¹ below 1400 m (~29% yr^−^¹) and ~1 fire yr^−^¹ above 1400 m (~12% yr^−^¹) relative to pre-2015 mean fire frequency (Table S10). At the same time, fire occurrence shifted upslope. Elevation density distributions based on mean elevation show a clear upward displacement after 2015 across the full fire footprint (Fig. 2b, Table S3). Comparisons of early (2000–2014) and late (2015–2025) periods based on mean fire elevation show increases in fire elevation across all datasets: the full D800 record (median rose from 910 m to 1004 m, 72.10 m decade^−^¹), the 90th percentile of annual fire elevations (Z90; 1353 m to 1460 m, 82.35 m decade^−^¹), and the ten highest-elevation fires per year (T10; 1451 m to 1777 m, 250.28 m decade^−^¹; Fig. S2, Table S7). Analyses based on maximum fire elevation show similar trends, although with slightly lower rates of change. Linear regressions over 2000–2025 show positive but non-significant full-period trends (Table S8), supporting a post-2015 regime shift rather than a gradual increase. Temporal trends confirm consistent increases across all elevation classes, with the steepest gains at lower elevations (800–1000 m) but progressive rises even above 2200 m (Fig. 2c). Together, these findings demonstrate both an intensification of fire frequency and a significant upward expansion of fire occurrence into Europe’s mountain forests.

Fire seasonality differed among mountain regions and shifted over time (Figs. S3–5; Tables S11–13). For example, in the Balkans/Southeast Europe and Carpathians, peak fire occurrence shifted from late summer (August) before 2015 to early spring (March–April) after 2015 (Figs. S3–5), corresponding to advances of up to five months. In contrast, no significant seasonal shift was detected in Turkey or the Pyrenees (Table S13). Across regions, elevation of fire occurrence increased significantly through the calendar year (*β* = 13.7 m per month, *t* = 8.98), although region-specific trends varied (Table S14).

### Contrasting fire activity among European mountain regions

**Fig. 3:**
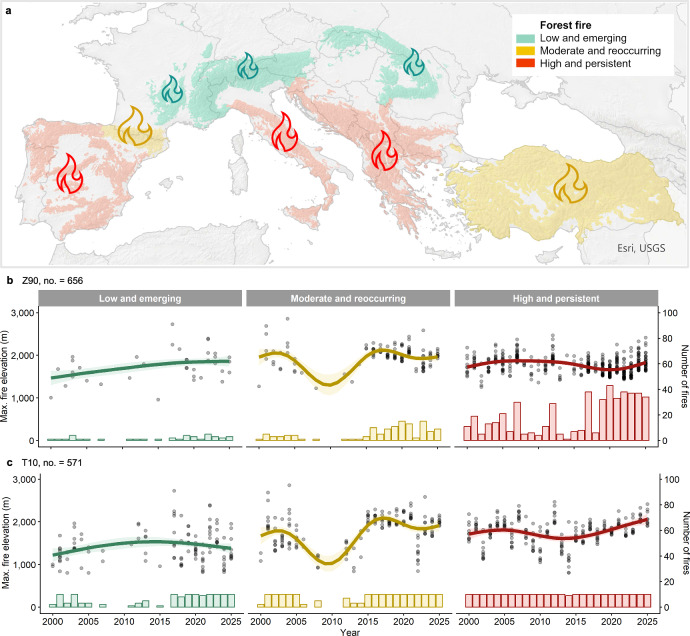
Spatiotemporal trends of forest fire activity across central and southern Europe from 2000 to 2025. **a** Classification of fire regimes into three types based on emergence and recurrence patterns: low and emerging, moderate and reoccurring, and high and persistent. **b**, **c** Trends in the maximum elevation of forest fires derived from the Z90 and T10 datasets, respectively, are shown separately for each fire regime type. Each point represents a fire occurrence. Solid lines show generalized additive models (GAMs) fits (*k* = 6) with shaded areas indicating 95% confidence intervals. Histograms below each panel represent annual fire counts per region. The extent of mountain regions was defined following Kapos et al.^59^, the country borders and the digital elevation model^71^ were used to enhance the visualization.

Based on fire emergence and recurrence patterns, we distinguished three distinct fire regime types across Europe’s mountains (Fig. 3). High and persistent fire regime type in the Balkans, Iberian Mountains and Apennines accounted for 78% of all events (4839 fires), while moderate and reoccurring fire regime type such as the Pyrenees and Turkey contributed 18% (1120 fires), and low and emerging fire regime type including the Alps, Carpathians and French/Swiss middle mountains only 4% (266 fires) (Table S2). High and persistent fire regime types sustained consistently high fire numbers (>50 per year in multiple years) and large events regularly reaching ~2000 m (Fig. 3b, c). In contrast, moderate and recurring fire regime types showed low activity before 2015 but a sharp post-2015 rise in both frequency and elevation, while low and emerging fire regime types only recorded consistent fire activity after 2017, at still modest numbers and with limited upslope expansion.

### Climatic constraints dominate fire activity above 1400 m

At the European level, significant differences in fuel type and climatic variables were detected above and below 1400 m (Table 1). Climatic variables consistently indicated that fires at higher elevations (>1400 m) occurred under drier and more stressful conditions than at lower elevations (<1400 m). Fires occurring below 1400 m were associated with higher water availability, reflected in greater actual evapotranspiration (AET), precipitation (PPT), soil moisture, and moderate fire weather danger (FWI). In contrast, fires above 1400 m were linked to drier and highly fire-conducive weather (FWI), characterized by elevated climatic water deficit (DEF), potential evapotranspiration (PET), vapor pressure deficit (VPD), and minimum and maximum annual temperature (Tmin and Tmax). Transitional vegetation was prevalent across elevations but dominated above 1400 m, accounting for 55% of burned area in high and persistent regions and 50% in moderate and reoccurring regions, compared to 41–42% below 1400 m. In contrast, broadleaf forests were generally more represented below 1400 m in high and persistent (17%) and low and emerging regions (43%), although patterns varied among regime types (Table 1, S15 and 16). Mixed and conifer forests remained comparatively less dominant overall, despite some elevation-specific contrasts (e.g., higher conifer and mixed shares above 1400 m in low and emerging regions). The human footprint was significantly lower above 1400 m.

**Table 1:** Elevation-based differences in median values of climatic, fuel, and human footprint variables across fire occurrences (D800) above and below 1400 m

	Variable	Median D800	Median <1400 m	Median >1400 m	*χ*^2^	*p*-Value	Sig.
Climatic	AET (mm)	38.38	**41.08**	28.50	1,828,455	4.30 × 10^−^^20^	***
	DEF (mm)	42.60	23.81	**93.21**	2,564,347	2.28 × 10^−^^14^	***
	PDSI	−2.81	−2.86	−2.66	2,269,547	0.37	ns
	PET (mm)	101.95	92.18	**135.18**	2,495,231	1.59 × 10^−^^09^	***
	PPT (mm)	30.83	**32.92**	21.92	1,923,724	2.45 × 10^−^^12^	***
	Soil moisture (mm)	40.91	**52.84**	29.57	1,774,573	2.23 × 10^−^^25^	***
	Tmin (°C)	10.07	8.49	**11.56**	2,324,994	0.03	*
	Tmax (°C)	21.37	18.85	**23.38**	2,419,116	1.72 × 10^−^^5^	***
	VPD (kPa)	0.83	0.69	**1.12**	2,550,769	2.82 × 10^−^^13^	***
	Wind speed (m/s)	2.59	**2.65**	2.42	1,904,039	8.95 × 10^−^^14^	***
	FWI	15.62	12.33	**23.02**	3,158,768	3.57 × 10^−^^28^	***
Fuel	Broadleaf (%)	17.26	**20.31**	9.09	2,300,317	5.84 × 10^−^^12^	***
	Conifer (%)	0	0	0	2,809,017	9.80 × 10^−^^8^	***
	Mixed (%)	0	0	0	2,789,469	3.39 × 10^−^^6^	***
	Transitional (%)	42.06	40.60	**47.03**	2,869,230	2.46 × 10^−^^6^	***
Human	Human footprint	11.25	11.56	**9.85**	2,010,794	1.90 × 10^−^^36^	***

*AET* actual evapotranspiration, *DEF* climatic water deficit, *PDSI* Palmer drought severity index, *PET* potential evapotranspiration, *PPT* precipitation, *Tmin* minimum annual temperature, *Tmax* maximum annual temperature, *VPD* vapor pressure deficit.

Fuel: broadleaf, coniferous, mixed forest cover, and transitional vegetation are given as percentage. In bold are marked prevalent environmental conditions below and above 1400 m.

Sig. indicates statistical significance based on *p*-values (*p < 0.05; **p < 0.01; ***p < 0.001; *ns* not significant).

### Climate drives burned area across European mountain regions

**Fig. 4:**
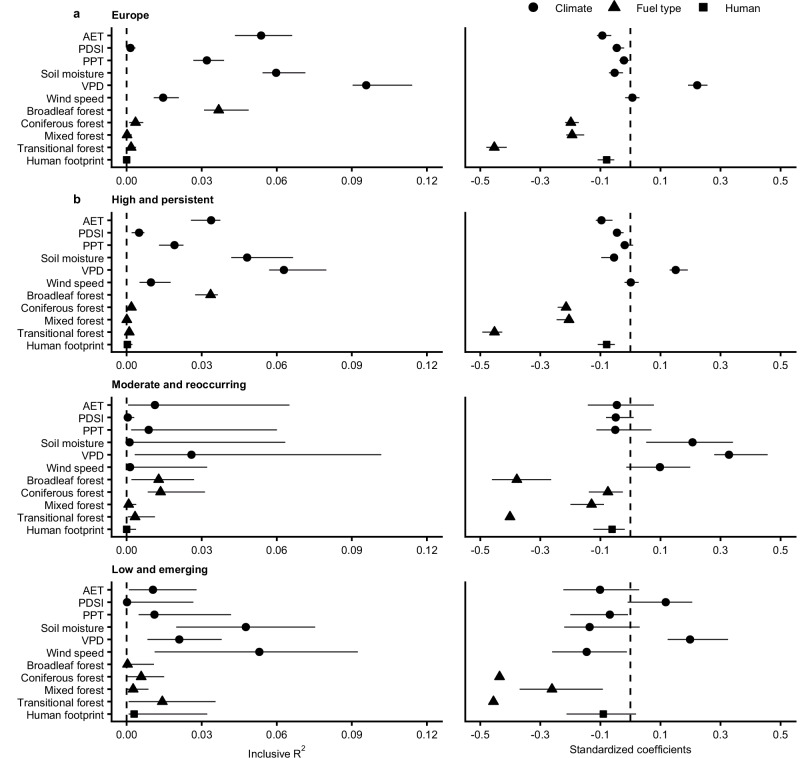
Predictors of burned area in European mountain regions and within fire regime types. **a** Europe-wide model (D800 dataset). **b** Models for individual fire regime types: high and persistent, moderate and reoccurring, low and emerging. Shown are the explained variance (inclusive *R*²) and standardized coefficients of climatic, fuel type, and human predictors of burned area. AET actual evapotranspiration, PDSI Palmer drought severity index, PPT precipitation, VPD vapor pressure deficit.

The largest fires occurred in regions with high and persistent or moderate and recurring fire regime types, compared with low and emerging fire regime types (Fig. S6a). Across Europe’s mountain regions, climatic variables explained most of the variance in burned area (*R*²*m* = 0.17; *R*²*c* = 0.21), far exceeding fuel type (4.2%) and human footprint (0.001%; Fig. 4 and Table S17). Vapor pressure deficit (VPD) was the strongest positive predictor (9.6%) across all regions and regimes, while precipitation and soil moisture had negative effects, underscoring the role of aridity in promoting large fires. Wind speed contributed additionally to the high and persistent fire regime type, whereas moderate and low fire regime types showed weaker associations. Vegetation cover also influenced fire size: broadleaf forests were linked to lower burned area, while transitional, mixed, and coniferous cover were linked to larger events (Fig. S6b, c). In high and persistent fire regime type, small fires were dominated by broadleaf vegetation, but the largest fires were associated with transitional and coniferous cover; moderate and reoccurring fire regime type showed similar though weaker patterns, while low and emerging regimes contained mixed vegetation across all fire sizes (Fig. S6b, c). Human footprint effects were detectable but consistently marginal at both continental and regional scales.

## Discussion

### Fires climb higher and intensify across Europe

From 2000 to 2025, fire occurrence and burned area increased across Europe’s mountain regions, with a marked surge after 2015. Earlier declines reported for southern Europe by Turco et al.^19^ align with our finding of relatively low activity until 2015, after which, all datasets (D800, Z90, T10) showed a consistent upslope shift, with peaks in 2019 and 2025 marking unprecedented fire activity (Fig. 2). These trends parallel global evidence of larger, more severe wildfires in the Amazon, Siberia, North America, and Australia^5,10,37,38^ and point to a reconfiguration of fire regimes in central and southern European mountains.

Elevational analyses revealed rising fire occurrence across all ranges (800 to >2000 m), with the steepest increase at mid-elevations (800–1400 m) and detectable gains above 2000 m. However, 1532 fires (24.6%) originate below 800 m but spread above this threshold, accounting for 1.49 million ha of burned area (52.4%), suggesting that climatic barriers that historically limited fire spread to higher elevations are weakening under increasingly dry and fire-prone conditions and highlighting implications for fire management strategies that are typically concentrated near human settlements rather than remote mountain areas. Further, we showed a shift toward spring fire activity in several mountain regions, consistent with increasing non-summer fires in Europe^39^. Although rare, high-elevation fires are particularly concerning as these forests are poorly adapted to fire. Unlike the western U.S., where the strongest trends occur above 3000 m^30^, Europe shows heterogeneous elevation-dependent warming^40,41^, yet the net effect is an upward spread of fires into ecosystems with little historical exposure.

Our classification into three fire regime types (Fig. 3) provides a framework to interpret how different patterns of fire activity relate to biophysical and socio-economic drivers. High and persistent fire regime types in Mediterranean mountains are consistent with evidence that intensifying summer heatwaves, prolonged drought, and declining precipitation are driving increasingly extreme fires in these areas^42^. By contrast, the sharp post-2015 increase in moderate and recurring fire regime types such as the Pyrenees and Turkey reflected the emergence of new high-elevation fire risks, aligning with recent extreme fire seasons^43^. Low and emerging fire regime types, including the Alps, French/Swiss middle mountains, and Carpathians, only began to experience recurrent fire activity after 2019, confirming projections that rapidly warming temperate and montane forests are becoming increasingly exposed to extreme fire weather^26,44^. This classification underscores the heterogeneity of mountain fire regime types in Europe, but also their convergence toward increasingly frequent and higher-elevation fires.

### Climate controls burned area across European mountain fire regimes

Climate stressors dominated fire activity across European mountain regions, with vegetation composition exerting a secondary role and human footprint only a marginal influence. Although most fires in Europe are human-ignited, our results showed that the spread and final size of large fires are primarily controlled by climatic conditions and fuel dryness. Our estimates of anthropogenic influence are likely conservative, as the Human footprint index is a coarse proxy and does not capture fine-scale ignition and accessibility gradients. Vapor pressure deficit (VPD) was the strongest predictor of burned area at both continental and regional scales, underscoring the role of atmospheric dryness in accelerating vegetation desiccation, increasing fuel flammability, and promoting fire spread^4^. Rising burned area under declining precipitation and soil moisture further highlights the central role of drought in shaping fire activity. Fires above 1,400 m occurred under much drier conditions than those below, with ~4× higher climatic water deficit and nearly double VPD, alongside lower precipitation and soil moisture (Table 1). This indicates that high-elevation fires are primarily climate-limited, while fires below 1,400 m are less constrained by aridity and more influenced by vegetation type and human activity. By contrast, low and emerging fire regime types were buffered by relatively low VPD and high soil moisture, a hydroclimatic protection likely to weaken as climate change intensifies. Similar upslope expansions linked to rising VPD have been reported in the western U.S.^30^, while globally, fire activity peaks during years of extreme fire weather and drought^45,46^. In Europe, the projected lengthening of the fire season by nearly a week^42^ signals growing risks even in forests historically buffered from burning.

Vegetation modulated fire behavior, for instance, broadleaf and transitional forests reduced fire size compared to conifers, yet coniferous fires, though rare, contributed disproportionately to the largest events, emphasizing the relevance of forest composition^47,48^. We represent fuels via harmonized land-cover fractions, which do not resolve fuel loads or vertical structure that may further influence mountain fire spread. Our results further demonstrate that vegetation effects interact with elevation and regional fire regime types, rather than acting independently of climatic gradients. The 1400 m threshold reflects not only a climatic boundary but also a structural transition in mountain forests, where tree height becomes constrained^49^. Warming is shifting the 1400 m threshold upward, increasing fuel cover at higher elevations; combined with rising dryness, this amplifies fire risk in previously buffered mountain forests.

### Implications and future directions

Our results demonstrate that climate-driven fires are expanding into European mountain forests that were historically buffered from burning. This upward shift (~72 m per decade), accompanied by increases in fire occurrence (~30% per year below 1400 m and ~12% above), has major implications for ecosystem functioning and global change feedbacks. Mountain forests are essential for carbon storage, biodiversity, and water resources^26^, yet even modest increases in high-elevation fire activity could destabilize soils, reduce water quality, and accelerate carbon losses^31,32^. High-elevation species, poorly adapted to fire, may face regeneration failures under rising aridity and water stress^50,51^, underscoring growing ecosystem vulnerability and the need for proactive management despite their remoteness and traditionally lower priority.

From a management perspective, these results highlight the need to anticipate fire risk in ecosystems not traditionally considered fire-prone. Most prevention and suppression strategies in Europe remain focused on the Mediterranean lowlands^52^, yet our results demonstrate increasing fire risk in Central European and montane regions. Proactive strategies, including fine-scale monitoring of fire weather and vegetation stress, cross-border coordination, adaptive silviculture and prescribed burning to reduce fuel continuity, load, and increase resistance to wildfires^53^, will be critical to maintain ecosystem services. Lessons from countries where early-warning systems and integrated fire management have reduced large-fire risk^19,54^ could inform similar strategies for mountain systems.

Future research should prioritize three key areas. First, higher-resolution fire data (e.g., Sentinel-2), harmonized fine-scale ignition metrics, and local incident reports should be integrated to better detect recent, small, and emerging fires at high elevations, as current inventories likely underestimate emerging fire activity in mountain systems. Between 1980 and 2010, the vast majority of Alpine wildfires were smaller than 10 ha^55^, indicating that shifts in fire activity at higher elevations may be underestimated in our analysis, which considers only fires >30 ha. Second, high-resolution climate products, such as easyclimate^56^, would further improve the detection and future predictions of fine-scale climate-fire interactions in complex mountain terrain. Further, socio-economic drivers such as land abandonment, tourism, and local ignition practices remain underrepresented in continental analyses^34,57^, yet they strongly shape ignition pressure. Third, the resilience and regeneration of fire-naïve mountain forests remain poorly understood, particularly under repeated burning and prolonged water stress^58^, and demand focused ecological research.

In summary, fire activity has increased and shifted upward across European mountains since 2015, driven by rising atmospheric aridity and declining moisture availability. As climate change weakens constraints on fire, these historically buffered, high-elevation ecosystems may transition into fire-prone systems. Without proactive adaptation, improved monitoring, cross-border management, and fire prevention, these mountain regions—long considered climate refugia—may transition into emerging fire hotspots, with cascading consequences for biodiversity, carbon storage, and water resources.

## Methods

### Fire data

The study area was divided into eight major mountain regions in southern and central Europe (Fig. 1a): Iberian mountains, Pyrenees, Apennines, French/Swiss middle mountains, Alps, Carpathians, Balkans/Southeast European mountains, and Turkish mountains. The boundaries of these mountain regions were delineated according to Kapos et al.^59^, based on elevation, slope, and local elevation range.

Fire data was obtained from the European Forest Fire Information System (EFFIS)^22^. The EFFIS dataset is based on the identification of abnormally hot regions from daily MODIS satellite imagery at 250 m ground spatial resolution and identifies fires with a size of ≥30 ha. In this study, all burned areas from 2000 to 2025 were used and clustered annually. We focused exclusively on forest fires by filtering events whose proportion of burned area corresponded to at least 50% of forest cover. The total percentage of forest cover within a given fire polygon was calculated by combining broad-leaved, conifer, mixed forest, and transitional woodland/shrub classes of the CORINE Land Cover provided directly in the EFFIS dataset. These areas encompass regions with various stages of tree growth (young, damaged, or dead), regenerating forests, and fully-grown trees covering less than 30% of the area, which are all recognized as forested areas^60^.

Forest fires were extracted for each mountain region above 800 m a.s.l. across central and southern Europe^59^. We derived the maximum elevation of each fire polygon from a digital elevation map (DEM) at 100 m resolution^61^ using the exact_extract function from the exactextractr package^62^. The threshold of 800 m was chosen to differentiate between low and high elevation fires, as it aligns with the elevation typically associated with sub-montane ecoregions^36^. We used maximum elevation to capture upward fire spread into mountain ecosystems, including fires originating below 800 m that extend into higher elevations (Table S2 and Fig. S8). Sensitivity analyses using mean elevation (≥800 m) produced consistent results. In addition, small mountain ranges, such as the Aegean Islands, Crete, Sardinia, and Corsica, were not included in the analysis. The final dataset after selection (i.e., forest fires above 800 m a.s.l., with ≥30 ha fire burn area, and between 2000 and 2025) comprised 6225 total fire polygons and is hereafter referred to as D800.

To analyze changes in the elevation of the highest forest fires, we generated two datasets using complementary metrics. The first (Z90) includes fires above the 90th percentile of the annual fire-elevation distribution^30^ (based on fires above 800 m; Fig. S7). The second (T10) includes the ten highest-elevation fires per year (T10). Both metrics were computed annually at two scales: (i) across the entire study area (continental scale Z90 = 635 and T10 = 260 fire occurrences) and (ii) separately for each mountain region (regional scale with Z90 = 656 and T10 = 571 fire occurrences). Together, these two datasets provide complementary perspectives on elevational shifts in fire activity, both at the continental scale and across regions with distinct fire activity. Hereafter, we refer to these datasets as the Z90 and T10 datasets, respectively.

To quantify elevational change, we used complementary approaches at the fire and yearly levels. Density distributions were based on pooled fire-level observations (2000–2014, 1998 fires; 2015–2025, 4227 fires) to visualize distributional shifts. Elevational change was quantified using yearly metrics (D800, Z90, T10), comparing period medians and calculating rates from period midpoints (Table S7). Annual metrics were used to ensure equal weighting of years and avoid bias from interannual variation in fire counts, and are shown as boxplots. Linear regressions of yearly metrics against Year (2000–2025) were fitted to assess continuous trends (Table S8).

### Fire activity

We assessed the fire activity as measured by fire occurrence and burned area within the eight major mountain regions. The eight European mountain regions were classified into three fire-regime categories based on fire emergence and recurrence patterns from 2000 to 2025. Low and emerging fire regime types (e.g., Alps, Carpathians, French/Swiss middle mountains) experienced consecutive years with fewer than 10 large fires (>30 ha), often with no fire before 2017. Moderate and recurring fire regime type (e.g., Pyrenees, Turkey) recorded fires between 2000 and 2005, followed by minimal to sporadic activity until 2015, and then a marked increase in frequency thereafter. High and persistent fire regime type (e.g., Iberian Mountains, Apennines, Balkans/Southeast Europe) consistently sustained high fire frequencies, often exceeding 50 fires per year, with recurrent extreme years throughout the study period (Table S2).

### Factors driving fire activity

Climate data was obtained from the TerraClimate database^63^. Monthly averages of actual evapotranspiration (AET), precipitation (PPT), soil moisture (SM), wind speed (WS), vapor pressure deficit (VPD), and Palmer drought severity index (PDSI) were downloaded for all years from 2000 to 2025 at a spatial resolution of 4 km. All climate layers were resampled to 1 km by bilinear interpolation and transformed to yearly averages to match the fire dataset’s time resolution. Temperature was additionally adjusted using a DEM-based lapse rate correction (−6.5 °C km^−^¹)^64^ to account for sub-grid elevational variability in mountainous terrain. Human footprint integrates multiple anthropogenic pressure variables, including built environments, population density, nighttime lights, cropland, pasture, roads, railways, and navigable waterways, into a composite index ranging from 0 (no detectable human pressure) to 50 (maximum cumulative human pressure), with values < 10 representing low human influence, 10–30 moderate disturbance (e.g., scattered agriculture), and >30 high-impact zones such as urbanized regions. Annual maps from 2000 to 2018 at 1 km spatial resolution were obtained from Mu et al.^65^. Detailed information on the data used in this study is summarized in Table 2 and some are plotted over time in Fig. S9. The Canadian fire weather index (FWI) (unitless, range 0 to >50, low to extreme) was evaluated but excluded from the driver analysis due to strong collinearity with several climatic predictors (e.g., VPD, DEF, PET) (Fig. S10). The mean, maximum, and minimum values of climatic and human predictors were extracted for each fire polygon, following the same approach as for elevation extraction.

**Table 2:** Summary of data used, their original spatial resolution, temporal coverage, and source

Data	Unit	Spatial resolution	Temporal coverage	Reference
Fire polygons	–	250 m	2000–2025 yearly	San-Miguel-Ayanz et al.^22^
Mountain regions	–	–	2008	Kapos et al.^59^
Copernicus digital elevation model (DEM) for Europe	m	100 m	2022	Neteler et al.^61^
Actual evapotranspiration (AET)	mm	4 km	2000–2024 monthly	Abatzoglou et al.^63^
Climatic water deficit (DEF)	mm	4 km	2000–2024 monthly	Abatzoglou et al.^63^
Potential evapotranspiration (PET)	mm	4 km	2000–2024 monthly	Abatzoglou et al.^63^
Temperature (T)	°C	4 km	2000–2024 monthly	Abatzoglou et al.^63^
Precipitation (PPT)	mm	4 km	2000–2024 monthly	Abatzoglou et al.^63^
Soil moisture (SM)	mm	4 km	2000–2024 monthly	Abatzoglou et al.^63^
Wind speed (WS)	m/s	4 km	2000–2024 monthly	Abatzoglou et al.^63^
Vapor pressure deficit (VPD)	kPa	4 km	2000–2024 monthly	Abatzoglou et al.^63^
Palmer drought severity index (PDSI)	unitless	4 km	2000–2024 monthly	Abatzoglou et al.^63^
Fire weather index (FWI)	unitless	~9 km	2000–2025 daily	CEMS^70^
Human footprint (HFP)	unitless	1 km	2000–2018 yearly	Mu et al.^65^

### Identification of elevation-dependent changes in burned area and fire number with time

To characterize regional fire activity across Europe, we first quantified trends in fire occurrence and burned area from 2000 to 2025. We assessed temporal changes in fire elevation using three complementary datasets: all fires above 800 m (D800), the 90th percentile of annual fire elevation (Z90), and the ten highest elevation fires per year per region (T10).

To test for differences in the distribution of fire elevations between the early (2000–2014) and late (2015–2025) study periods, we applied two-sample Kolmogorov–Smirnov tests. The latter period was chosen because it corresponds to a marked increase in fire occurrence and burned area in Europe. Temporal trends in fire occurrence across elevation classes (800–3000 m, in 200 m intervals, based on maximum elevation per polygon) were modeled using generalized linear mixed models (GLMMs) with year as a fixed effect and elevation class as a random effect. Model fit was evaluated using AIC, marginal *R*², and conditional *R*² values^66^. Models were fitted using the glmer function in the lme4 package^67^. Full model specifications and validation procedures are provided in Table S9. To visualize non-linear patterns (not for inference), we applied LOESS smoothers to fire occurrence per elevation class over time. Descriptive statistics of fire occurrence were additionally calculated for each elevation band before and after 2015 to highlight shifts in fire activity across the study period.

To assess trends in fire elevation across European mountain regions from 2000 to 2025, we analyzed maximum fire elevations using two derived datasets: the top ten highest-elevation fires per year per region (T10) and the 90th percentile of annual fire elevation (Z90). Temporal trends were modeled using generalized additive models (GAMs), with year as a predictor and group-specific smooths fitted for each fire regime type. The general model structure was:

$$E(Z\!\max,i)=\alpha+f{{\mathrm{Group}}}(ti)+\varepsilon i,\varepsilon i\sim N(0,\sigma 2)$$

Where $E(Z\!\max,i)$ is the expected maximum elevation of fire polygon $i$, $f{{\mathrm{Group}}}(ti)$ is a smooth function of year fitted separately for each fire regime type group, and ${{{\rm{\varepsilon }}}}{{{\rm{i}}}}$ is the residual error. An identity link function and a Gaussian error distribution were used. Models were fitted using the bam function in the mgcv package^68^, with basis dimension $k=7$ for the smooth terms. Model fit was evaluated using Akaike’s Information Criterion (AIC), explained deviance, and adjusted *R*².

### Drivers affecting fire occurrence and burned area

To compare the environmental conditions of fire occurrence across elevations, we assessed whether distributions differed significantly between two elevation groups using the non-parametric Wilcoxon rank–sum test (stats package). Specifically, we compared the median values of climatic, fuel type, and human variables between fires occurring above and below 1400 m a.s.l. The 1400 m threshold reflects the central tendency of statistically derived elevation breakpoints across climatic, fuel type, and human variables (Table S18), which cluster around 1400–1600 m at the continental scale. Vegetation composition within burned areas was quantified as the percentage cover of broadleaf, conifer, mixed, and transitional vegetation intersecting each fire polygon. Burned area and median vegetation cover were summarized by elevation class (≤1400 m and >1400 m) and fire regime type (Table S15), and by mountain region and elevation class (Table S5b). Burned area distributions across regime types were examined using log-transformed burned area and kernel density estimates (Fig. S6).

To identify drivers of burned area, we used a linear mixed-effects model using lme4 and lmerTest. Fixed effects captured climatic variables (vapor pressure deficit, soil moisture, minimum temperature, precipitation, Palmer Drought Severity Index, and wind speed), fuel composition (broadleaf, coniferous, mixed, and transitional), and human footprint. Pearson’s correlations among predictors and between predictors and log-scaled burned area were calculated. Predictors displaying a correlation higher than 0.7 were considered highly correlated and not included in the model (Fig. S11). A random intercept accounted for heterogeneity among mountain regions. The linear mixed-effects model specification was as in Eq. (1):

$${\log ({{\mathrm{Burned}}}\,{{\mathrm{area}}})}_{{{\mathrm{ij}}}}={\beta }_{0}+{\beta }_{1}*{{{\mathrm{vpd}}}}_{{{\mathrm{ij}}}}+{\beta }_{2}*{{\mathrm{soil}}}\,{{{\mathrm{moisture}}}}_{{{\mathrm{ij}}}}+{\beta }_{3}*{{{{{\rm{t}}}}}_{\min }}_{{{\mathrm{ij}}}}+ {\beta }_{4}*{{{\mathrm{precipitation}}}}_{{{\mathrm{ij}}}}+{\beta }_{5}*{{{\mathrm{pdsi}}}}_{{{\mathrm{ij}}}}+{\beta }_{6}*{{\mathrm{wind}}}\,{{{\mathrm{speed}}}}_{{{\mathrm{ij}}}}+{\beta }_{7}*{{\mathrm{broad}}}\,{{{\mathrm{leaf}}}}_{{{\mathrm{ij}}}}+{\beta }_{8}*{{{\mathrm{coniferous}}}}_{{{\mathrm{ij}}}}+{\beta }_{8}*{{{\mathrm{mixed}}}}_{{{\mathrm{ij}}}}+{\beta }_{9}*{{{\mathrm{transitional}}}}_{{{\mathrm{ij}}}}+{u}_{0{{{\rm{j}}}}}+{\varepsilon }_{{{\mathrm{ij}}}}$$

Where ${\beta }_{0}$ is the global intercept, ${\beta }_{1}\ldots {\beta }_{{{{\rm{p}}}}}$, are the fixed slopes of the predictors, ${u}_{0{{{\rm{j}}}}}$ is the random intercept for each mountain region *j*, and ${\varepsilon }_{{{\mathrm{ij}}}}$ the residual error.

The burned area was log-transformed to stabilize variance and reduce the influence of the largest fire occurrences. All predictor variables were centered and scaled (mean = 0, SD = 1) before analysis to allow for direct comparison of effect sizes and improve model convergence. *P*-values for fixed effects were obtained via Satterthwaite approximations. The explained variance was partitioned using partR2 package^69^; inclusive R2 quantifies the unique and shared variance associated with each predictor, while structure coefficients show the contribution of each predictor to the overall model prediction. Models were fitted both at the continental scale (D800) and for each mountain region with distinct fire regime types.

## Supplementary information

Supplementary Information

Transparent Peer Review file

## Data Availability

The data generated in this study have been deposited in the Zenodo file [Forest fire dataset for European mountain regions (2000–2025)] and can be accessed via this link [10.5281/zenodo.17078774]. These data were derived from the following resources available in the public domain (Table 2).
