# Supplementary material for: Climate-driven upward spread of forest fires in European mountain regions
Source: Nat Commun. 2026 Apr 30;17:5912. doi: 10.1038/s41467-026-72551-0 (PMC13338238; doi:10.1038/s41467-026-72551-0)
Supplement: Supplementary file 1 — Supplementary Information [file 41467_2026_72551_MOESM1_ESM.pdf]

Supplementary materials

Climate-driven upward spread of forest fires in European mountain regions

Mirela Beloiu[1a]*, Tomoki Loeillot[1], Verena C. Griess[1,] Dimitris Poursanidis[2], Fanny Petibon[1a]*

[1] *Department of Environmental System Sciences, Institute of Terrestrial Ecosystems, ETH Zurich, Zurich, Switzerland*

[2] *Foundation for Research and Technology Hellas, Institute of Applied and Computational Mathematics, 100 N. Plastira Str., Vassilika Vouton, 70013, Heraklion, Greece*

[a]*Authors contributed equally.*

**Corresponding author:*

Mirela Beloiu: mirela.beloiu@usys.ethz.ch

Fanny Petibon: fanny.petibon@usys.ethz.ch

1. Spatial, temporal, and elevational distribution of fire activity across mountain regions

Table S1. Aggregate fire distribution by mountain region (2000–2025).

Mountain	Fire number	Fire (%)	Area (%)	Total area (ha)
Iberian mts.	1,477	23.73	37.37	1,064,619
Balkans/Southeast Europe	2,773	44.55	36.83	1,049,224
Turkey	887	14.25	18.46	525,987
Apennines	589	9.46	4.47	127,413
Pyrenees	233	3.74	1.24	35,464
Alps	118	1.90	0.73	20,764
Carpathians	95	1.53	0.63	18,083
French/Swiss middle mts.	53	0.85	0.25	7,039
**Total**	**6,225**	**100.00**	**100.00**	**2,848,593**

Table S2. Distribution of burned area and number of fires across European mountain regions from 2000 to 2025. The table reports annual burned area (in hectares), total number of fires, and their breakdown by fire regime classification and specific mountain regions. Fire regimes are categorized as: Low and emerging (Alps, Carpathians, French/Swiss middle mountains), Moderate and reoccurring (Pyrenees, Turkey), and High and persistent (Apennines, Iberian Mountains, Balkans/Southeast Europe). No. fires (Number of fires) includes all fires, whereas Fires (no 01.01) excludes those recorded on 1st of January.

Year	Burned area (ha)	No. fires	Fires (no 0.1.01)	Low and emerging	Moderate and reoccurring	High and persistent	Alps	Carpa-thians	French/ Swiss middle mts.	Pyrenees	Turkey	Balkans/ Southeast Europe	Iberian mts.	Apennines
**2000**	59802	110	86	2	2	106	0	1	1	2	0	31	41	34
**2001**	63151	237	168	10	45	182	4	1	5	13	32	14	126	42
**2002**	29057	81	39	3	29	49	0	2	1	9	20	2	44	3
**2003**	163460	188	41	31	27	130	14	2	15	9	18	72	51	7
**2004**	51645	174	120	3	33	138	2	0	1	27	6	1	131	6
**2005**	137217	238	163	3	32	203	2	1	0	10	22	11	189	3
**2006**	35214	64	0	0	2	62	0	0	0	0	2	1	56	5
**2007**	189190	295	1	2	0	293	2	0	0	0	0	176	30	87
**2008**	30234	51	0	0	5	46	0	0	0	0	5	34	2	10
**2009**	19910	51	0	0	0	51	0	0	0	0	0	2	45	4
**2010**	21401	38	0	0	0	38	0	0	0	0	0	8	27	3
**2011**	29611	104	0	1	0	103	1	0	0	0	0	55	39	9
**2012**	124804	297	0	3	7	287	0	3	0	3	4	211	42	34
**2013**	38003	58	0	5	3	50	2	3	0	0	3	12	37	1
**2014**	5816	12	0	0	3	9	0	0	0	0	3	2	7	0
**2015**	30928	97	1	1	30	66	1	0	0	0	30	32	32	2
**2016**	105017	174	0	0	96	78	0	0	0	2	94	38	34	6
**2017**	270489	439	0	18	46	375	15	2	1	2	44	205	46	124
**2018**	65798	154	0	8	89	57	3	5	0	3	86	37	16	4
**2019**	107398	447	0	33	102	312	16	13	4	17	85	272	29	11
**2020**	160343	585	1	21	143	421	6	11	4	7	136	369	20	32
**2021**	350127	459	0	9	123	327	6	1	2	6	117	236	28	63
**2022**	144641	441	2	50	11	380	23	22	5	4	7	295	54	31
**2023**	213933	545	1	27	149	369	13	0	14	111	38	53	295	21

Year	Burned area (ha)	No. fires	Fires (no 0.1.01)	Low and emerging	Moderate and reoccurring	High and persistent	Alps	Carpa-thians	French/ Swiss middle mts.	Pyrenees	Turkey	Balkans/ Southeast Europe	Iberian mts.	Apennines
**2024**	144049	442	0	11	61	370	2	9	0	5	56	325	14	31
**2025**	257355	444	0	25	82	337	6	19	0	3	79	279	42	16
**Total**	**2848593**	**6225**	**623**	**266**	**1120**	**4839**	**118**	**95**	**53**	**233**	**887**	**2773**	**1477**	**589**

Table S3. Aggregate fire distribution by maximum elevation class across all European mountain regions (2000–2025).

Elevation class (m)	Fire number	Fire (%)	Total area (ha)	Area (%)
800–1000	1,842	29.59	846,130	29.70
1000–1200	1,692	27.18	687,518	24.14
1200–1400	1,105	17.75	506,065	17.77
1400–1600	722	11.60	338,873	11.90
1600–1800	477	7.66	252,203	8.85
1800–2000	260	4.18	158,307	5.56
2000–2200	91	1.46	46,098	1.62
>2200	36	0.58	13,399	0.47

Table S4. Aggregate fire distribution across European mountain regions (2000–2025). Distribution is summarized by mountain range and elevation class (below and above 1,400 m), based on maximum fire elevation.

Mountain	Elevation class (m)	Fire number	Total area (ha)	Fire (%)	Area (%)
Alps	<=1,400 m	69	9,137	58.47	44
Alps	>1,400 m	49	11,627	41.53	56
Apennines	<=1,400 m	536	105,404	91	82.73
Apennines	>1,400 m	53	22,009	9	17.27
Balkans/Southeast Europe	<=1,400 m	2051	695,751	73.96	66.31
Balkans/Southeast Europe	>1,400 m	722	353,473	26.04	33.69
Carpathians	<=1,400 m	86	16,479	90.53	91.13
Carpathians	>1,400 m	9	1,604	9.47	8.87
French/Swiss middle mts.	<=1,400 m	51	6,916	96.23	98.25
French/Swiss middle mts.	>1,400 m	2	123	3.77	1.75
Iberian mts.	<=1,400 m	1,224	883,381	82.87	82.98
Iberian mts.	>1,400 m	253	181,238	17.13	17.02
Pyrenees	<=1,400 m	176	27,939	75.54	78.78
Pyrenees	>1,400 m	57	7,525	24.46	21.22
Turkey	<=1,400 m	446	294,706	50.28	56.03
Turkey	>1,400 m	441	231,281	49.72	43.97

Table S5. Distribution of fire events by mountain region and elevation class (2000–2025).

No.	Mountain	Elevation class (m)	Fire number	Total area (ha)	Fire (%)	Area (%)
1	Alps	800-1000	13	1005	11.02	4.84
2	Alps	1000-1200	39	5483	33.05	26.41
3	Alps	1200-1400	17	2649	14.41	12.76
4	Alps	1400-1600	20	2790	16.95	13.44
5	Alps	1600-1800	10	1668	8.47	8.03
6	Alps	1800-2000	10	2196	8.47	10.58
7	Alps	2000-2200	5	1046	4.24	5.04
8	Alps	>2200	4	3927	3.39	18.91
9	Apennines	800-1000	260	49249	44.14	38.65
10	Apennines	1000-1200	201	41343	34.13	32.45
11	Apennines	1200-1400	75	14812	12.73	11.63
12	Apennines	1400-1600	38	17074	6.45	13.4
13	Apennines	1600-1800	10	1798	1.7	1.41
14	Apennines	1800-2000	5	3137	0.85	2.46
15	Balkans/Southeast Europe	800-1000	685	303390	24.7	28.92
16	Balkans/Southeast Europe	1000-1200	776	218279	27.98	20.8
17	Balkans/Southeast Europe	1200-1400	590	174082	21.28	16.59
18	Balkans/Southeast Europe	1400-1600	363	165899	13.09	15.81
19	Balkans/Southeast Europe	1600-1800	210	101501	7.57	9.67
20	Balkans/Southeast Europe	1800-2000	108	57103	3.89	5.44
21	Balkans/Southeast Europe	2000-2200	28	23362	1.01	2.23
22	Balkans/Southeast Europe	>2200	13	5608	0.47	0.53
23	Carpathians	800-1000	51	8053	53.68	44.53
24	Carpathians	1000-1200	25	6124	26.32	33.87
25	Carpathians	1200-1400	10	2302	10.53	12.73
26	Carpathians	1400-1600	2	589	2.11	3.26
27	Carpathians	1600-1800	5	739	5.26	4.09
28	Carpathians	1800-2000	2	276	2.11	1.53
29	French/Swiss middle mts.	800-1000	20	3804	37.74	54.04
30	French/Swiss middle mts.	1000-1200	17	2167	32.08	30.79
31	French/Swiss middle mts.	1200-1400	14	945	26.42	13.43
32	French/Swiss middle mts.	1400-1600	2	123	3.77	1.75
33	Iberian mts.	800-1000	645	380871	43.67	35.78
34	Iberian mts.	1000-1200	382	316093	25.86	29.69
35	Iberian mts.	1200-1400	197	186417	13.34	17.51
36	Iberian mts.	1400-1600	124	89406	8.4	8.4

No.	Mountain	Elevation class (m)	Fire number	Total area (ha)	Fire (%)	Area (%)
37	Iberian mts.	1600-1800	85	39432	5.75	3.7
38	Iberian mts.	1800-2000	35	46300	2.37	4.35
39	Iberian mts.	2000-2200	7	5860	0.47	0.55
40	Iberian mts.	>2200	2	240	0.14	0.02
41	Pyrenees	800-1000	59	11813	25.32	33.31
42	Pyrenees	1000-1200	66	8489	28.33	23.94
43	Pyrenees	1200-1400	51	7637	21.89	21.53
44	Pyrenees	1400-1600	34	4961	14.59	13.99
45	Pyrenees	1600-1800	12	1813	5.15	5.11
46	Pyrenees	1800-2000	5	288	2.15	0.81
47	Pyrenees	2000-2200	4	304	1.72	0.86
48	Pyrenees	>2200	2	159	0.86	0.45
49	Turkey	800-1000	109	87945	12.29	16.72
50	Turkey	1000-1200	186	89540	20.97	17.02
51	Turkey	1200-1400	151	117221	17.02	22.29
52	Turkey	1400-1600	139	58031	15.67	11.03
53	Turkey	1600-1800	145	105252	16.35	20.01
54	Turkey	1800-2000	95	49007	10.71	9.32
55	Turkey	2000-2200	47	15526	5.3	2.95
56	Turkey	>2200	15	3465	1.69	0.66

Table S6. Elevation characteristics of forest fire events across European mountain regions (2000–2025). The table reports the number of fires and summary statistics of fire elevation derived from the minimum (Z_min), mean (Z_mean), and maximum (Z_max) elevation of burned polygons.

Mountain	Fires (no.)	Mean Z_mean (m)	Median Z_mean (m)	Mean Z_max (m)	Median Z_max (m)	Mean Z_min (m)	Median Z_min (m)
Alps	118	1079	999	1382	1291	795	784
Apennines	589	859	829	1074	1031	638	643
Balkans/Southeast Europe	2773	1042	1004	1233	1175	845	835
Carpathians	95	871	812	1060	978	676	623
French/Swiss middle mts.	53	985	946	1095	1085	853	817
Iberian mts.	1477	955	895	1125	1045	781	758
Pyrenees	233	976	932	1225	1173	737	690
Turkey	887	1219	1201	1428	1399	1030	1029

A total of 1,532 fires (24.6%) had a mean elevation below 800 m but reached elevations above 800 m. These fires accounted for 1.49 million hectares of burned area (52.4% of total burned area), indicating that many large fires originate at lower elevations but spread upslope into mountain environments.

2. Annual forest fire occurrence across European mountain regions (2000 to 2025)

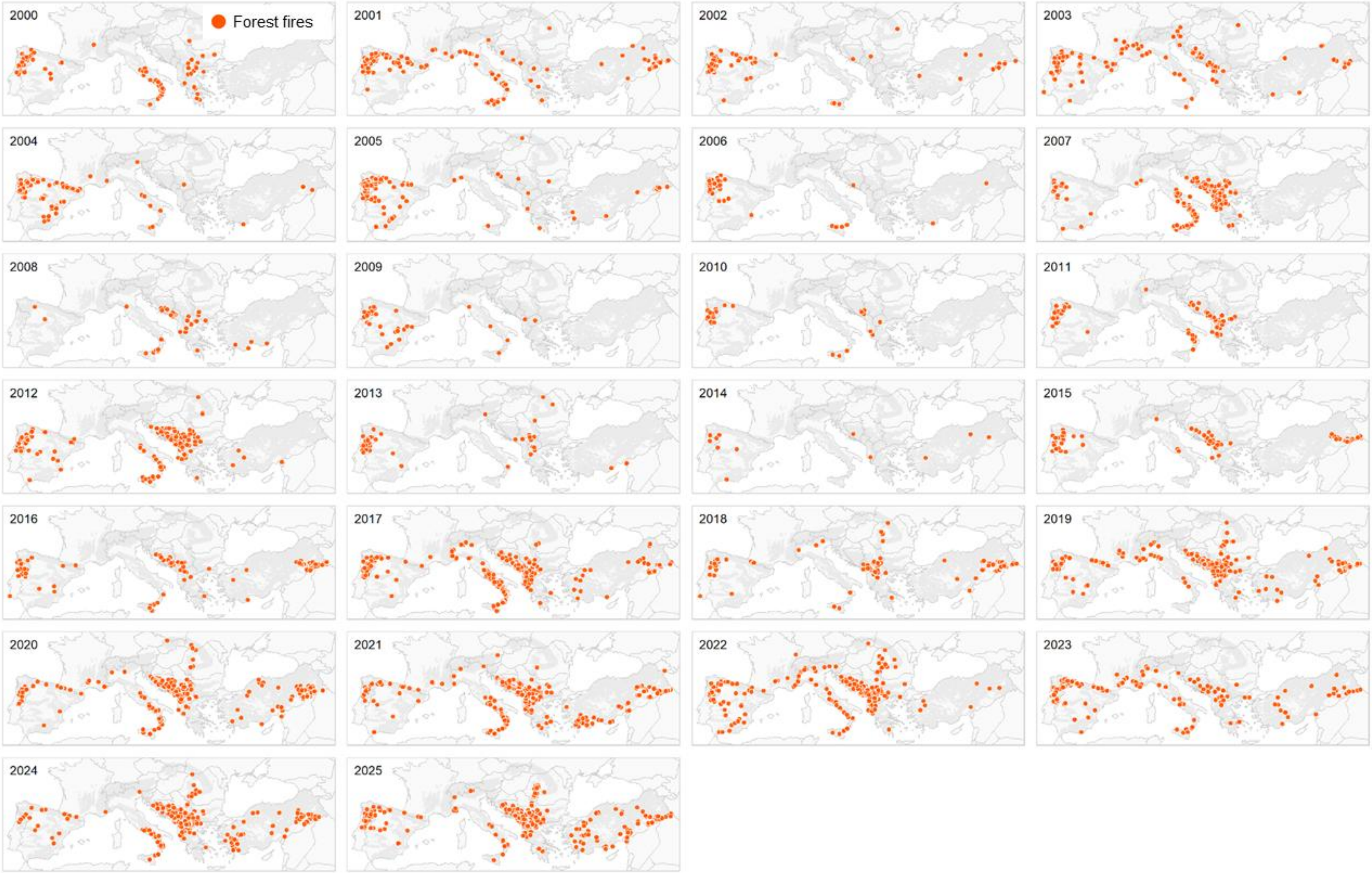

Fig. S1. Annual spatial distribution of forest fires in the European mountain study area (2000 to 2025). Each panel shows fire locations for a single year as point representations of burned area events overlaid on country outlines, enabling visual comparison of year-to-year variability and shifts in hotspots across the mountain regions.

3. Upslope shift in European mountain fire elevation before and after 2015

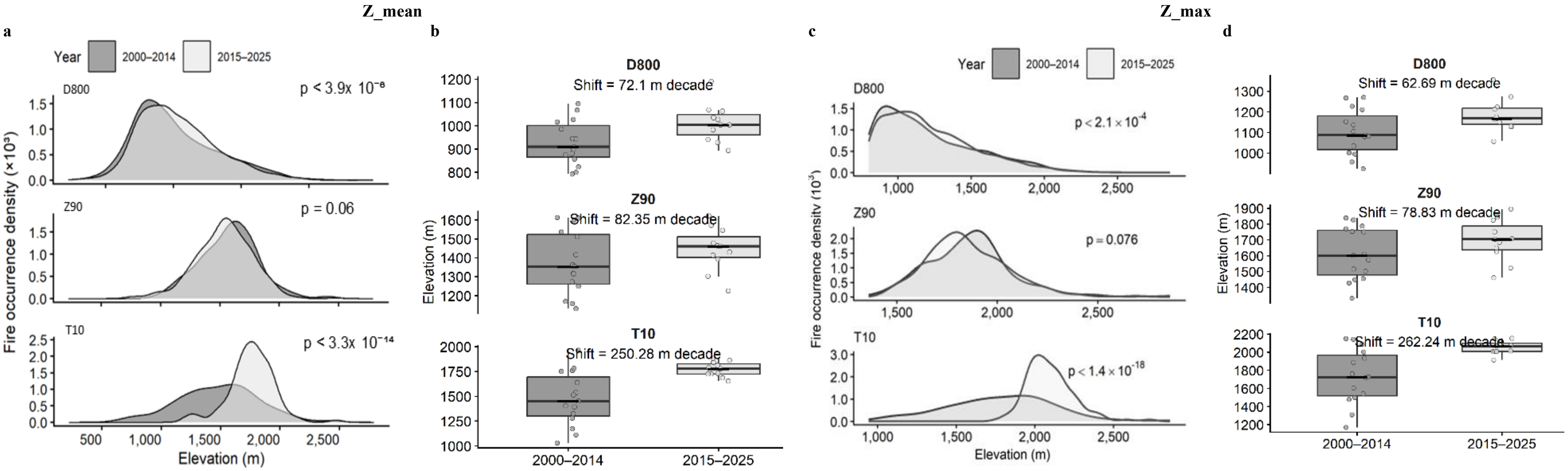

Figure S2. Upslope shift in European mountain fire elevation before and after 2015 based on mean and maximum fire elevation. a, b Density distributions and yearly boxplots of fire elevation metrics based on mean fire elevation (Z_mean). **c, d** Same as in **a, b** but based on maximum fire elevation (Z_max). Fire events were filtered to include only those reaching ≥ 800 m (Z_max ≥ 800 m). Three metrics are shown: D800, the median elevation of fires reaching ≥ 800 m; Z90, the yearly 90th percentile; and T10, the median elevation of the 10 highest-elevation fires per year. Boxplots show the distribution of yearly values (points), with boxes indicating the interquartile range, central lines the median, and whiskers the data range. Shifts (m decade^{-1}) are calculated from differences between period medians (2000–2014 vs. 2015–2025). P-values indicate differences between periods based on Kolmogorov–Smirnov tests.

Table S7. Upslope shift in European mountain fire elevation before and after 2015. Fires were filtered to Z_max ≥ 800 m. For each year, D800 (median), Z90 (90th percentile), and T10 (median of the 10 highest fires) were computed using either Z_max or Z_mean per polygon. Z_mean = mean fire elevation per fire polygon and Z_max = maximum fire elevation per fire polygon. Z_max is constrained by the upper elevational limits of fires, whereas Z_mean reflects where fires predominantly occur; thus, when the overall distribution shifts upslope, Z_mean can increase more than Z_max.

The table reports changes in yearly elevation metrics to quantify temporal shifts, giving equal weight to each year and avoiding bias from interannual variation in fire counts. Boxplots are based on the same yearly metrics and are therefore consistent with the table, whereas density plots use pooled fire-level observations and primarily reflect distributional differences, which can lead to contrasting patterns (Figure S2).

Data	Definition	Median 2000–2014 (m)	Median 2015–2025 (m)	Elevation change (m)	Shift (m yr^{-1})	Shift (m $decade^{-1}$)
D800	Median Z_mean of all fires ≥ 800 m per year	909.87	1,003.60	93.73	7.21	72.10
Z90	Yearly 90th percentile of Z_mean	1,352.54	1,459.59	107.05	8.24	82.35
T10	Median Z_mean of the 10 highest fires per year	1,451.16	1,776.52	325.37	25.03	250.28
D800	Median Z_max of all fires ≥ 800 m per year	1,086.77	1,168.26	81.49	6.27	62.69
Z90	Yearly 90th percentile of Z_max	1,600.87	1,703.35	102.48	7.88	78.83
T10	Median Z_max of the 10 highest fires per year	1,725.25	2,066.16	340.91	26.22	262.24

Table S8. Linear regression slopes of yearly elevational fire metrics (2000–2025). Slopes are expressed in meters per year and meters per decade. Confidence intervals include zero, indicating that the full-period linear trend is not statistically significant, supporting the interpretation of a post-2015 regime shift rather than a gradual monotonic increase. Z_mean = mean fire elevation per fire polygon and Z_max = maximum fire elevation per fire polygon.

	**Metric**	**Shift (m yr^{-1})**	**95% CI (m yr^{-1})**	**Shift (m decade^{-1})**	**R^2**	**p-value**
**Z_mean**	D800	3.38	−1.79 to 8.54	33.76	0.070	0.190
	Z90	2.52	−5.62 to 10.67	25.21	0.017	0.529
	T10	12.23	−0.89 to 25.36	122.32	0.134	0.066
**Z_max**	D800	3.30	−2.31 to 8.91	33.01	0.058	0.236
	Z90	2.65	−5.78 to 11.07	26.48	0.017	0.523
	T10	12.16	−2.47 to 26.80	121.65	0.109	0.099

Table S9. Summary of generalized linear mixed models of the temporal trends in fire occurrence with elevation classes as a random effect. Data were centered and scaled prior to analysis. AIC = Akaike's Information Criterion. Year was included as a continuous predictor to estimate the overall directional temporal trend in fire occurrence across the study period rather than interannual variation. Random slopes of year within elevation classes (Year | Elevation class) were included to allow the temporal trend to vary among elevation bands. Models assumed a Gaussian error distribution because fire counts per elevation class were approximately normally distributed after aggregation.

**Model**		**Fire occurrence**
**Formula**		Fire occurrence ~ Year + (Year|Elevation class)
**Estimate ± Standard error**	Intercept	28.76 ± 8.87
	Slope	18.72 ± 6.36 (equivalent to 2.44 fires per year)
P-value	Intercept	0.0012
	Slope	0.0033
**Number of observations**		185
**AIC**		**1,729.2**
**Marginal R-squared (R^2m)**		0.236
**Conditional R-squared (R^2c)**		0.642

Table S10. Temporal trends of forest fire occurrence before and after 2015. Annual fire counts were calculated for each elevation class. Before 2015 (2000–2014), values are reported as mean and standard deviation of yearly fire occurrence, as no significant temporal trends were detected (see Fig. 2c). After 2015 (2015–2025), linear regressions of yearly fire counts against Year were fitted for each elevation class; slopes (fires yr^{-1}) indicate the annual rate of change in fire occurrence and R^2 values the strength of the trend. For example, fire occurrence increased by 11.02 fires yr^{-1} at 800–1,000 m, compared to only 0.06 fires yr^{-1} above 2,200 m, indicating substantially stronger increases at lower elevations. Before 2015 there was no slope since it was not significant (see Fig. 2c in the manuscript).

Before 2015, mean annual fire occurrence averaged 32.8 fires yr^{-1} below 1,400 m and 8.2 fires yr^{-1} above, indicating 4 times higher fire occurrence (≈300% higher) at lower elevations. After 2015, fire occurrence increased across all elevation classes but much more strongly below 1,400 m (+9.5 fires yr^{-1}; ~29% yr^{-1}) than above (+1.0 fires yr^{-1}; ~12% yr^{-1}), representing an approximately 9-fold stronger increase at lower elevations.

	before 2015		after 2015	
**Elevation class (m)**	**Mean**	**SD**	**Slope**	**R^2**
**800 – 1’000**	44.33	25.61	11.02	0.484
**1’000 – 1’200**	33.60	26.55	10.94	0.429
**1’200 – 1’400**	20.47	17.18	6.67	0.426
**1’400 – 1’600**	17.00	13.68	3.06	0.215
**1’600 – 1’800**	10.93	10.79	1.58	0.223
**1’800 – 2’000**	7.07	7.98	0.34	0.029
**2’000 – 2’200**	2.67	2.29	0.05	0.002
**> 2’200**	3.50	1.00	0.06	0.037

4. Temporal changes in fire seasonality across European mountains

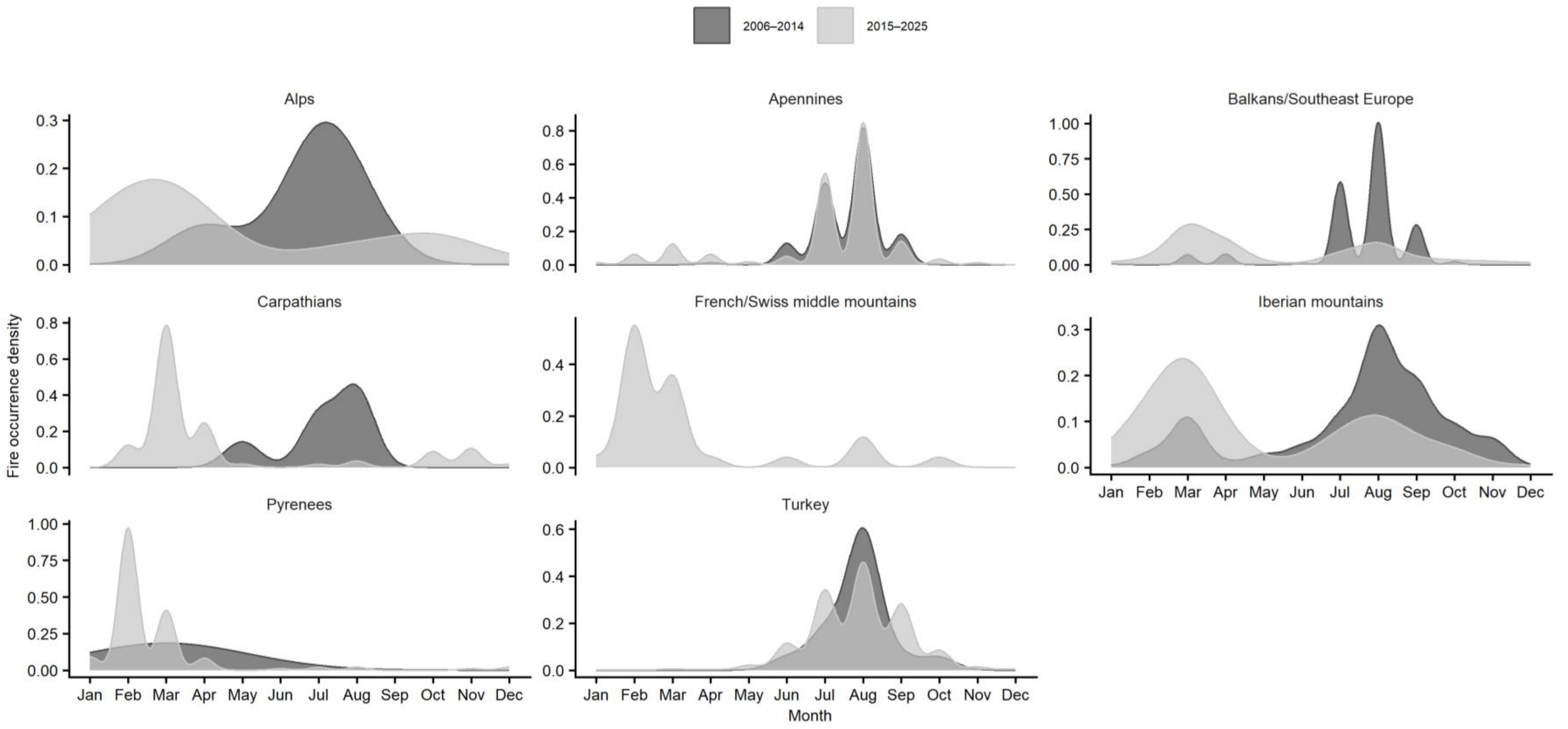

Fig. S3. Seasonal distribution of fire occurrence across European mountain regions (2006–2025 dataset). Kernel density distributions of monthly fire occurrence for each mountain region comparing two periods: 2006–2014 (dark grey) and 2015–2025 (light grey). Densities are scaled within each region (free y axis). The figure illustrates shifts in peak fire timing and changes in seasonal concentration between periods. Number of fires: 5,197 (2006-2025). The forest fires from 2000 to 2005 were removed since many fires do not have exact date, hence were recorded on 1st of January (see Tables S2).

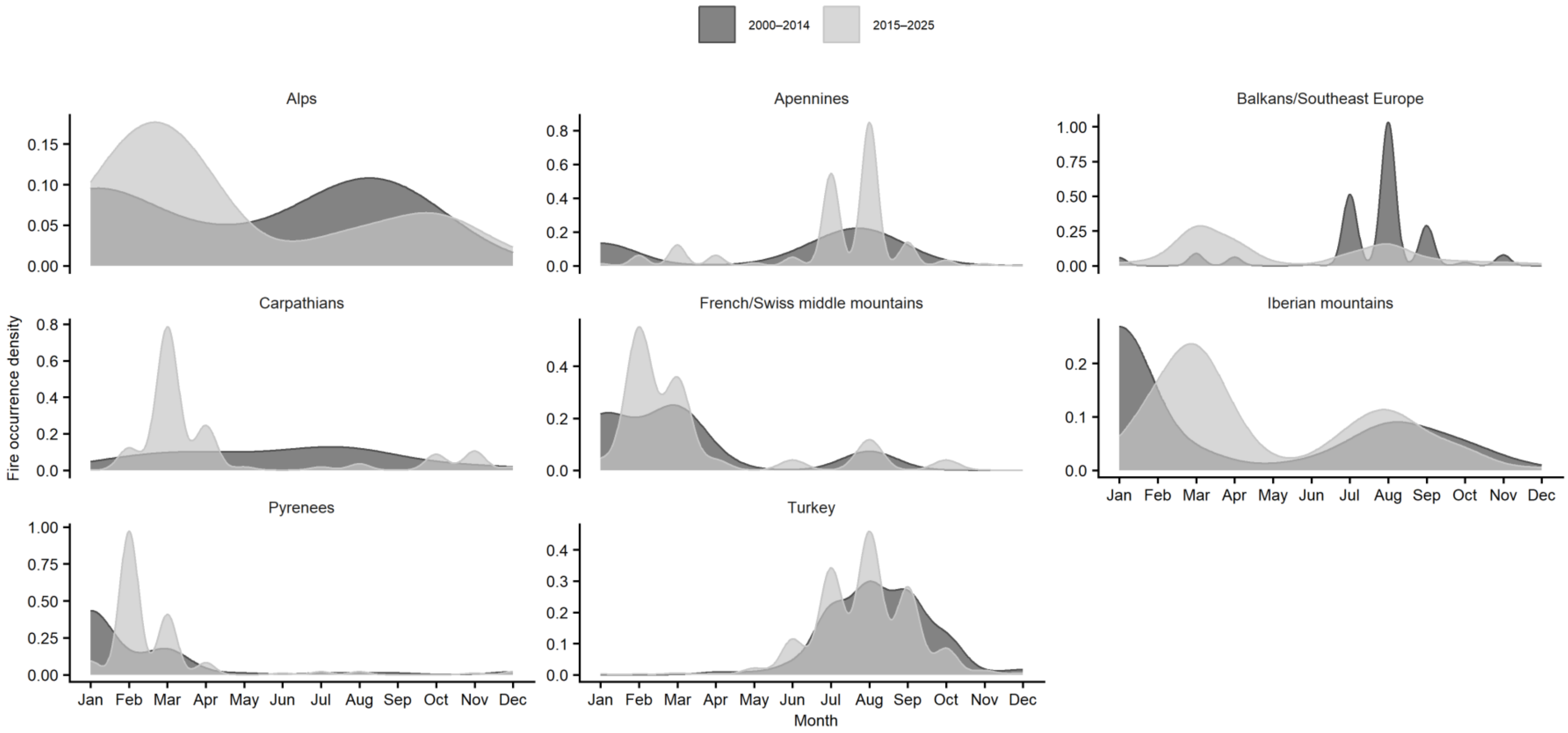

Fig. S4. Seasonal distribution of fire occurrence across European mountain regions (2000–2025 dataset). Kernel density distributions of monthly fire occurrence for each mountain region comparing two periods: 2000–2014 (dark grey) and 2015–2025 (light grey). Densities are scaled within each region (free y axis). The figure highlights long term changes in fire season timing and the relative concentration of fire occurrence over the past two decades. The figure includes all fires (no.=6225), also the one recorded on 1st January from 2000 to 2005 (see Table S2).

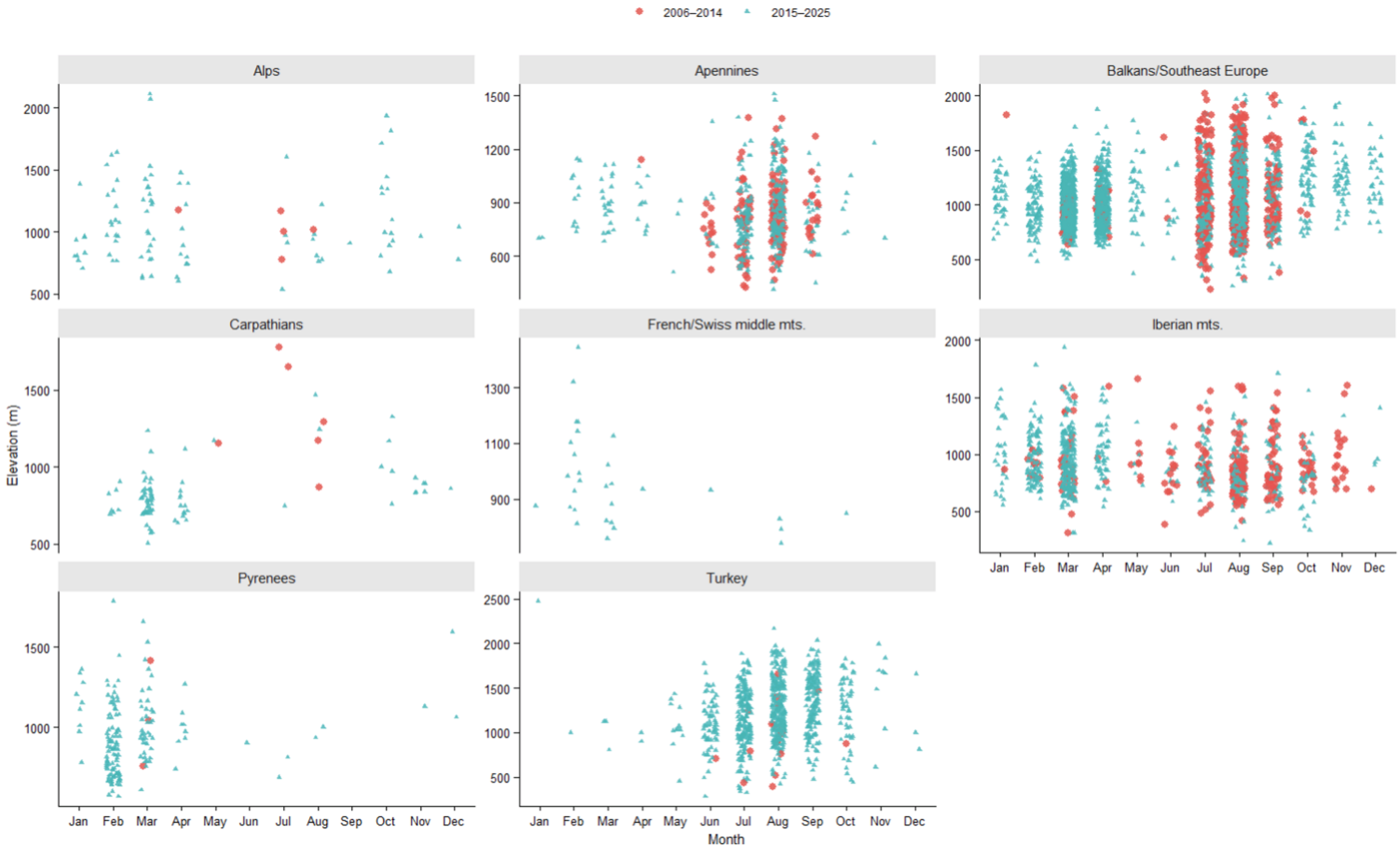

Fig. S5. Seasonal distribution of fire elevation across European mountain regions (2006–2025). Each point represents a single fire occurrence plotted by month and elevation. Red circles indicate fires during 2006–2014 and teal triangles indicate fires during 2015–2025. The figure illustrates seasonal shifts in the elevation range of fires, with winter fires generally occurring at lower elevations and summer fires extending to higher elevations in several mountain regions. Number of fires: 5,197 (2006-2025).

5. Seasonal fire occurrence by mountain region and period

Table S11. Seasonal distribution of fire occurrence by mountain region and period (2006–2014; 2015–2025). For each region, the table reports the number of fires, peak month (month with highest fire frequency), peak share (% of annual fires occurring in the peak month), and median fire month. The forest fires from 2000 to 2005 were removed since many fires do not have exact date, hence were recorded on 1st of January (see Tables S2). Number of fires: 5,197 (2006-2025).

Mountain	Period (years)	Fires (n)	Peak month	Peak share (%)	Median month
Alps	2006-2014	5	Jul	60.0	Jul
Alps	2015-2025	91	Mar	26.4	Mar
Apennines	2006-2014	153	Aug	50.3	Aug
Apennines	2015-2025	341	Aug	44.6	Aug
Balkans/Southeast Europe	2006-2014	501	Aug	49.1	Aug
Balkans/Southeast Europe	2015-2025	2141	Mar	33.5	Apr
Carpathians	2006-2014	6	Aug	50.0	Aug
Carpathians	2015-2025	82	Mar	54.9	Mar
French/Swiss middle mts.	2015-2025	30	Feb	46.7	Feb
Iberian mts.	2006-2014	285	Aug	31.6	Aug
Iberian mts.	2015-2025	610	Mar	32.0	Mar
Pyrenees	2006-2014	3	Mar	100.0	Mar
Pyrenees	2015-2025	160	Feb	59.4	Feb
Turkey	2006-2014	17	Aug	64.7	Aug
Turkey	2015-2025	772	Aug	34.5	Aug

Table S12. Comparison of seasonal fire timing between 2006–2014 and 2015–2024 across mountain regions. The table reports the number of fires per period, peak month and its share, median fire month, and the shift in peak and median timing (in months; negative values indicate earlier fire occurrence). Negative values indicate earlier fire occurrence. The forest fires from 2000 to 2005 were removed since many fires do not have exact date, hence were recorded on 1st of January (see Tables S2). Number of fires: 5,197 (2006-2025).

Mountain	Number of fires 2006-2014	Peak month 2006-2014	Peak share (%) 2006-2014	Median month 2006-2014	Number of fires 2015-2025	Peak month 2015-2025	Peak share (%) 2015-2025	Median month 2015-2025	Peak month shift (months)	Median month shift (months)
Alps	5	Jul	60	Jul	91	Mar	26.4	Mar	-4	-4
Apennines	153	Aug	50.3	Aug	341	Aug	44.6	Aug	0	0
Balkans/Southeast Europe	501	Aug	49.1	Aug	2141	Mar	33.5	Apr	-5	-4
Carpathians	6	Aug	50	Aug	82	Mar	54.9	Mar	-5	-5
French/Swiss middle mts.	NA	NA	NA	NA	30	Feb	46.7	Feb	NA	NA
Iberian mts.	285	Aug	31.6	Aug	610	Mar	32	Mar	-5	-5
Pyrenees	3	Mar	100	Mar	160	Feb	59.4	Feb	-1	-1
Turkey	17	Aug	64.7	Aug	772	Aug	34.5	Aug	0	0

Statistical significance of seasonal shifts

Table S13. Statistical significance of seasonal shifts in fire occurrence between 2006–2014 and 2015–2024 by mountain region. P-values derive from tests comparing monthly fire distributions between periods. The forest fires from 2000 to 2005 were removed since many fires do not have exact date, hence were recorded on 1st of January (see Tables S2). Number of fires: 5,197 (2006-2025).

Mountain	P value (seasonal shift)
Alps	$p = 2.74 \times 10^{-3}$
Apennines	$p = 2.90 \times 10^{-4}$
Balkans/Southeast Europe	$p < 1.13 \times 10^{-118}$
Carpathians	$p = 2.93 \times 10^{-8}$
French/Swiss middle mts.	$p = 1.31 \times 10^{-6}$
Iberian mts.	$p = 3.48 \times 10^{-36}$
Pyrenees	$p = 0.38$
Turkey	$p = 0.76$

Table S14. Cyclic seasonal mixed model of fire elevation

Model: Elevation ~ sin(Month) + cos(Month) + (1 | Mountain region)

Number of fires: 5,197 (2006-2025). The forest fires from 2000 to 2005 were removed since many fires do not have exact date, hence were recorded on 1st of January (see Tables S2). Mountain regions: 8.

The seasonal cycle corresponded to an estimated peak-to-trough shift of approximately 170 m in mean fire elevation, suggesting a systematic upslope expansion of fire occurrence during the warm season. Because Month is circular, the amplitude of seasonal elevation change is:

Amplitude = $\sqrt{(\sin^2 + \cos^2)}$

= $\sqrt{(47.47^2 + 69.84^2)}$

≈ $\sqrt{(2253 + 4878)}$

≈ $\sqrt{7131}$

≈ 84.4 m

The peak-to-trough difference is twice the amplitude: $2A \approx 2 \times 84.4 \approx 168.8$ m ≈ 170 m

Fixed effects

Term	Estimate	SE	t value	Interpretation
Intercept	997.8	44.8	22.30	Mean annual elevation (m)
sin(Month)	−47.47	5.06	−9.38	Seasonal component
cos(Month)	69.84	8.44	8.27	Seasonal component

Component	Variance	SD
Mountain intercept	15,443	124.3 m
Residual	72,497	269.3 m

6. Vegetation composition across fire region types in European mountains

Table S15. Vegetation composition across fire region types. Distribution of burned area (ha) and median vegetation cover (%) across elevation classes (≤1,400 m and >1,400 m) within the three fire regime types (high and persistent, moderate and reoccurring, low and emerging) for the period 2000–2025. Vegetation classes represent percentage cover within burned polygons.

Region type	Elevation class (m)	Total area (ha)	Conifer forest (%)	Broadleaf forest (%)	Mixed forest (%)	Transi-tional forest (%)	Total forest cover (%)
High and persistent	<=1,400 m	2132902	8.41	17.47	5.54	40.9	72.32
High and persistent	>1,400 m	108354	7.96	12.53	5.24	55.48	81.21
Low and emerging	<=1,400 m	39968	9.65	43.06	9.55	15.9	78.16
Low and emerging	>1,400 m	5918	22.25	13.16	17.04	25.12	77.57
Moderate and reoccurring	<=1,400 m	506620	25.91	6.54	4.85	42.03	79.33
Moderate and reoccurring	>1,400 m	54831	4.58	26.09	2.33	50.32	83.32

Table S16. Vegetation composition across mountain regions and elevation classes. Distribution of fire occurrence, burned area, and vegetation type across mountain regions and elevation classes (≤1,400 m and >1,400 m considering mean fire elevation) for the period 2000–2025. Vegetation variables represent percentage cover of conifer, broadleaf, mixed forest, and transitional vegetation within burned polygons. Fire (%) and Area (%) indicate the proportion of total fires and burned area within each mountain region attributed to the respective elevation class. "Total forest cover %" represents the combined percentage of the four vegetation classes within burned areas.

Mountain	Elevation class (m)	Fire number	Total area (ha)	Conifer forest (%)	Broadleaf forest (%)	Mixed forest (%)	Transitional forest (%)	Fire (%)	Area (%)	Total forest cover %
Alps	<=1,400 m	100	15291	8.29	43.01	13.09	17.71	84.75	73.64	82.1
Alps	>1,400 m	18	5473	22.58	12.91	17.91	23.8	15.25	26.36	77.2
Apennines	<=1,400 m	574	126202	12.99	28.59	7.99	24.91	97.45	99.05	74.48
Apennines	>1,400 m	15	1211	3.72	71.83	4.79	3.32	2.55	0.95	83.66
Balkans/Southeast Europe	<=1,400 m	2480	967050	5.74	27.03	6.82	33.54	89.43	92.17	73.13
Balkans/Southeast Europe	>1,400 m	293	82174	6.68	12.7	6.4	53.61	10.57	7.83	79.39
Carpathians	<=1,400 m	91	17726	2.67	55.35	4.21	11.03	95.79	98.03	73.26
Carpathians	>1,400 m	4	357	22.54	0.28	7.84	50.01	4.21	1.97	80.67
French/Swiss middle mts.	<=1,400 m	52	6951	30.45	11.87	15.38	24.33	98.11	98.75	82.03
French/Swiss middle mts.	>1,400 m	1	88	1.11	81.11	0	6.67	1.89	1.25	88.89
Iberian mts.	<=1,400 m	1352	1039650	10.33	7.23	4.06	49.68	91.54	97.65	71.3
Iberian mts.	>1,400 m	125	24969	12.37	9.11	1.42	64.14	8.46	2.35	87.04
Pyrenees	<=1,400 m	217	34317	7.34	31.74	2.32	42.44	93.13	96.77	83.84
Pyrenees	>1,400 m	16	1147	26.42	9.98	3.33	49.2	6.87	3.23	88.93
Turkey	<=1,400 m	609	472303	27.26	4.71	5.04	42.01	68.66	89.79	79.02
Turkey	>1,400 m	278	53684	4.12	26.43	2.31	50.34	31.34	10.21	83.2

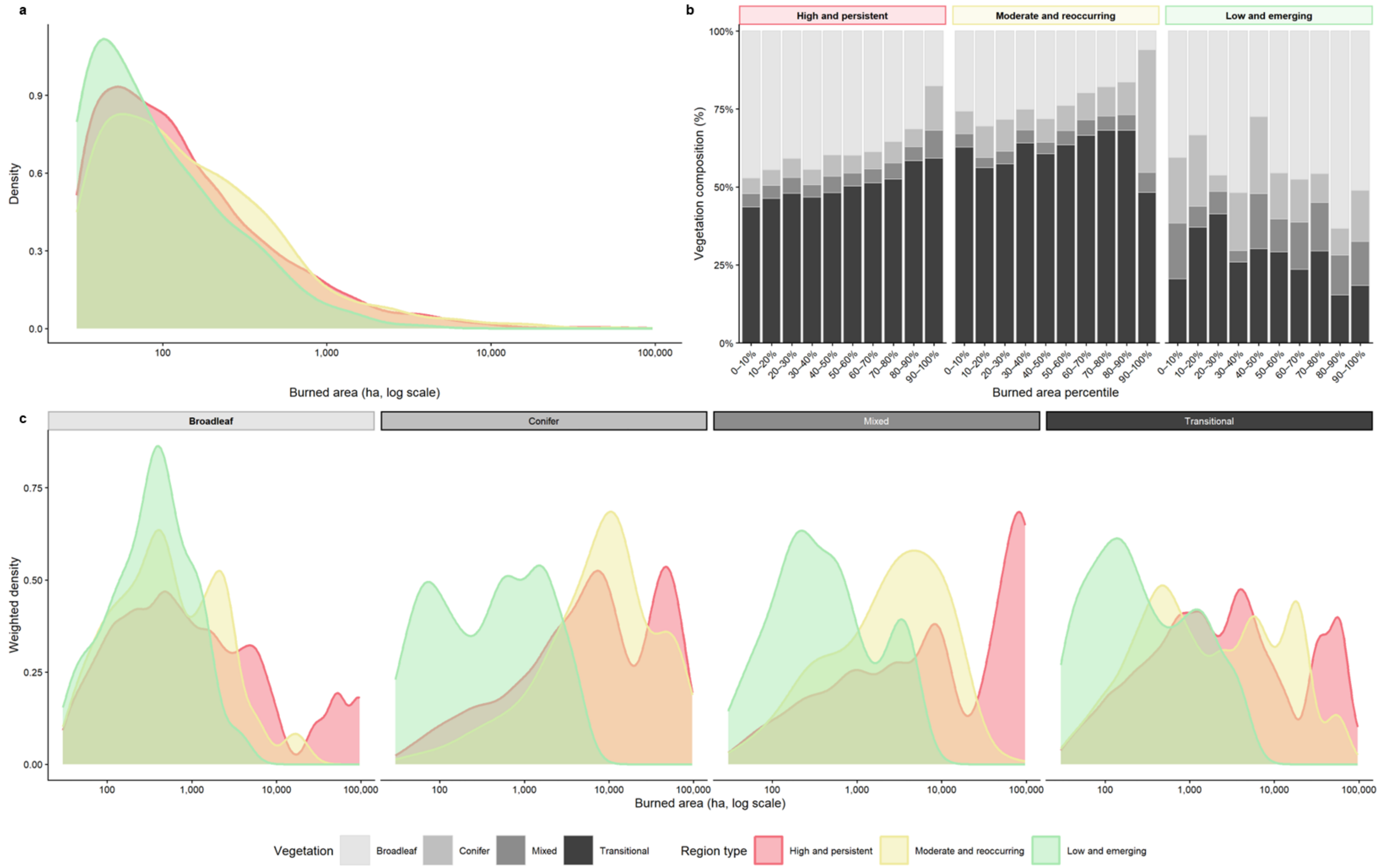

Fig. S6. Burned area distributions and vegetation composition across region types. a Probability density of burned area (log scale) for high and persistent (red), moderate and reoccurring (yellow), and low and emerging (green) fire region types. **b** Vegetation composition of burned areas across burned area percentiles (0–10% to 90–100%) separated by region type, showing proportional

contributions of broadleaf, conifer, mixed, and transitional forests. **c** Weighted density of burned area (log scale) for each vegetation type (broadleaf, conifer, mixed, transitional), stratified by region type. Overall, burned area distributions are right-skewed with large fires occurring across all region types, but the contribution of vegetation classes varies: transitional forests dominate across percentiles, while broadleaf and conifer contributions differ by region type and fire regime intensity.

7. Climatic drivers

Table S17. Relative contribution of climatic, vegetation, and human predictors to burned area. Inclusive R^2 and overall model fit for the Europe-wide mixed effects model (M1). Inclusive R^2 values represent the proportion of variance in log-transformed burned area uniquely attributable to each predictor within the full mixed model. Estimates are based on marginal R^2 partitioning (fixed effects only) using bootstrapped confidence intervals (95% CI). The marginal R^2 ($R^2m = 0.171$) indicates the proportion of variance explained by the fixed effects, whereas the conditional R^2 ($R^2c = 0.207$) reflects the variance explained by both fixed effects and the mountain-region random intercept. Percent contributions are calculated relative to the marginal R^2.

ID	Predictor	Inclusive R^2	95% CI Lower	95% CI Upper	% of R^2m
1	Vapor pressure deficit	0.096	0.090	0.114	55.9
2	Soil moisture	0.060	0.054	0.071	34.9
3	Precipitation	0.032	0.027	0.039	18.8
4	PDSI	0.002	3.43×10^{-4}	0.004	0.9
5	Actual evapotranspiration	0.054	0.043	0.066	31.4
6	Wind speed	0.015	0.011	0.021	8.5
7	Human footprint	1.35×10^{-5}	8.06×10^{-7}	0.001	0.0
8	Broadleaf forest	0.037	0.031	0.049	21.5
9	Mixed forest	1.67×10^{-4}	1.08×10^{-5}	0.002	0.1
10	Coniferous forest	0.004	0.001	0.007	2.1
11	Transitional forest	0.002	0.001	0.004	1.0

Table S18. Elevational breakpoints of climatic, vegetation, and human variables across European fire regimes. Elevational breakpoints (cutpoints, m) derived from statistical change-point analyses for climatic, fuel, and human variables across fire occurrences in Europe and within each fire regime type (high and persistent, moderate and reoccurring, low and emerging). For each variable, the table reports the estimated elevation cutpoint, test statistic, and associated p-value. The number of observations per regime is shown at the top. Summary statistics (median, average, and standard deviation of cutpoints) are provided at the bottom.

	**Europe**			**High and persistent**			**Moderate and reoccurring**			**Low and emergent**		
No. observations	5,608			4,270			1,087			251		
	cutpoint	*statistic*	*p_value*	*cutpoint*	*statistic*	*p_value*	*cutpoint*	*statistic*	*p_value*	*cutpoint*	*statistic*	*p_value*
AET	**1,657**	9.8346	0	**925**	3.3399	0.0222	**957**	4.1869	0.0010	**1,695**	2.8398	0.0698
DEF	**1,601**	9.1860	0	**980**	7.1862	0	**1,836**	2.5333	0.1652	**1,411**	3.2977	0.0154
PDSI	**992**	2.8765	0.0750	**992**	4.6947	0.0004	**957**	1.7922	0.5961	**1,380**	2.0660	0.3713
PET	**1,568**	7.5670	0	**1069**	7.1787	0	**1,836**	2.6369	0.1346	**1,181**	2.1651	0.3083
PPT	**1,610**	9.0364	0	**930**	7.0240	0	**1,357**	5.1746	0	**1,677**	2.7878	0.0726
Soil moisture	**1,567**	11.9949	0	**1566**	6.6486	0	**1,658**	4.0971	0.0012	**1,411**	5.8187	0
tmax	**1,568**	6.2170	0	**1069**	11.1337	0	**1,843**	4.5129	0	**1,159**	1.1060	0.9580
tmin	**1,004**	4.3344	0.0006	**1069**	10.4911	0	**1,843**	4.3944	0.0002	**1,370**	2.4328	0.1710
VPD	**1,568**	9.2247	0	**980**	9.2417	0	**963**	3.4952	0.0142	**1,411**	2.4501	0.1748
Wind speed	**1,567**	9.1455	0	**1563**	3.1376	0.0366	**1,649**	7.4136	0	**877**	2.4714	0.1570
Broadleaf forest	**1,640**	9.0254	0	**1592**	7.5897	0	**1,231**	5.8405	0	**1,181**	6.2439	0
Coniferous forest	**1,435**	5.8668	0	**1564**	8.5614	0	**958**	5.1057	0	**1,210**	6.7422	0
Mixed forest	**1,307**	6.5059	0	**1551**	7.9363	0	**1,788**	3.5410	0.0108	**1,252**	4.1144	0.0004
Transitional forest	**1,681**	7.3190	0	**1593**	4.0181	0.0020	**966**	3.1383	0.0366	**1,395**	3.8416	0.0034
Human footprint	**1,101**	18.2473	0	**1105**	18.2599	0	**1,618**	7.7302	0	**1,550**	2.0084	0.4109
Median	**1,568**			**1,069**			**1,618**			**1,380**		
Average	**1,458**			**1,236**			**1,431**			**1,344**		
Standard deviation	**239**			**288**			**386**			**212**		

8. Variable selection

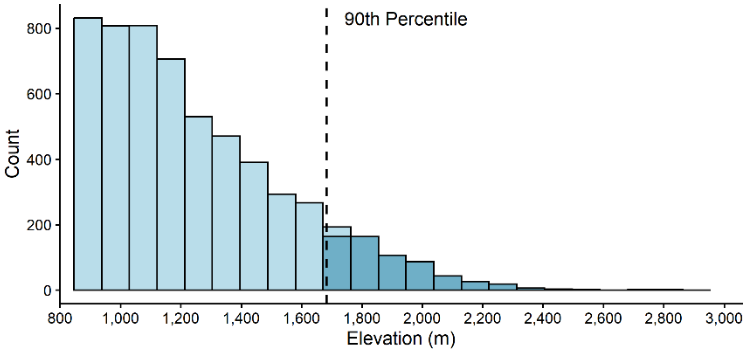

Fig. S7. Illustration of fire selection based on the 90th percentile of the elevation distribution. The histogram represents the elevation distribution of fires above 800 m. The dashed line represents the elevation at the 90th percentile of the fire elevation distribution (Z_{90}). Light blue bars represent the counts of fires below the Z_{90} threshold and darker blue bars represent fires above it.

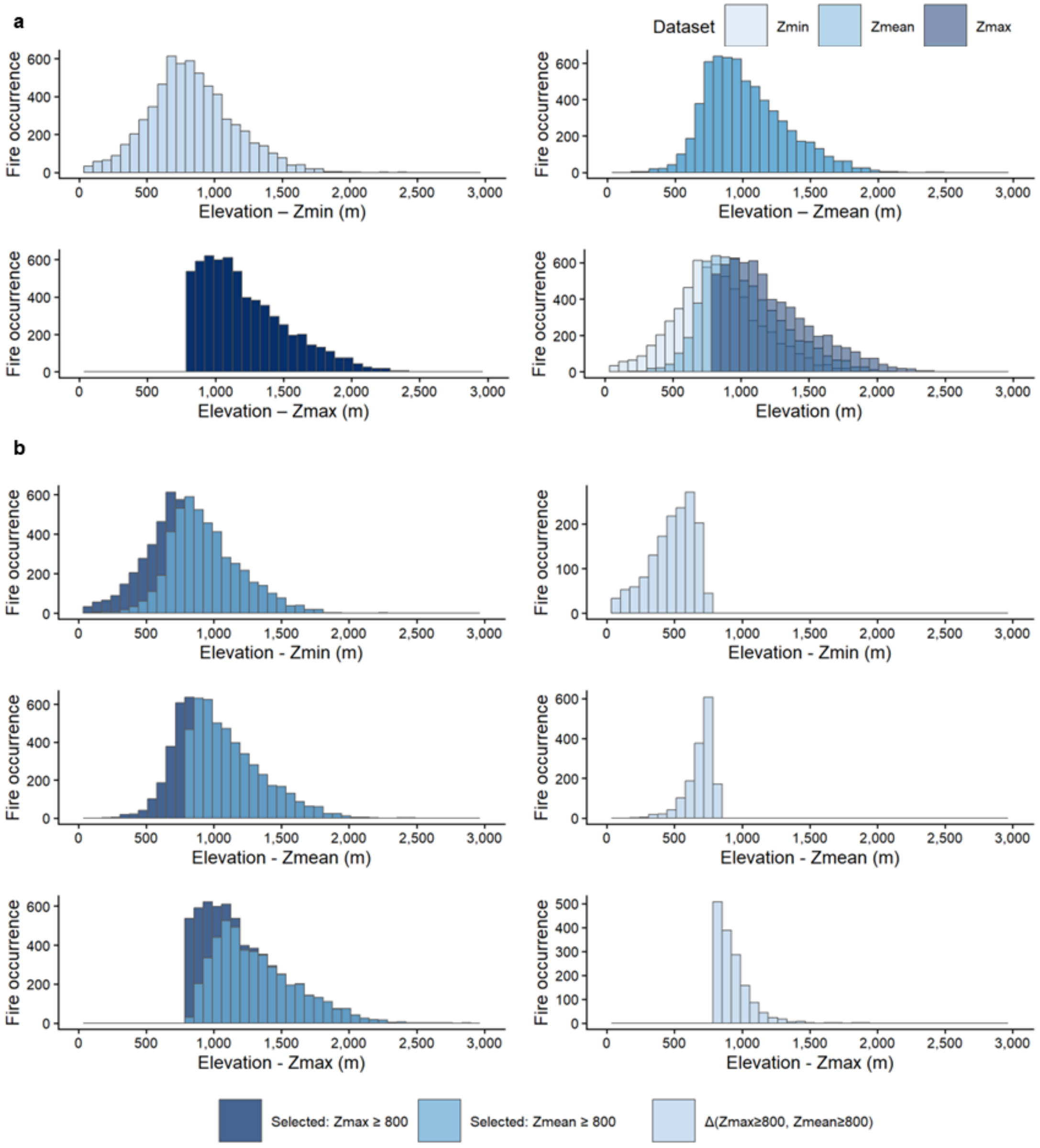

Fig. S8. Elevation distributions and threshold-based selection effects. a Histograms of minimum (Zmin), mean (Zmean), and maximum (Zmax) fire elevations and their overlap (0–3,000 m). **b** Elevation distributions after applying an 800 m threshold. Dark blue indicates fires with Zmax ≥ 800 m, medium blue those with Zmean ≥ 800 m, and light blue the symmetric difference between both selections.

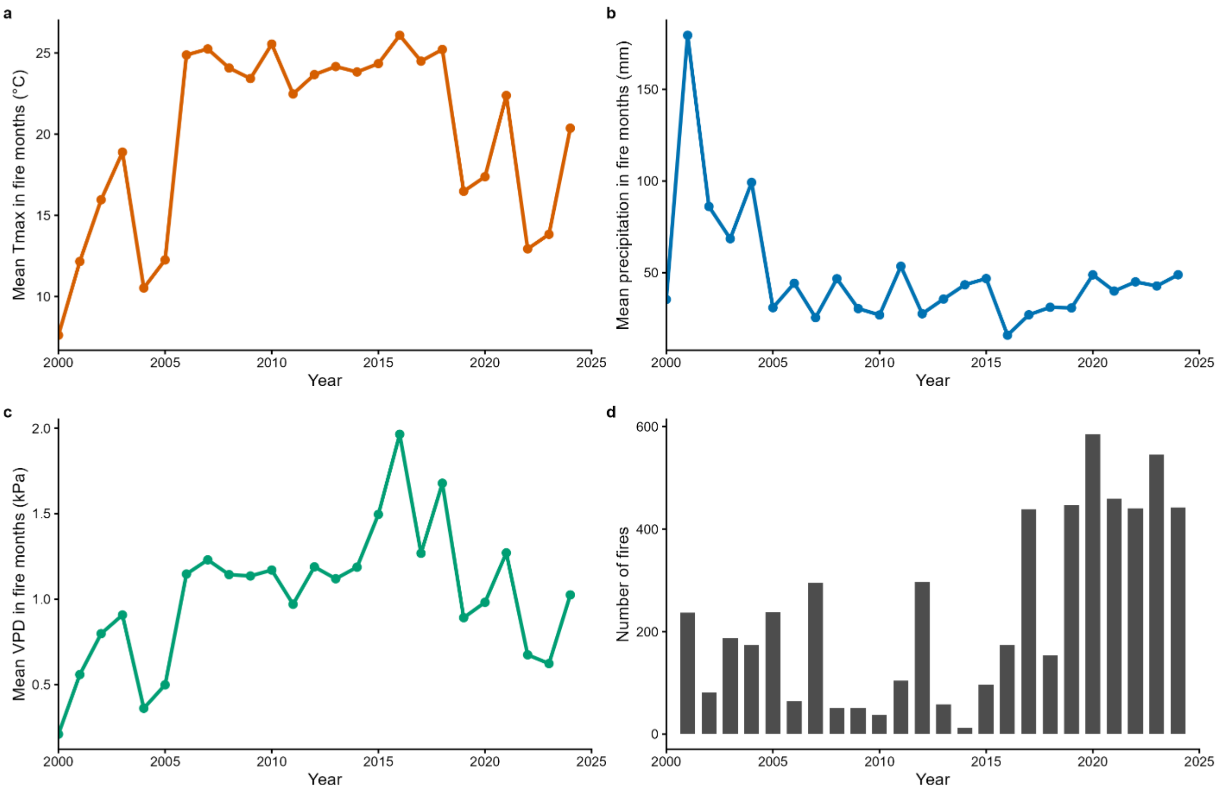

Fig. S9. Interannual variation in fire-month climate conditions and fire activity. a Annual mean monthly maximum temperature (Tmax), **b** annual mean monthly precipitation total (PPT), and **c** annual mean vapor pressure deficit (VPD) calculated for the months in which individual fires occurred, averaged across all fire polygons within each year. **d** Number of recorded fires per year. Monthly climate variables were derived from TerraClimate and processed as follows: TerraClimate fields at approximately 4 km resolution were bilinearly resampled to a 1 km grid defined by a digital elevation model (DEM). Temperature variables were additionally downscaled using a lapse-rate correction (-0.0065 °C m^{-1}) based on the difference between local 1 km DEM elevation and a 4 km mean-elevation surface, to account for topographic effects. For each fire polygon and fire date, climate values from the corresponding calendar month were extracted as the spatial mean across all grid cells intersecting the polygon. Precipitation represents monthly totals (mm $month^{-1}$), whereas temperature and VPD represent monthly means. Annual values shown here are averages across all fire events within each year. Years without available TerraClimate data (e.g., 2025) are excluded from climate summaries.

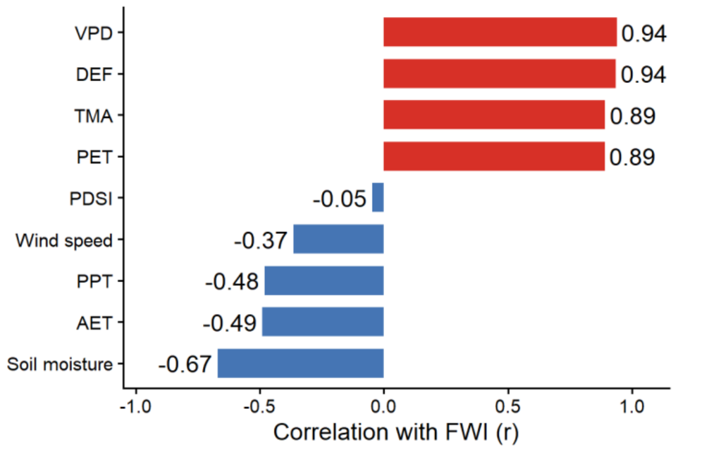

Fig. S10 Correlation of the Canadian Fire Weather Index (FWI) with mean climatic variables (2000 to 2024).

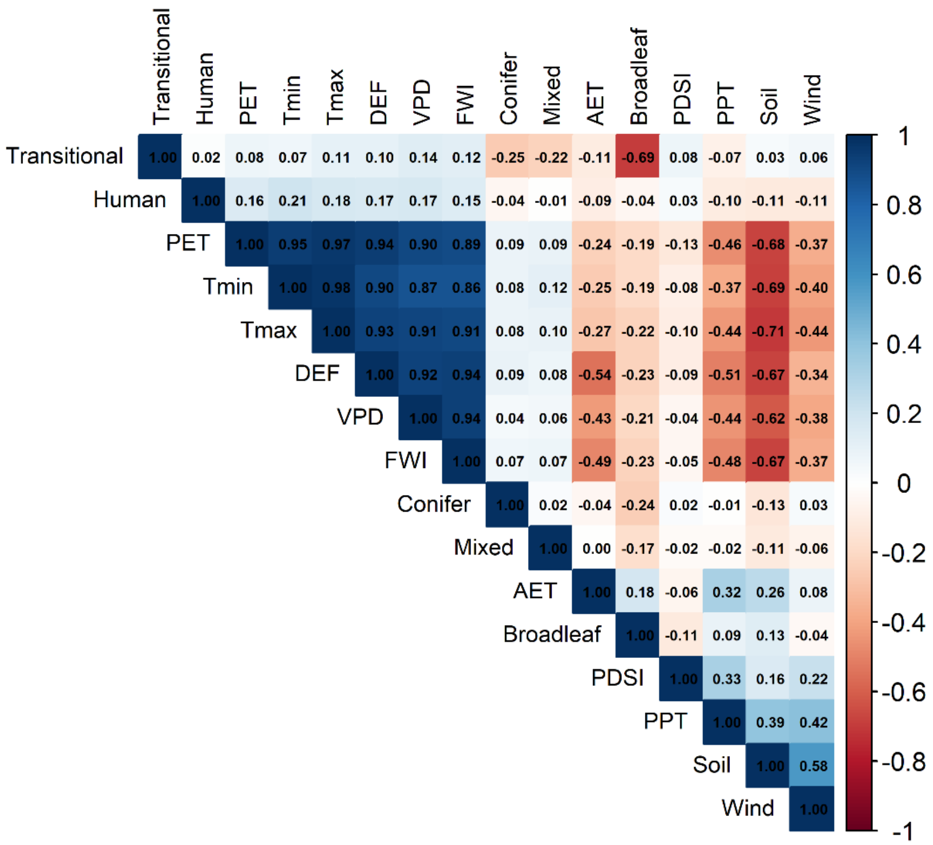

Fig. S11 Correlation matrix of climatic, fuel, and human variables across fire events. Strong positive correlations are observed among atmospheric demand variables (VPD, DEF, PET, Tmax), while soil moisture and precipitation are negatively correlated with these variables. The Fire Weather Index (FWI) shows strong positive correlations with VPD and DEF and negative correlations with soil moisture and precipitation, indicating that it integrates key aspects of climatic fire risk.
