# Supplementary material for: Climate-driven upward spread of forest fires in European mountain regions
Source: Nat Commun. 2026 Apr 30;17:5912. doi: 10.1038/s41467-026-72551-0 (PMC13338238; doi:10.1038/s41467-026-72551-0)
Supplement: Supplementary file 2 — Transparent Peer Review file [file 41467_2026_72551_MOESM2_ESM.pdf]

nature portfolio

Peer Review File

Climate-driven upward spread of forest fires in European mountain regions

Corresponding Author: Dr Mirela Beloiu

This file contains all reviewer reports in order by version, followed by all author rebuttals in order by version.

Version 0:

Reviewer comments:

Reviewer #1

(Remarks to the Author)
This manuscript addresses a highly relevant and timely question: whether climate change is enabling an upslope expansion of forest fires across European mountain regions. Using EFFIS fire records, climatic time series, and mixed statistical modelling, the authors demonstrate a clear post-2015 increase in high-elevation fire occurrence, with atmospheric aridity (notably VPD) emerging as the dominant driver of burned area.
The study is ambitious, methodologically solid, and contributes valuable continental-scale evidence.
However, several aspects require clarification and methodological refinement before the manuscript can be considered for publication. In particular, (i) the analysis would benefit from integrating a composite fire weather index such as FWI, (ii) high-resolution climatic data should be considered for mountainous terrain, and (iii) the representation of anthropogenic drivers is too limited.

Major comments:

Integration of fire weather indices (FWI)
The study convincingly identifies VPD as the dominant climatic predictor of burned area, but the analysis treats climatic variables independently, overlooking their non-linear and synergistic interactions.
I strongly recommend testing whether the Canadian Fire Weather Index (FWI)—already operational in EFFIS and Copernicus systems—better explains burned area and elevation patterns than the current suite of single variables (VPD, PPT, SM, PET, etc.) avoiding variables collinearity.
FWI integrates temperature, humidity, wind, and precipitation into a standardized measure of fire danger, enabling a consistent comparison across regions and years and aligns with established European fire risk frameworks.

Climate data resolution and anthropogenic variables
The Discussion should more explicitly address the influence of data resolution and the limited representation of anthropogenic factors.
The use of TerraClimate at 4 km resolution likely obscures the strong topoclimatic variability typical of mountainous regions, where temperature, humidity, and wind change sharply over short distances. I suggest clarifying this limitation and, where possible, incorporating or testing higher-resolution climatic datasets (e.g., easyclimate at 1 km^2 - https://doi.org/10.1016/j.envsoft.2023.105627) or applying a simple downscaling approach (lapse-rate correction, topographic bias adjustment) to better capture fine-scale climate–fire interactions. This would improve the accuracy of elevational analyses and strengthen the interpretation of climate–fire linkages.
In parallel, the manuscript's treatment of human influence is overly simplified.
The "Human Footprint Index" provides only a coarse, static proxy that underestimates key drivers such as ignition pressure, land-use change, and accessibility.
The authors should consider including additional variables (road density, wildland–urban interface, agricultural abandonment, tourism intensity) or, at minimum, explicitly acknowledge this limitation to balance the current climate-dominant narrative.

Justification of the 1,400 m elevation threshold
The 1,400 m threshold is statistically derived but lacks ecological or climatic justification.
Is this value consistent across mountain systems, or does it reflect distinct bioclimatic zones?
A sensitivity analysis (e.g., thresholds between 1,200 – 1,600 m) or modelling elevation as a continuous effect could bring more robustness.

Temporal discontinuity post-2015
The sharp post-2015 increase in fire activity is compelling, but it may partly reflect methodological changes in the EFFIS data stream rather than a purely climatic signal.
In 2018, EFFIS transitioned from MODIS (250 m) to Sentinel-2 (20 m) imagery for burned area mapping, which markedly improved spatial resolution and enabled the detection of fires smaller than 30 ha—precisely those excluded under the ≥ 30 ha threshold applied in this study.
With this technological shift, the effective minimum mapping unit changed, potentially biasing total burned area estimates and temporal trend analyses.
Therefore, the authors should verify whether the apparent post-2015 sharp increase in burned area and fire frequency correlates with the sensor transition. If possible, they should provide evidence that the observed peak is not an artifact of improved detectability or processing differences between MODIS and Sentinel-2 datasets.

Minor comments
1. In the abstract, the authors could quantify the rate of upslope shift (e.g., “median elevation increased by X m per decade”) to convey magnitude.
2. In Figure 1, I recommend improving the visual interpretability of the map by converting the burned-area polygons into point representations (e.g., centroids), to reduce overlap. Given that European mountain ranges are already well known, the authors could simply shade the mountainous areas in obscure-grey to provide spatial context without distracting from the main signal. Additionally, colour gradients could then be used to represent quinquennial periods of fire occurrence, effectively conveying both spatial and temporal dynamics of fire distribution across the continent.

(Remarks on code availability)

Reviewer #2

(Remarks to the Author)
The study by Beloui et al. analyses the upward shift of wildfires in mountainous areas across southern and central Europe. The research gap and objectives of the study are clearly stated and of interest, as they focus on forests that are generally not fire-prone and thus, more vulnerable to fire-related mortality and regeneration failure, putting the ecosystem services they provide at risk. The authors used remote sensing and climatic time series from 2000 to 2022 to study the upward shift of wildfires in mountains during the last two decades. They used forest fire records from the EFFIS database (burned area > 30 ha) across eight mountain regions and analysed those that burned above 800 m elevation using climate, forest type and human footprint as predictors.
Overall, the manuscript is well written and results interesting, but analyses are not always properly justified and the discussion lacks critical reflection. I believe that after revision it can be considered for publication by Nature Communications.
My main concerns relate to the methodology:
- Elevation threshold for including wildfires: The authors included in the analysis all wildfires whose maximum elevation was > 800 m, which includes a substantial number of fires that actually originate below the threshold and only burn a small portion above 800 m. This choice is not explained in the manuscript and it is not clear why the authors chose the maximum elevation instead of the mean elevation as the threshold to include fires, which seems more appropriate. A quick scan of the dataset shows that almost 25% of the fires have a mean elevation below 800 m, and these fires account for 44% of the total burned area. If the authors have valid reasons for their approach, it should be clearly stated that these fires do not occur entirely above 800 m. It would also be useful to include the mean elevation of fires in a supplementary table, broken down by mountain region, for example.
- Statistical models: The justification for the statistical models is not clearly explained. I provide more specific comments on this below.
Regarding the discussion, a deeper reflection is needed on how burned area is not only influenced by climatic conditions and the type of vegetation cover, but highly related to the amount of fuel load and the topography (e.g., slope plays a critical role and it is not addressed in the current manuscript).

In the following I provide more detailed comments.
**Abstract
I would appreciate more details of the study in the abstract. For example, type of vegetation cover and human footprint are

not mentioned as part of the analysis, or number of mountain ranges evaluated.
L17. It depends on the region, but a 30 ha fire is typically not considered large. I would suggest to just state "forest fires > 30 ha".

**Introduction
L33. Since this paper focuses on Europe, why don't you mention Europe as an example? For example, in Portugal in 2017 there were extreme wildfires that burned large areas and even caused fatalities (Turco et al., 2019).
L35. The concept of megafires is ambiguous, as it is related to the size of the fire. However, climate change is not only increasing fire size but also the amount of energy that fires release. A single fire may not be particularly large but release huge amounts of energy, spread erratically and cause severe damage. That's why this concept is increasingly being replaced by "extreme wildfire events" (e.g., Tedim et al., 2018; Stoof et al., 2024; Linley et al., 2022).
L38. Fire suppression may have reduced burned areas in the late 20th century, but it has also contributed to increase fuel load, increasing the likelihood of larger and more intense wildfires, the so-called fire paradox (e.g., Kreider et al., 2024). This aspect should be acknowledged in the sentence.
L42. "central and northern Europe will be affected due to high fuel availability, warming, and drought" – this sentence is not entirely correct, since fuel availability depends largely on temperature and relative humidity. It would be more precise to state "due to high fuel load, which becomes increasingly available under warm and dry conditions" or "due to high fuel availability as a consequence of increasing temperatures and drought conditions", or similar.
L42. "Yet, it remains unclear whether current fire activity already signals a sustained upslope expansion into less-adapted forests" – that's not entirely accurate. There is evidence suggesting these trends. The topic may be understudied, but several studies have already analysed these patterns, and some of them are cited in your manuscript.
L46. "Moisture content" instead of "moisture availability".
L51. Interplay between ignitions, fuel, climate, AND TOPOGRAPHY.
L56. One of the cited studies in this sentence (Dupire et al., 2017) refers to the French Alps, not to southern Europe.
L61. I suggest avoiding the use of the adjective large. Moreover, most mountain fires burn less than 30 ha. It is OK to limit the analyses to fires > 30 ha because this is the threshold in EFFIS database, but it should be acknowledged that this approach excludes most of the fires that burn in mountain areas (for example, in the Alps most wildfires are < 10 ha – Conedera et al., 2018). An increase in fire activity of fires < 30 ha is also likely and probably already happening – this limitation should be also acknowledged in the manuscript.

**Results
L80. The caption of Figure 1 should be clarified. As it stands, it gives the impression that the mountain regions shown on the map correspond to areas above 800 m, but the authors actually mean that the figure displays the fire polygons that burned above 800 m within the mountain regions defined by Kapos et al. (2000). Moreover, in Figure 1b, it is somewhat confusing that the number of fires is below 1,000 while the y-axis extends up to 3,000. It would be clearer if the axis range matched the data range.
L93. Table S2 does not show a trend in fire occurrence. It shows the total number of fires by elevation class and mountain region.
L96. Figure 2b. The distribution of fires in the D800 dataset seems quite similar. How do you explain the significant p-value?
L103. This reference should probably refer to Table S2, since Tables S3 and S4 do not show fire occurrences by mountain region.
L119. The percentage cover of each vegetation type below and above 1400 m would help the reader to better interpret the results.
L123. Table 1. I suggest reporting p-values as $p < 0.001$ for clarity. The parameters that are not significantly different between below and above 1400 m should not be highlighted in bold (Tmin, human footprint). Moreover, units of climatic variables are missing and should be included.
L135. It should be "fuel type" or "type of vegetation cover". Just "fuel" can be misleading, as one could interpret it as referring to fuel load.
L147. Figure 4. Lower panels should be (d) instead of (a). Soil moisture in moderate and reoccurring fire regimes is positively correlated with burned area, any idea why?

**Discussion
L149. "Fires climb higher" sounds a bit awkward. A clearer alternative could be "fires shift upslope" or a similar expression. Moreover, based on your analysis, how do you know that fires are intensifying? An increase in burned area does not necessarily imply higher fire intensity.
L153. Delete "after 2015", already mentioned before.
L158. Did you mean (800-1400 m) instead of (800-1600 m)?
L164. Socioeconomic drivers are represented by the human footprint parameter, which did not show any significant contribution driving fires upslope. I think that claiming that this study provides a framework that links fire activity to socioeconomic drivers seems overstated.
L167. Careful with terminology ("severe fires"). Fire severity refers to fire effects on the ecosystem; fire intensity is the energy released by the fire. In this study burned area and fire occurrence are analysed, and they do not always relate to intensity or severity (Keeley, 2009).
L172. "Extreme fire weather" is more commonly used than "severe fire weather"
L176. The analysis shows a minor role of vegetation composition in driving fires upslope. But the analysis did not include fuel load and structure as potential predictors of burned area. It should be acknowledged in the discussion.
L198. Increasing fuel load/continuity/cover?
L214. Besides silvicultural treatments, prescribed burning can also be a valuable tool to reduce fuel load and continuity and increase resistance to wildfires (e.g., Vilà-Vilardell et al., 2023).

L215. Reduce fuel continuity AND LOAD.
L216. Instead of “coordinated fuel management”, a term which I believe is not much used, you could refer to “integrated fire management” (e.g., Oliveras-Menor et al., 2025).
L225. The statement that fire activity has intensified is misleading, as this study did not analyse changes in fire intensity. To avoid misinterpretation, I suggest changing it to “fire activity has increased”.
L231. Fine-scale monitoring of what?

**Methods
L260. Why did you exclude the small mountain ranges from the analysis?
L287. The resolution of the climate data is quite coarse (4 km, downscaled to 1 km). This should be acknowledged as a limitation in the discussion, particularly because fine-scale climatic variability is common in mountain regions with complex topography (e.g., Schuck et al., 2011).
L288. The authors analyse fire occurrences and burned area using average yearly climate values. This approach is appropriate, but a deeper analysis of the seasonal distribution of fires would be valuable, as fire timing is likely to influence post-fire regeneration (e.g., Tangney et al., 2022). When do most fires occur in each mountain region, and has this timing shifted over the past two decades? Including a summary table would be interesting to show.
L289. The human footprint parameter is not clearly explained in the manuscript. Which variables were used to create this map? This information is important to interpret the results. Also, in Table 2 this variable is shown as unitless, but what is its range? More details should be provided, as this is not a commonly known parameter, at least within the wildfire community.
L307. The significant intensification of fire activity is based on which data source? Is it derived from your dataset? This should be clearly stated.
L308. How were fires classified within the elevation classes? Based on the mean elevation of each fire or the maximum? This should be specified, especially since the 800 m threshold to include wildfire in the analysis was defined using the maximum elevation.
L309. From Table S3, I see that Year was included as a numeric predictor. This should be clearly explained and justified in the methods section. Using Year as a numeric predictor implies a linear relationship between fire occurrences and time, which may not always be the case. Thus, the choice to include Year as numeric should either be properly justified or Year should be included as a categorical factor. Also, Year was included as a fixed effect, but also as a random slope within Elevation class. This should also be explained and justified. Additionally, which GLMM family did you use? And why was “mountain range” not included as a random effect? How did you account for the dependency of the data related to location?
L320. For the GAM, same as with the previous model, what is the rationale for including Year as a numeric variable? Why did you choose GAM and not GAMM with a random intercept that accounts for dependency, instead? Moreover, where are the numerical results of this model?
L334. The rationale to choose 1400 m as a threshold should be more clearly justified.
L339. Parenthesis missing after transitional.
L350. Why burned area was log-transformed? Did you test with other GLMM families rather than Gaussian? Log-scaling is not optimal, since it changes the relationship between the response and predictor variables (Zuur et al., 2009, 2010). And again, why not including mountain range as random intercept?
L357. The term “region type” is confusing, as it suggests mountain regions. Instead, I recommend replacing it by “fire regime type”.

**Supplementary material
Table S1. For clarity, I suggest changing the column order of the mountain ranges to match the order used for the fire regime types (red-green-yellow; red-green-yellow)
Figure S2. Labels of figures are missing (a to f).
Table S4. Where do these results come from? It is not clear.

REFERENCES
Conedera, M., Krebs, P., Valese, E., Cocca, G., Schunk, C., Menzel, A., ... & Pezzatti, G. B. (2018). Characterizing Alpine pyrogeography from fire statistics. Applied Geography, 98, 87-99.
Keeley, J. E. (2009). Fire intensity, fire severity and burn severity: a brief review and suggested usage. International journal of wildland fire, 18(1), 116-126.
Kreider, M.R., Higuera, P.E., Parks, S.A. et al. Fire suppression makes wildfires more severe and accentuates impacts of climate change and fuel accumulation. Nat Commun 15, 2412 (2024). https://doi.org/10.1038/s41467-024-46702-0
Linley, G. D., Jolly, C. J., Doherty, T. S., Geary, W. L., Armenteras, D., Belcher, C. M., ... & Nimmo, D. G. (2022). What do you mean,‘megafire’?. Global Ecology and Biogeography, 31(10), 1906-1922.
Oliveras Menor, I., Prat-Guitart, N., Spadoni, G. L., Hsu, A., Fernandes, P. M., Puig-Gironès, R., ... & Armenteras Pascual, D. (2025). Integrated fire management as an adaptation and mitigation strategy to altered fire regimes. Communications Earth & Environment, 6(1), 202.
Schunk, C., Wastl, C., Leuchner, M., Schuster, C., & Menzel, A. (2013). Forest fire danger rating in complex topography– results from a case study in the Bavarian Alps in autumn 2011. Natural hazards and earth system sciences, 13(9), 2157-2167.
Stoof, C. R., De Vries, J. R., Castellnou Ribau, M., F. Fernández, M., Flores, D., Galarza Villamar, J., ... & Fernandes, P. M. (2024). Megafire: An ambiguous and emotive term best avoided by science. Global Ecology and Biogeography, 33(2), 341-351.
Tangney, R., Paroissien, R., Le Breton, T.D. et al. Success of post-fire plant recovery strategies varies with shifting fire seasonality. Commun Earth Environ 3, 126 (2022). https://doi.org/10.1038/s43247-022-00453-2
Tedim, F., Leone, V., Amraoui, M., Bouillon, C., Coughlan, M. R., Delogu, G. M., ... & Xanthopoulos, G. (2018). Defining extreme wildfire events: Difficulties, challenges, and impacts. Fire, 1(1), 9.

Turco, M., Jerez, S., Augusto, S. et al. Climate drivers of the 2017 devastating fires in Portugal. Sci Rep 9, 13886 (2019). https://doi.org/10.1038/s41598-019-50281-2
Vilà-Vilardell, L., De Cáceres, M., Piqué, M., & Casals, P. (2023). Prescribed fire after thinning increased resistance of sub-Mediterranean pine forests to drought events and wildfires. Forest Ecology and Management, 527, 120602.
Zuur, A. F., Ieno, E. N., Walker, N. J., Saveliev, A. A., & Smith, G. M. (2009). Mixed effects models and extensions in ecology with R (Vol. 574, p. 574). New York: Springer.
Zuur, A. F., Ieno, E. N., & Elphick, C. S. (2010). A protocol for data exploration to avoid common statistical problems. Methods in ecology and evolution, 1(1), 3-14.

(Remarks on code availability)
The rgdal package has not been available since 2023 (https://r-spatial.org/r/2022/04/12/evolution.html)
The R code provided should be revised. I got several errors.

Version 1:

Reviewer comments:

Reviewer #1

(Remarks to the Author)
The authors have done an excellent work addressing the concerns raised in the previous round. The revisions substantially improve the manuscript, and the responses are thorough and convincing. In particular, I commend the careful re-evaluation of the EFFIS database, detecting the forest cover estimation inconsistences, This significantly strengthens the robustness and reliability of the analysis, and also confirms that this peak is not due to an artifact caused by the sensor change. The additional methodological clarifications, the sensitivity analysis of the Canadian Fire Weather Index, and the expanded discussion of human influence are all well integrated and enhance the overall quality of the work.

One remaining point deserves further consideration. While the implementation of a DEM-based first-order lapse-rate correction for temperature is appropriate and improves the representation of thermal gradients, limiting the downscaling procedure to temperature alone may introduce inconsistencies in mountainous environments. Other key climatic variables relevant to fire dynamics —such as precipitation, VPD, PET — also exhibit strong spatial heterogeneity driven by microtopography. In particular, precipitation gradients can critically influence fuel moisture and, consequently, fire occurrence and behavior. Moreover, the use of mixed spatial resolutions across predictors (i.e., downscaled temperature versus coarser-resolution moisture-related variables) may introduce internal inconsistencies in the analysis, potentially affecting model sensitivity and the relative importance attributed to different drivers. In this context, it would be preferable to apply a consistent downscaling strategy across all key climatic variables, or alternatively to retain all variables at their native resolution. A brief discussion acknowledging these limitations and justifying the adopted approach would further strengthen the methodological transparency of the study.

Finally, I find the composite quinquennial centroids figure to be excellent, as it greatly enhances the clarity of the spatiotemporal patterns described in the manuscript.

(Remarks on code availability)

Reviewer #2

(Remarks to the Author)
My earlier comments and concerns have been satisfactorily addressed in this new version of the manuscript, for which I congratulate the authors.

(Remarks on code availability)

Version 2:

Reviewer comments:

Reviewer #1

(Remarks to the Author)
The authors have excellently addressed all the issues raised, and I congratulate them on the final version of Figure 1.

(Remarks on code availability)

Open Access This Peer Review File is licensed under a Creative Commons Attribution 4.0 International License, which permits use, sharing, adaptation, distribution and reproduction in any medium or format, as long as you give appropriate credit to the original author(s) and the source, provide a link to the Creative Commons license, and indicate if changes were made.
In cases where reviewers are anonymous, credit should be given to 'Anonymous Referee' and the source.
The images or other third party material in this Peer Review File are included in the article's Creative Commons license, unless indicated otherwise in a credit line to the material. If material is not included in the article's Creative Commons license and your intended use is not permitted by statutory regulation or exceeds the permitted use, you will need to obtain permission directly from the copyright holder.
To view a copy of this license, visit https://creativecommons.org/licenses/by/4.0/

Response to Reviewer 1

We thank Reviewer 1 for the constructive and thoughtful evaluation of our manuscript. In response to these suggestions, we strengthened the methodological framework, clarified analytical choices, and expanded several aspects of the analysis. Specifically, we:

1. evaluated the Canadian Fire Weather Index (FWI) and added a sensitivity analysis confirming the dominant role of atmospheric dryness;
2. clarified the resolution of climate data and implemented a DEM-based lapse-rate correction to better represent topographic gradients;
3. expanded the description and interpretation of human influence, including the components and range of the Human Footprint Index;
4. provided a stronger justification of the 1,400 m elevation threshold through statistical breakpoint analyses;
5. re-examined the EFFIS database, incorporated the most recent fire records (extending the dataset from 2000–2022 to 2000–2025), and corrected inconsistencies in forest-cover attribution identified in an earlier dataset release.

All analyses and figures were updated accordingly.

A detailed point-by-point response to all reviewer comments is provided below. Reviewer comments are shown in black, our responses in blue, and changes in the revised manuscript are indicated in red.

We thank the reviewer for the constructive comments, which helped improve the manuscript.

Thank you for helping us to improve the manuscript.

Yours sincerely

Mirela Beloiu and Fanny Petibon

On behalf of the authors

1 # Reviewer 1

Reviewer #1 (Remarks to the Author): This manuscript addresses a highly relevant and timely question: whether climate change is enabling an upslope expansion of forest fires across European mountain regions. Using EFFIS fire records, climatic time series, and mixed statistical modelling, the authors demonstrate a clear post-2015 increase in high-elevation fire occurrence, with atmospheric aridity (notably VPD) emerging as the dominant driver of burned area.

The study is ambitious, methodologically solid, and contributes valuable continental-scale evidence. However, several aspects require clarification and methodological refinement before the manuscript can be considered for publication. In particular, (i) the analysis would benefit from integrating a composite fire weather index such as FWI, (ii) high-resolution climatic data should be considered for mountainous terrain, and (iii) the representation of anthropogenic drivers is too limited.

Thank you for your suggestions that helped us to improve the storyline of the manuscript. We have revised the manuscript and you will find our responses to each of your suggestions below.

1.1 Major comments:

Integration of fire weather indices (FWI)

The study convincingly identifies VPD as the dominant climatic predictor of burned area, but the analysis treats climatic variables independently, overlooking their non-linear and synergistic interactions. I strongly recommend testing whether the Canadian Fire Weather Index (FWI)—already operational in EFFIS and Copernicus systems—better explains burned area and elevation patterns than the current suite of single variables (VPD, PPT, SM, PET, etc.) avoiding variables collinearity. FWI integrates temperature, humidity, wind, and precipitation into a standardized measure of fire danger, enabling a consistent comparison across regions and years and aligns with established European fire risk frameworks.

We thank the reviewer for recommending the Canadian Fire Weather Index (FWI). We agree that FWI is a widely used operational fire danger metric and have therefore added an additional analysis including FWI for 2000–2025 (Table 1 and Fig. S4d).

1. Conceptual objective and mechanistic interpretation

Our objective is to identify mechanistic climatic drivers of burned area across elevation gradients. We therefore model atmospheric dryness (VPD), climatic water balance (AET, DEF), precipitation supply (PPT), soil moisture, and temperature separately to preserve process-level interpretability. Because FWI integrates temperature, humidity, wind, and precipitation into a single scalar index, including it together with its component variables introduces strong multicollinearity and reduces causal interpretability.

FWI confirms the importance of dryness-driven fire conditions but does not allow separation of the specific processes driving the observed upslope fire shift. Because our central question concerns mechanistic elevation-dependent responses, we retain individual climatic variables in the main analysis and added the analysis on FWI to Table 1. Consistent with the other climatic predictors reported in Table 1, FWI shows higher

fire weather severity above the 1,400 m elevation threshold: the median FWI across the entire dataset is 15.62, increasing to 23.02 for fires occurring above 1,400 m and decreasing to 12.33 for fires below 1,400 m.

2. Empirical relationship in our dataset

In our dataset, FWI_mean is strongly positively correlated with VPD and DEF, followed by Tmax and PET, and negatively correlated with soil moisture and precipitation (Fig. S4a). This pattern is consistent with evidence that atmospheric evaporative demand is a major large-scale driver of burned area in many fire-prone regions (Williams et al., 2019; Alizadeh et al., 2021)**. Thus, the FWI sensitivity analysis confirms rather than alters our main conclusions.**

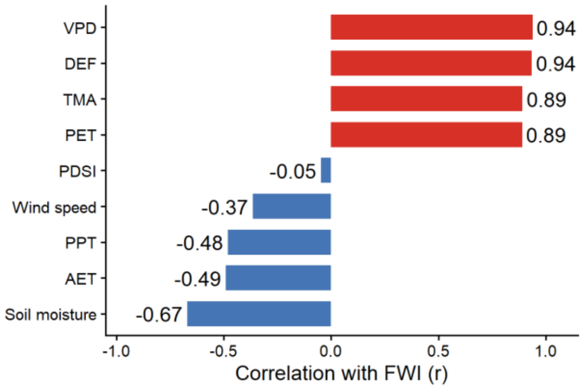

Fig. S4d. Correlation of the Canadian Fire Weather Index (FWI) with our mean variables (2000 to 2024), showing strong positive associations with VPD and moisture deficit and negative associations with precipitation and soil moisture.

3. Performance in mountain environments

FWI was originally developed for boreal fuel systems (Van Wagner, 1987) **and can perform variably in complex mountain terrain characterized by steep elevation gradients, heterogeneous microclimates, and snow-influenced fuel regimes. For instance, the FWI dataset has a spatial resolution of 0.1° (~8–10 km in Europe), which is substantially coarser than TerraClimate (~4 km). Such grid sizes can smooth sharp elevational and microclimatic gradients typical of mountain systems, potentially reducing sensitivity to fine-scale elevation-dependent fire–climate interactions. By contrast, vapor pressure deficit (VPD) has been identified as a robust large-scale predictor of burned area and it emerged as the strongest predictor in our models.**

4. Revision to the manuscript:
We have added the following text to the Methods L361-363:
"*The Canadian Fire Weather Index (FWI) 80 (unitless, range 0 to >50, low to extreme) was evaluated but excluded from the driver analysis due to strong collinearity with several climatic predictors (e.g., VPD, DEF, PET) (Fig. S4d).* "
Results: Table 1

"Fires occurring below 1,400 m were associated with higher water availability, reflected in greater actual evapotranspiration (AET), precipitation (PPT), soil moisture, wildfire intensity and danger (FWI)."
Supplementary materials: ***Fig. S4d Correlation of the Canadian Fire Weather Index (FWI) with mean climatic variables (2000 to 2024).***

Climate data resolution and anthropogenic variables

The Discussion should more explicitly address the influence of data resolution and the limited representation of anthropogenic factors. The use of TerraClimate at 4 km resolution likely obscures the strong topoclimatic variability typical of mountainous regions, where temperature, humidity, and wind change sharply over short distances. I suggest clarifying this limitation and, where possible, incorporating or testing higher-resolution climatic datasets (e.g., easyclimate at 1 km² - https://doi.org/10.1016/j.envsoft.2023.105627) or applying a simple downscaling approach (lapse-rate correction, topographic bias adjustment) to better capture fine-scale climate–fire interactions. This would improve the accuracy of elevational analyses and strengthen the interpretation of climate–fire linkages.

We agree that the ~4 km resolution of TerraClimate may smooth fine-scale topoclimatic variability typical of mountain regions. TerraClimate was retained because it provides a complete and internally consistent set of drought and water-balance variables essential for our analysis, including AET, PET, soil moisture, PDSI, and climatic water deficit. These variables are not available in easyclimate (Cruz-Alonso et al., 2023), which primarily provides high-resolution temperature and precipitation fields. Because vegetation water stress and atmospheric demand emerged as dominant fire drivers in our study, excluding these variables would substantially weaken the mechanistic interpretation of climate–fire relationships.

To address the coarse resolution of TerraClimate, we applied a first-order DEM-based lapse-rate correction to temperature. First, TerraClimate monthly temperature (tmax and tmin; native resolution about 4 km) was bilinearly resampled to a 1 km grid. We then corrected temperature for sub grid topographic variation using a standard environmental lapse rate of −6.5 °C km⁻¹ (−0.0065 °C m⁻¹). The correction was based on the elevation anomaly of each 1 km cell relative to the mean elevation of its surrounding 4 km neighborhood, computed from the DEM:

$$\Delta z = z_{1km} - z_{4km}, T_{corrected} = T_{1km} + \gamma \Delta z$$

where z_{1km} is the 1 km DEM elevation, z_{4km} is the 4 km mean elevation (aggregated from the DEM and resampled back to 1 km), and $\gamma = -0.0065$°C m^{-1} (Barry & Chorley, 2009). This adjustment decreases temperatures at pixels higher than their surrounding 4 km mean elevation and increases temperatures at lower pixels, thereby better representing topographic temperature gradients relevant for elevational fire analyses. This is a first order correction and does not model local inversions or radiation related microclimates.

Revision: We now updated the methods, discussed this limitation and highlighted the value of future high-resolution climate products for regional-scale model refinement in the revised manuscript.

Revision:

Methods L352-353: "*Temperature (tmin and tmax) was additionally adjusted using a DEM-based lapse rate correction (−6.5 °C km^{-1})* (Barry & Chorley, 2009) *to account for sub-grid elevational variability in mountainous terrain*."

Discussion L271-272: "*Similarly, high-resolution climate products, such as easyclimate (Cruz-Alonso et al., 2023), would further improve the detection of fine-scale climate–fire interactions in complex mountain terrain.* ".

In parallel, the manuscript's treatment of human influence is overly simplified. The "Human Footprint Index" provides only a coarse, static proxy that underestimates key drivers such as ignition pressure, land-use change, and accessibility. The authors should consider including additional variables (road density, wildland–urban interface, agricultural abandonment, tourism intensity) or, at minimum, explicitly acknowledge this limitation to balance the current climate-dominant narrative.

Human pressure was represented using the Human Footprint Index (HFP), which is the only globally harmonized dataset providing consistent spatial coverage across all eight mountain regions over the full study period. The Human Footprint (HFP) dataset follows the global annual Human Footprint product of Mu et al. (2022) **which integrates multiple anthropogenic pressure variables, including built environments, population density, nighttime lights, cropland, pasture, roads, railways, and navigable waterways, into a composite index. The dataset is provided at 1 km spatial resolution and ranges from 0 (no detectable human pressure) to 50 (maximum cumulative human pressure), with values <10 representing low human influence, 10–30 moderate disturbance (e.g., scattered agriculture), and >30 high-impact zones such as urbanized regions. We have expanded the Methods section to briefly describe the components and range of the HFP index.**

More detailed ignition-related variables such as road density, wildland–urban interface extent, tourism intensity, or agricultural abandonment are not harmonized across countries and would introduce substantial cross-country bias. Importantly, while the majority of fires in Europe are human-ignited, our study focuses on burned area and fire spread rather than ignition probability. The growth and ultimate size of large fires (>30 ha) are primarily constrained by other factors, which explains the dominant role of atmospheric aridity in our analyses. Because large fires are more sensitive to fire weather and drought conditions than to fine-scale ignition patterns, our estimates of anthropogenic influence should be interpreted as conservative rather than dismissive of human drivers. We now explicitly acknowledge this distinction in the Discussion section.

Revision:

Methods L353-360: "*Human footprint integrates multiple anthropogenic pressure variables, including built environments, population density, nighttime lights, cropland, pasture, roads, railways, and navigable waterways, into a composite index ranging from 0 (no detectable*

human pressure) to 50 (maximum cumulative human pressure), with values <10 representing low human influence, 10–30 moderate disturbance (e.g., scattered agriculture), and >30 high-impact zones such as urbanized regions. Annual maps from 2000 to 2018 at 1 km spatial resolution were obtained from Mu et al. **(2022).*"***

Discussion L220-223: "*Although most fires in Europe are human-ignited, our results showed that the spread and final size of large fires are primarily controlled by climatic conditions and fuel dryness. Our estimates of anthropogenic influence are likely conservative, as the Human Footprint Index is a coarse proxy and does not capture fine-scale ignition and accessibility gradients.*"

Justification of the 1,400 m elevation threshold. The 1,400 m threshold is statistically derived but lacks ecological or climatic justification. Is this value consistent across mountain systems, or does it reflect distinct bioclimatic zones? A sensitivity analysis (e.g., thresholds between 1,200 – 1,600 m) or modelling elevation as a continuous effect could bring more robustness.

We thank the reviewer; we have conducted the suggested analysis. The 1,400 m threshold was informed by the elevation breakpoint analysis which we have included now in the Table S7. Across all variables, the average European cutpoint was 1,458 m and the median 1,568 m, with regime-specific medians ranging from 1,069 m (high and persistent) to 1,618 m (moderate and reoccurring). In low and emerging regimes, the median breakpoint was 1,380 m. Thus, 1,400 m represents a central tendency across multiple significant climatic and vegetation thresholds and falls within one standard deviation of the overall breakpoint distribution. We have clarified in the Methods that this threshold reflects the approximate elevation at which multiple fire–environment relationships shift, rather than an arbitrary division.

Importantly, this elevation also corresponds to a clear shift in fire distribution and vegetation composition. As shown in Tables S5a-b, most fires occur below 1,400 m (≈75%), whereas fires above this elevation are less frequent but occur under markedly drier climatic conditions. These patterns indicate that around 1,400 m multiple fire–environment relationships shift simultaneously, making it a meaningful continental-scale breakpoint. We acknowledge that the exact ecological transition varies among mountain systems, as it was shown also in tree height (Gelabert et al., 2024)**, which decreases above a climate related elevation threshold around 1,400-1,500 m in the Alps, Carpathians, and Pyrenees. Therefore, the 1,400 m threshold should be interpreted as an emergent continental-scale breakpoint rather than a fixed ecological boundary. We have clarified this point in the Methods.**

Revision:

Table S7 *Elevational breakpoints of climatic, vegetation, and human variables across European fire regimes.*

Table 5a-b, *Vegetation composition across fire region types in European mountains*

Fig. S3. *Burned area distributions and vegetation composition across region types*

Methods L405-411: "*The 1,400 m threshold reflects the central tendency of statistically derived elevation breakpoints across climatic, fuel type, and human variables (Table S7),*

which cluster around 1,400–1,600 m at continental scale. Vegetation composition within burned areas was quantified as the percentage cover of broadleaf, conifer, mixed, and transitional vegetation intersecting each fire polygon. Burned area and median vegetation cover were summarized by elevation class (≤1,400 m and >1,400 m) and fire regime type (Table S5a), and by mountain region and elevation class (Table S5b)."

Temporal discontinuity post-2015

The sharp post-2015 increase in fire activity is compelling, but it may partly reflect methodological changes in the EFFIS data stream rather than a purely climatic signal. In 2018, EFFIS transitioned from MODIS (250 m) to Sentinel-2 (20 m) imagery for burned area mapping, which markedly improved spatial resolution and enabled the detection of fires smaller than 30 ha—precisely those excluded under the ≥ 30 ha threshold applied in this study. With this technological shift, the effective minimum mapping unit changed, potentially biasing total burned area estimates and temporal trend analyses.

Therefore, the authors should verify whether the apparent post-2015 sharp increase in burned area and fire frequency correlates with the sensor transition. If possible, they should provide evidence that the observed peak is not an artifact of improved detectability or processing differences between MODIS and Sentinel-2 datasets.

We thank the reviewer for raising this point. Following this comment, we re-examined the EFFIS database, downloaded the updated dataset (2000–2025), identified inconsistencies in the earlier version (2000–2022), and rebuilt the database accordingly.

1. Dataset verification and update

During revision, we conducted an additional quality check of the EFFIS database and obtained an updated dataset extending through 2025. This revised version harmonizes reporting inconsistencies related to forest area accounting changes introduced in 2019 and 2022. Because our analysis includes only fires with ≥50% forest cover, these inconsistencies inflated the number of polygons retained in those years. For example, after applying our filtering criteria (≥30 ha burned area, ≥800 m maximum elevation - to capture upward fire spread into mountain ecosystems, ≥50% forest cover) we got the following numbers of fire polygons:

- **2019: 949 fires in the earlier dataset vs 447 fires in the updated dataset**
- **2022: 767 fires in the earlier dataset vs 441 fires in the updated dataset**

The discrepancy resulted from incorrect forest cover percentages in our earlier EFFIS dataset downloaded in spring 2023. After verifying the values with EFFIS and CORINE Land Cover 2018, we corrected and rebuilt the database using the updated version. Forest cover was defined as the sum of conifer, broadleaf, mixed forest, and transitional woodland–shrub classes within burned polygons. Because the forest cover percentages for 2019 and 2022 were lower in the updated dataset, fewer polygons met the ≥50% forest cover threshold and were therefore retained in the analysis.

Importantly, incorporating the extended time series does not weaken the post-2015 elevational trends reported in the manuscript (Fig. 2b). On the contrary, the inclusion of the most recent years reinforces the upward trajectory in burned area and fire frequency,

confirming that the observed shift represents a persistent and continuing pattern rather than a short-term anomaly or artefact.

As in the previous version, some fires between 2000 and 2005 lacked precise ignition dates and were default-recorded as 1 January. Because this does not affect annual burned area totals or elevational patterns, these fires were retained for temporal pattern analyses (and are visible in Fig. 2b). However, they were excluded from the climate-driver models to avoid mismatches with monthly climate variables.

2. Influence of the MODIS–Sentinel sensor transition

We also examined whether the post-2015 increase in fire activity could reflect changes in satellite sensors rather than real fire dynamics. Sentinel-2 imagery was integrated into the Rapid Damage Assessment (RDA) module of EFFIS system in 2019, whereas the increase in fire occurrence and burned area in our dataset begins earlier, in 2017 and 2021 (Fig. 2b). Moreover, our analysis includes fires ≥30 ha, which were already reliably detected by MODIS (250 m resolution). Sentinel-2 mainly improves the detection of smaller fires (<30 ha), which are excluded from our dataset.

Revision:

The entire manuscript and supplementary materials were updated with the new dataset values. Specifically, Tables S1ab show the distribution of burned area and number of fires across European mountain regions from 2000 to 2025.

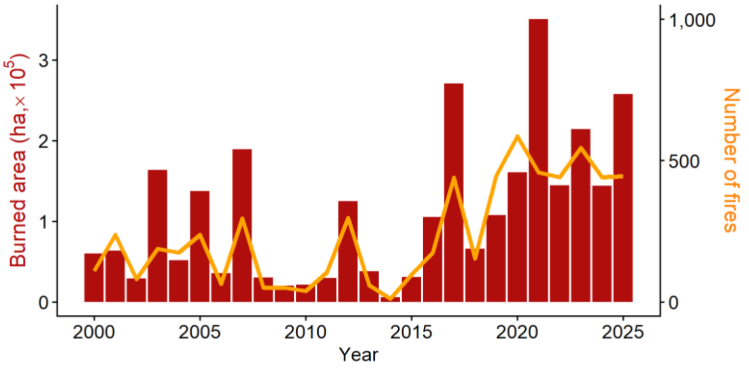

Fig. 2b. Evolution of burned area and fire number within mountain regions of Europe.

1.2 Other minor comments:

1. In the abstract, the authors could quantify the rate of upslope shift (e.g., "median elevation increased by X m per decade") to convey magnitude.

Thank you for this valuable suggestion. We agree that quantifying the rate of elevational change strengthens the clarity of the abstract. Since fire frequency remains substantially higher at lower elevations (Table S2a), changes in mean elevation are strongly influenced by the large number of low-elevation fires and therefore do not adequately represent the upward displacement of the upper fire distribution. Instead, we quantified elevational change using two-period medians and metrics derived from maximum fire elevation (Z_max), which directly capture the upper elevational reach of fires. Specifically, we calculated yearly Z_max-based metrics and compared their medians between an early

period (2000–2014) and a late period (2015–2025). The results are summarized in Table S3a.

For example:

- **D800 (yearly median Z_max of all fires ≥ 800 m) increased from 1,087 m to 1,168 m, corresponding to a shift of 62.69 m per decade (6.27 m yr^{-1}).**
- **Z90 (yearly 90th percentile of Z_max) increased from 1,601 m to 1,703 m, corresponding to 78.83 m per decade (7.88 m yr^{-1}).**
- **T10 (yearly median Z_max of the ten highest-elevation fires per year) increased from 1,725 m to 2,066 m, corresponding to 262.24 m per decade (26.22 m yr^{-1}).**

These consistent increases across Z_max-derived metrics demonstrate a pronounced upslope shift, particularly in the upper and extreme fire elevations.

In addition to the two-period median comparison reported in the revised manuscript, we conducted a complementary linear regression analysis using yearly elevation metrics (D800, Z90, and T10) across the full 2000–2025 period. The regression slopes indicate positive elevational trends for all three metrics (Table S3b). However, none of these full-period linear trends are statistically significant ($p > 0.05$), and confidence intervals include zero. This confirms that the elevational shift is not a gradual monotonic increase across the entire period but rather reflects a temporal discontinuity emerging after 2015.

We therefore retain the two-period median comparison in the main manuscript, as it better captures the regime shift nature of the post-2015 upslope expansion. The full linear trend results are now provided in the Supplementary Material (Table S3b) for completeness and transparency.

Revision:

We have now added a quantified rate of elevational increase to the abstract and Supplementary materials as well as to the results to better convey the magnitude of the observed shift.

Abstract: "*Upper fire elevations increased by ~80 m per decade after 2015.*"

Methods L328-333:

"To quantify elevational change, we used two complementary approaches. We first compared two-period medians (2000–2014 vs 2015–2025) and calculated rates using period midpoints (Table S3a). We then fitted linear regressions of yearly elevation metrics (D800, Z90, T10) against Year over 2000–2025 to test for continuous trends (Table S3b). The median approach captures the post-2015 shift, whereas the regression assesses monotonic long-term change."

Results L105-111:

"Comparisons of early (2000–2014) and late (2015–2025) periods show increases in fire elevation across all datasets: the full D800 record (median rose from 1,087 m to 1,168 m, 62.69 m decade^{-1}), the 90th percentile of annual fire elevations (Z90; 1,601 m to 1,703 m, 78.83 m decade^{-1}), and the ten highest-elevation fires per year (T10; 1,725.25 m to 2,066.16 m, 262.24 m decade^{-1}, Table S3a). Linear regressions over 2000–2025 show positive but non-

significant full-period trends (Table S3b), supporting a post-2015 regime shift rather than a gradual increase."

Supplementary materials: ***Tables S3ab Upslope shift in European mountain fire elevation before and after 2015.***

2. In Figure 1, I recommend improving the visual interpretability of the map by converting the burned-area polygons into point representations (e.g., centroids), to reduce overlap. Given that European mountain ranges are already well known, the authors could simply shade the mountainous areas in obscure-grey to provide spatial context without distracting from the main signal. Additionally, colour gradients could then be used to represent quinquennial periods of fire occurrence, effectively conveying both spatial and temporal dynamics of fire distribution across the continent.

Thank you for this constructive suggestion. It helped us improve the visual clarity and temporal interpretability of the spatial patterns. Following the reviewer's recommendation, we tested several alternative cartographic representations. Specifically:

1. **Converted burned-area polygons to centroid points to reduce spatial overlap.**
2. **Encoded time using quinquennial bins**
3. **Tested a simplified split (before vs after 2015) (see below figures a-e).**
4. **Produced both combined and separate temporal panels to assess readability at continental scale.**

The centroid-based quinquennial map (Fig. d below) is now included in the Supplementary Material as Fig S1, while Fig. f is retained in the main manuscript as Fig 1. We decided to keep the mountain regions since they are informative for readers unfamiliar with Europe, and we added the quinquennial periods which offer illustrate the spatio-temporal changes. The other versions from below, a, b, c, e were discarded since they did not seem to offer enhanced information. However, if any of the maps below is preferred by the reviewer, we are happy to introduce them in the manuscript.

Revision: Fig. S1, Fig 1.

Tests considering different groupings of forest fires:

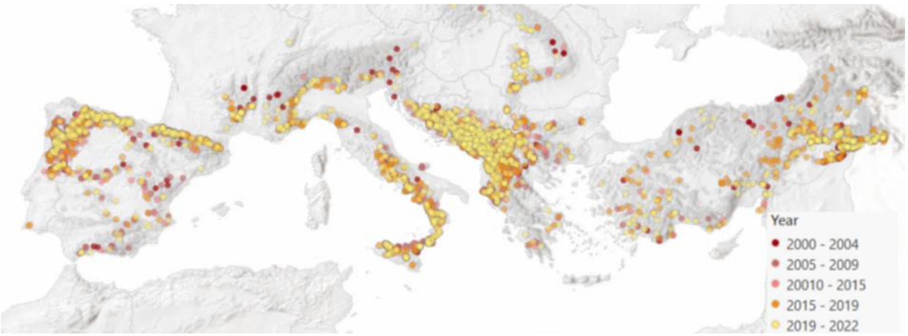

a. Burned areas shown as centroids aggregated into five quinquennial periods (2000–2004, 2005–2009, 2010–2014, 2015–2019, 2020–2022).

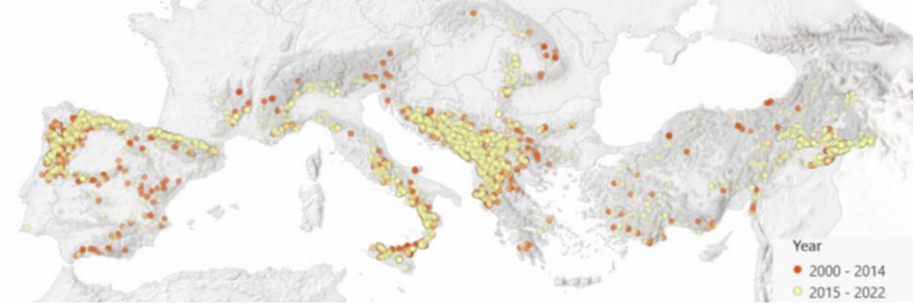

b. Centroid representation split into two periods (2000–2014 and 2015–2022).

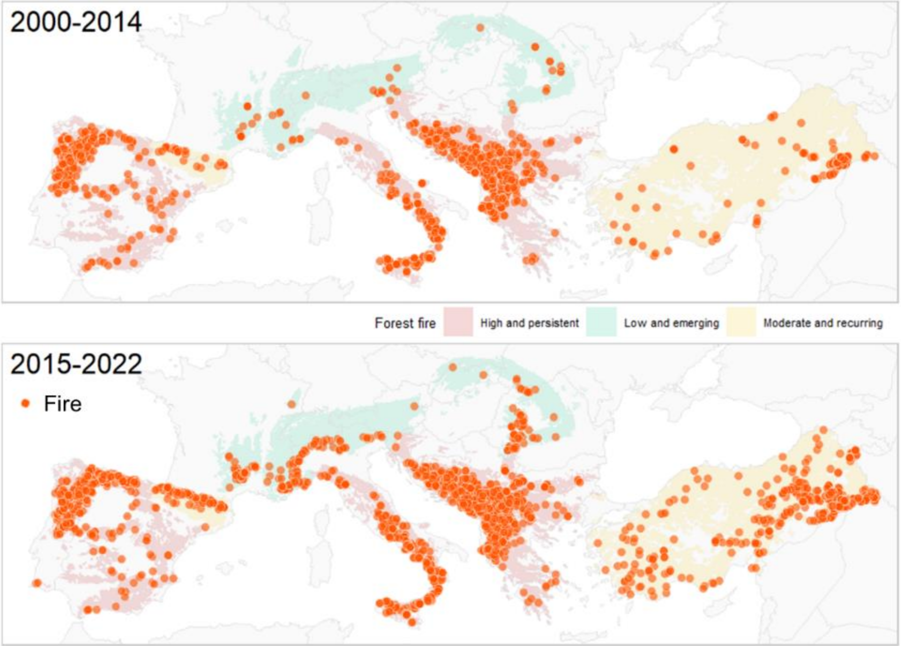

c. Burned area as a centroid for two classes based on years before and after 2014 in separate maps.

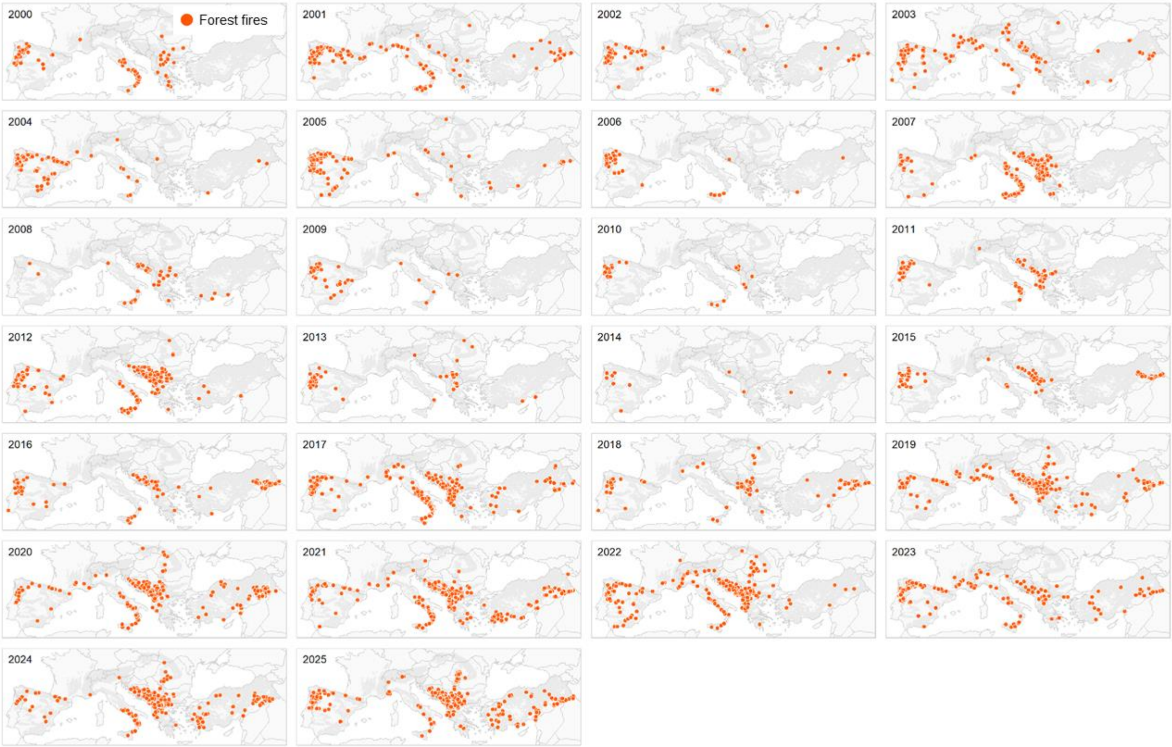

d. Spatial distribution of burned areas (2000–2025, ≥ 30 ha) across major European mountain systems. Red polygons indicate individual fire events. Shaded areas represent mountain regions analyzed in this study.

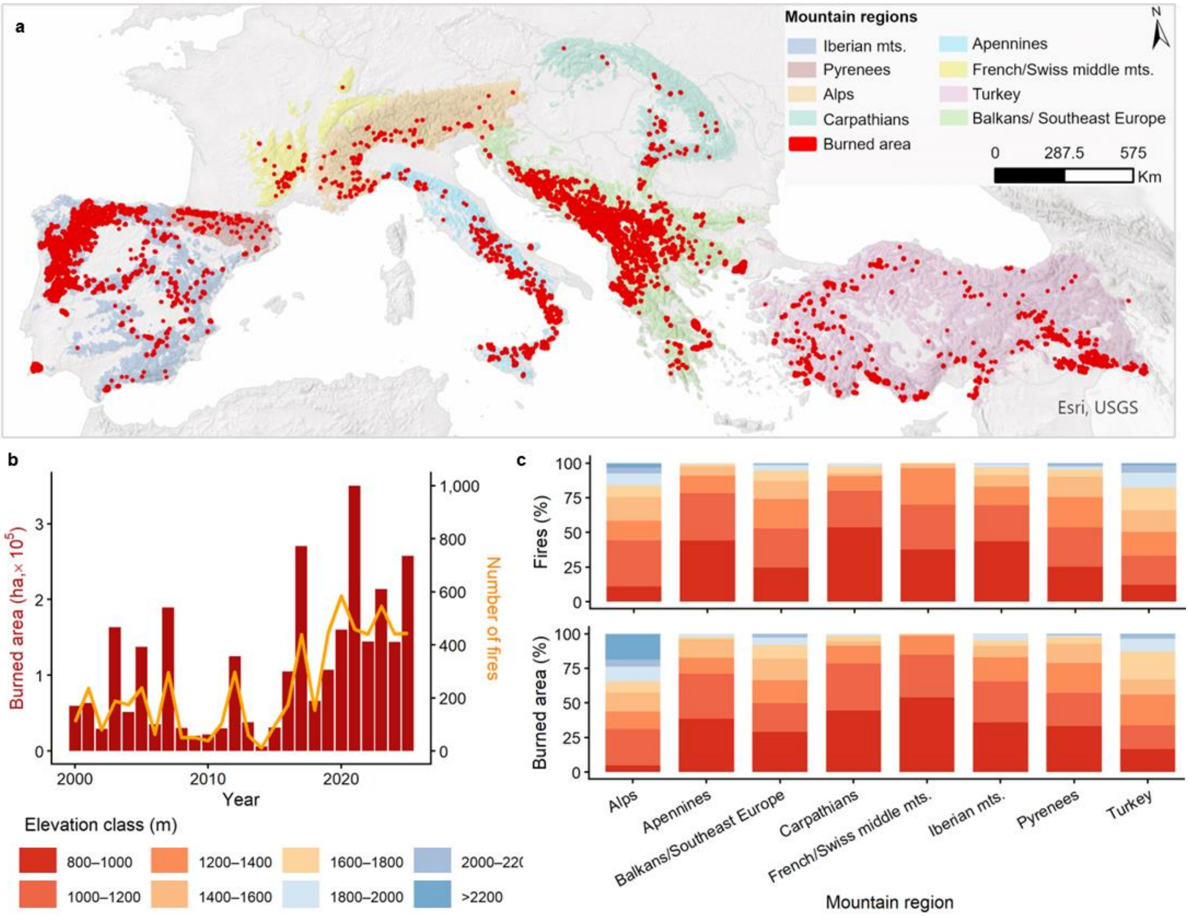

e. Spatial patterns of forest fire. a. Spatial distribution of forest fires (6,225) that occurred within the eight European mountain regions (≥800 m) from 2000 to 2025. b Evolution of burned area and fire number within mountain regions of Europe. c Percentage of fire occurrences and burned area (ha) for each mountain region and elevation class.

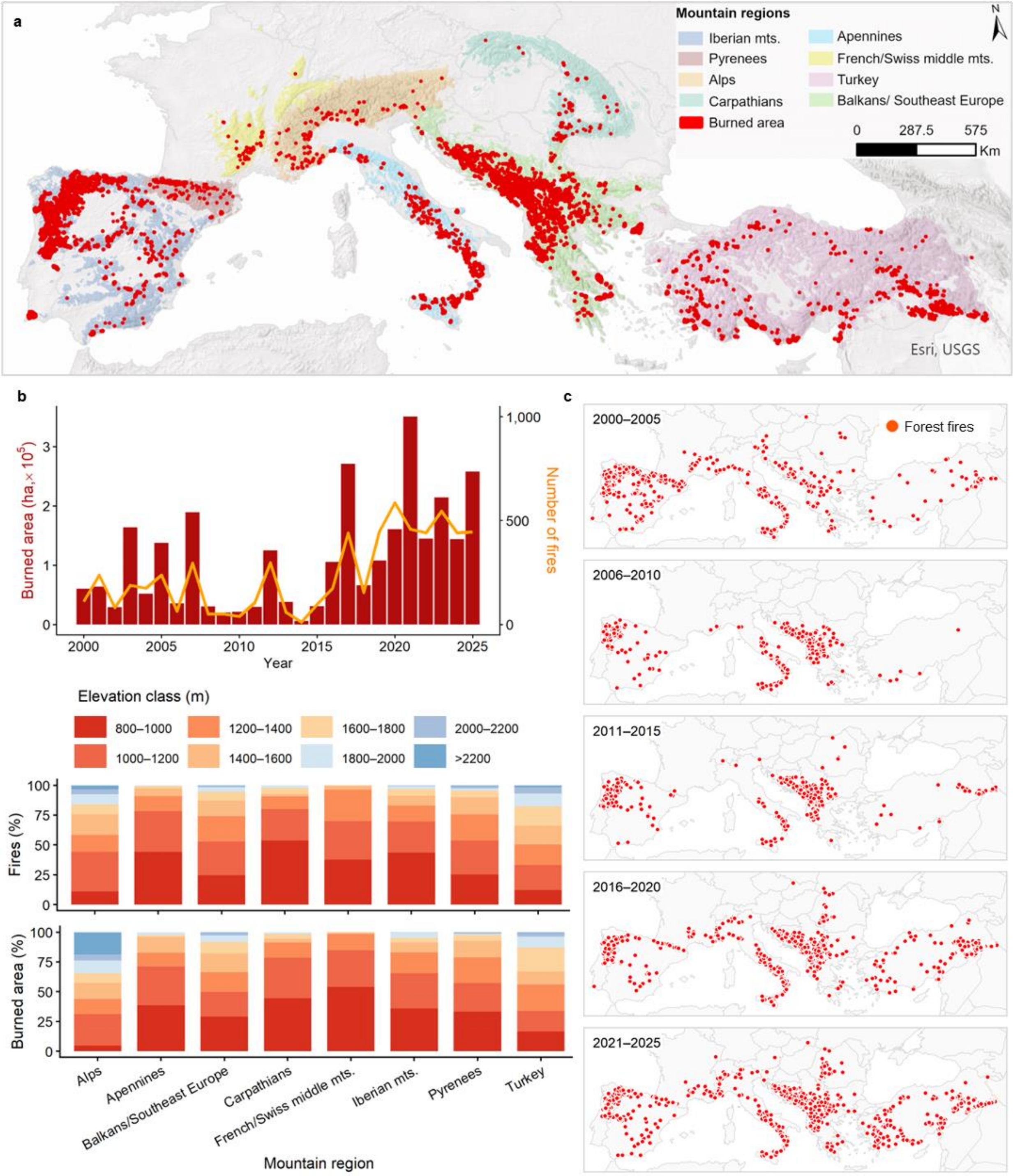

f. Spatial distribution of forest fires (6,225) that occurred within the eight European mountain regions (≥800 m) from 2000 to 2025. b Evolution of burned area and fire number within mountain regions of Europe and percentage of fire occurrences and burned area (ha) for each mountain region and elevation class. c Centroid locations of burned areas grouped into quinquennial periods. The figure illustrates the progressive spatial expansion and intensification of fire activity, particularly after 2015.

Thank you for helping us to improve the manuscript!

2 References

Alizadeh, M.R., Abatzoglou, J.T., Luce, C.H., Adamowski, J.F., Farid, A., & Sadegh, M. (2021) Warming enabled upslope advance in western US forest fires. *Proceedings of the National Academy of Sciences*, 118(22), e2009717118. https://doi.or/10.1073/pnas.2009717118

Barry, R.G., & Chorley, R.J. (2009) *Atmosphere, Weather and Climate*. Routledge: London. https://doi.or/10.4324/9780203871027

Cruz-Alonso, V., Pucher, C., Ratcliffe, S., Ruiz-Benito, P., Astigarraga, J., Neumann, M., et al. (2023) The easyclimate R package: Easy access to high-resolution daily climate data for Europe. *Environmental Modelling & Software*, 161, 105627. https://doi.or/10.1016/j.envsoft.2023.105627

Gelabert, P.J., Rodrigues, M., Coll, L., Vega-Garcia, C., & Ameztegui, A. (2024) Maximum tree height in European Mountains decreases above a climate-related elevation threshold. *Communications Earth & Environment*, 5(1), 84. https://doi.or/10.1038/s43247-024-01246-5

Mu, H., Li, X., Wen, Y., Huang, J., Du, P., Su, W., et al. (2022) A global record of annual terrestrial Human Footprint dataset from 2000 to 2018. *Scientific Data*, 9(1). https://doi.or/10.1038/s41597-022-01284-8

Van Wagner, C.E. (1987) *Development and structure of the Canadian forest fire weather index system*.

Williams, A.P., Abatzoglou, J.T., Gershunov, A., Guzman-Morales, J., Bishop, D.A., Balch, J.K., & Lettenmaier, D.P. (2019) Observed Impacts of Anthropogenic Climate Change on Wildfire in California. *Earth's Future*, 7(8). https://doi.or/10.1029/2019EF001210

Response to Reviewer 2

We thank Reviewer 2 for the constructive and thoughtful evaluation of our manuscript. In response to these suggestions, we strengthened the methodological framework, clarified analytical choices, and expanded several aspects of the analysis. Specifically, we:

1. clarified the rationale for using maximum fire elevation (Zmax) to capture upslope fire spread and provided additional statistics on mean fire elevation across mountain regions;
2. added supplementary analyses describing the distribution of minimum, mean, and maximum fire elevations and included additional tables summarizing elevation characteristics of fires across mountain regions;
3. expanded the statistical methods description, including justification of the GLMM and GAM model structures, the treatment of temporal trends, and the use of log-transformed burned area;
4. conducted a seasonal analysis of fire occurrence, including new tables and figures summarizing shifts in peak fire timing across mountain regions;
5. strengthened the discussion of fuel, vegetation, and topographic influences on burned area and clarified limitations related to fuel load and structure representation.
6. re-examined the EFFIS database, incorporated the most recent fire records (extending the dataset from 2000–2022 to 2000–2025), and corrected inconsistencies in forest-cover attribution identified in an earlier dataset release.

All analyses and figures were updated accordingly.

A detailed point-by-point response to all reviewer comments is provided below. Reviewer comments are shown in black, our responses in blue, and changes in the revised manuscript are indicated in red.

We thank the reviewer for the constructive comments, which helped improve the manuscript.

Thank you for helping us to improve the manuscript.

Yours sincerely

Mirela Beloiu and Fanny Petibon

On behalf of the authors

1 Reviewer #2 (Remarks to the Author)

The study by Beloui et al. analyses the upward shift of wildfires in mountainous areas across southern and central Europe. The research gap and objectives of the study are clearly stated and of interest, as they focus on forests that are generally not fire-prone and thus, more vulnerable to fire-related mortality and regeneration failure, putting the ecosystem services they provide at risk. The authors used remote sensing and climatic time series from 2000 to 2022 to study the upward shift of wildfires in mountains during the last two decades. They used forest fire records from the EFFIS database (burned area > 30 ha) across eight mountain regions and analysed those that burned above 800 m elevation using climate, forest type and human footprint as predictors.

Overall, the manuscript is well written and results interesting, but analyses are not always properly justified and the discussion lacks critical reflection. I believe that after revision it can be considered for publication by Nature Communications.

My main concerns relate to the methodology:

- Elevation threshold for including wildfires: The authors included in the analysis all wildfires whose maximum elevation was > 800 m, which includes a substantial number of fires that actually originate below the threshold and only burn a small portion above 800 m. This choice is not explained in the manuscript and it is not clear why the authors chose the maximum elevation instead of the mean elevation as the threshold to include fires, which seems more appropriate. A quick scan of the dataset shows that almost 25% of the fires have a mean elevation below 800 m, and these fires account for 44% of the total burned area. If the authors have valid reasons for their approach, it should be clearly stated that these fires do not occur entirely above 800 m. It would also be useful to include the mean elevation of fires in a supplementary table, broken down by mountain region, for example.

- Statistical models: The justification for the statistical models is not clearly explained. I provide more specific comments on this below.

Regarding the discussion, a deeper reflection is needed on how burned area is not only influenced by climatic conditions and the type of vegetation cover, but highly related to the amount of fuel load and the topography (e.g., slope plays a critical role and it is not addressed in the current manuscript). In the following I provide more detailed comments.

Thank you, we have followed the suggestions. We added the suggested analysis, justified statistical models and revised the discussion below, as well as in the manuscript.

Elevation threshold for including wildfires:

(1) Both approaches were tested, i.e. the mean elevation and the max elevation, and both provided similar results. Since our aim is to evaluate fire spread on high elevation, we decided to keep the max elevation, which better reflects how a fire spreads up. The rationale for selecting the maximal elevation: In region like Ticino in Switzerland fire suppression are focused on lowest elevation of the fire for protecting settlement but the upward spread is not actively suppressed. Also for our study it was important to assess the maximal elevations that were affected by fires and so by how many fire were reaching up to 800 meter defined as a threshold to determine mountain regions, implying that fire

starting at lower elevations can reach mountain ecosystems usually not fire-prone or fire-adapted.

(2) To address the reviewer's concern about the unbalanced contribution of fires that originate below the threshold and only burn a small portion above 800 m, we plotted the data in Fig S4b to better represent the data and clarified in the methods the reasons as well as tested both approaches (both thresholds yielded similar elevational trend).

Further, we calculated the mean elevation of fires and add it to a supplementary table (Table S2c), broken down by mountain region, as the reviewer suggested. We also calculated the percentage and indeed, a total of 1,532 fires (24.6%) had a mean elevation below 800 m but reached elevations above 800 m. These fires accounted for 1.49 million hectares of burned area (52.4% of total burned area), indicating that many large fires originate at lower elevations but spread upslope into mountain environments.

(3) The reviewers raised additional concerns regarding the justification of the analyses and statistical models chosen, as well as need for deeper critical reflection in the discussion regarding the contribution of fuel and topography. These aspects are addressed more specifically in the next comments.

(4) Revision to the manuscript:

Supplementary: *Table S2c. Elevation characteristics of forest fire events across European mountain regions (2000–2025).* This table shows that the average mean fire elevation (Z_mean) ranges from ~860 m (Apennines) to ~1,220 m (Turkey), while mean maximum elevations range from ~1,060 m to ~1,430 m, confirming that the majority of fires in the dataset extend well into mountain environments.

Fig. S4b: The distributions of minimum, mean, and maximum fire elevations (Fig. S4b) show that many fires span strong elevational gradients, with minimum elevations often below 800 m but maximum elevations commonly exceeding 1000 m, indicating that these events extend into mountain environments.

We added the following text to the Method L312-314:

"*We used maximum elevation to capture upward fire spread into mountain ecosystems, including fires originating below 800 m that extend into higher elevations. Sensitivity analyses using mean elevation (≥ 800 m) produced consistent results.*"

We added the following text to the Discussion: L191-199

"*Elevational analyses revealed rising fire occurrence across all ranges (800 m to > 2,000 m), with the steepest increase at mid-elevations (800–1,400 m) and detectable gains above 2,000 m. However, 1,532 fires (24.6%) originate below 800 m but spread above this threshold, accounting for 1.49 million ha of burned area (52.4%), suggesting that climatic barriers that historically limited fire spread to higher elevations are weakening under increasingly dry and fire-prone conditions and highlighting implications for fire management strategies that are typically concentrated near human settlements rather than remote mountain areas.*"

***Abstract**

I would appreciate more details of the study in the abstract. For example, type of vegetation cover and human footprint are not mentioned as part of the analysis, or number of mountain ranges evaluated.

Thank you, we added that now:

Revision abstract: L14-17

"*However, changes in the elevational distribution of forest fires in eight European mountain regions and their drivers remain largely unknown. Using satellite-derived burned area data (2000–2025), we integrated climate time series, soil moisture, forest land cover, and human footprint data in statistical models to assess fire activity.*"

L17. It depends on the region, but a 30 ha fire is typically not considered large. I would suggest to just state "forest fires > 30 ha".

We corrected that.

***Introduction**

L33. Since this paper focuses on Europe, why don't you mention Europe as an example? For example, in Portugal in 2017 there were extreme wildfires that burned large areas and even caused fatalities (Turco et al., 2019).

Thank you for the suggestion. We started with an introduction about global fire situation and added European examples in lines 40-44. We added also the fires from 2017 from Portugal.

Revision: L39-44.

"Europe has not been spared, for instance, although fire suppression reduced burned areas in the late 20th century (Turco et al., 2016; Ellis et al., 2022), recent record-breaking fire seasons, such as in 2017 (1.2 million ha, of which ~500 000 ha burned in Portugal and >120 lives were lost (Turco et al., 2019)), 2021 (500,566 ha), and 2022 (837,212 ha), have reversed this trend (San-Miguel-Ayanz et al., 2018, 2022, 2023; Mary Petritan & Beloiu Schwenke, 2023)."

L35. The concept of megafires is ambiguous, as it is related to the size of the fire. However, climate change is not only increasing fire size but also the amount of energy that fires release. A single fire may not be particularly large but release huge amounts of energy, spread erratically and cause severe damage. That's why this concept is increasingly being replaced by "extreme wildfire events" (e.g., Tedim et al., 2018; Stoof et al., 2024; Linley et al., 2022).

We thank the reviewer for this important clarification. We agree that the term "megafires" can be ambiguous because it primarily refers to fire size, whereas recent literature emphasizes that extreme wildfire behaviour is not solely defined by area burned but also by energy release, erratic spread, and socio ecological impacts. To better reflect this broader conceptual framing, we have replaced "megafires" with "extreme wildfire events", following Tedim et al. 2018, Linley et al. 2022, and Stoof et al. 2024.

Revisions: L 36-37

"These events reflect widespread shifts in fire regimes, with longer fire seasons 14, expanding burned area 15, and more frequent extreme wildfire events marked by elevated fire intensity and severe impacts 13,16–18."

L38. Fire suppression may have reduced burned areas in the late 20th century, but it has also contributed to increase fuel load, increasing the likelihood of larger and more intense wildfires, the so-called fire paradox (e.g., Kreider et al., 2024). This aspect should be acknowledged in the sentence.

We thank the reviewer for this important point. We have revised the sentence to acknowledge the fire suppression paradox and its role in fuel accumulation and increased fire intensity (Kreider et al., 2024)**.**

We have clarified that the decline in burned area during the late 20th century has been attributed to increased fire management efforts following the large fire episodes of the 1980s, including enhanced suppression capacity, expanded prevention measures, and institutional and technological improvements (Turco et al., 2016)**. We also acknowledge that prolonged fire exclusion may have increased fuel loads and connectivity, which might limit long term effectiveness** (Turco et al., 2016; Kreider et al., 2024)**.**

Revision : L39-44

"*Europe has not been spared, for instance, improved fire management, including suppression and prevention policies, reduced burned areas in the late 20th century* (Turco et al., 2016; Ellis et al., 2022)***, although prolonged fire exclusion may have increased fuel loads and connectivity, potentially limiting long term effectiveness*** (Kreider et al., 2024)**.**

L42. "central and northern Europe will be affected due to high fuel availability, warming, and drought" – this sentence is not entirely correct, since fuel availability depends largely on temperature and relative humidity. It would be more precise to state "due to high fuel load, which becomes increasingly available under warm and dry conditions" or "due to high fuel availability as a consequence of increasing temperatures and drought conditions", or similar.

Yes, we agree, thanks for the constructive suggestion.

Revision: L45-46

"*While fires continue to be most frequent in the Mediterranean, projections suggest that central and northern Europe will be increasingly affected due to high fuel loads that become more available under warming and drought conditions*."

L42. "Yet, it remains unclear whether current fire activity already signals a sustained upslope expansion into less-adapted forests" – that's not entirely accurate. There is evidence suggesting these trends. The topic may be understudied, but several studies have already analysed these patterns, and some of them are cited in your manuscript.

We acknowledge that elevational shifts in fire activity have been documented, e.g. in North America and in a local European study, while much of the European evidence remains projection based. Our intention was not to suggest a lack of evidence, but to emphasize that a consistent observational pattern of sustained upslope fire expansion across multiple European mountain systems has not yet been firmly established. We have revised the text accordingly to clarify this distinction and to stress that our point concerns continental scale consistency.

Revision: L46-*50*

"Although elevational increases in fire activity have been reported in some regions, it remains unclear whether current fire activity already signals a sustained upslope expansion into less-adapted European mountain systems, where even modest increases in fire activity could trigger disproportionate ecological and societal impacts."

L46. "Moisture content" instead of "moisture availability".

Corrected.

L51. Interplay between ignitions, fuel, climate, AND TOPOGRAPHY.

Thank you, added.

L56. One of the cited studies in this sentence (Dupire et al., 2017) refers to the French Alps, not to southern Europe.

Indeed, we rephrased it.

Revision: L62

L61. I suggest avoiding the use of the adjective large. Moreover, most mountain fires burn less than 30 ha. It is OK to limit the analyses to fires > 30 ha because this is the threshold in EFFIS database, but it should be acknowledged that this approach excludes most of the fires that burn in mountain areas (for example, in the Alps most wildfires are < 10 ha – Conedera et al., 2018). An increase in fire activity of fires < 30 ha is also likely and probably already happening – this limitation should be also acknowledged in the manuscript.

We acknowledge that most mountain fires, particularly in the Alps, are smaller than 30 ha (e.g., Conedera et al., 2018), and that national inventories, including Switzerland, record a fires below this threshold. For this reason, fires exceeding 30 ha represent comparatively large events within the Alpine context, as they exceed the size of most recorded mountain fires. While we removed the adjective "large" from the abstract, but re retained the term "large" in the introduction for > 30 ha fires in the European mountain context, while explicitly acknowledging that this threshold excludes many small fires and may not capture trends in fires < 30 ha. This limitation has been clarified in the manuscript in the discussion section.

Revision - Discussion: L266-272

"Our findings also point to several future research needs. Higher-resolution fire data (e.g., Sentinel-2), and harmonized fine-scale ignition metrics, and local incident reports should be integrated to better capture recent, small, and emerging fires at high elevations. Between 1980 and 2010, the vast majority of Alpine wildfires were smaller than 10 ha (Conedera et al., 2018)***, indicating that shifts in fire activity at higher elevations may be underestimated in our analysis, which considers only fires > 30 ha."***

*****Results***

L80. The caption of Figure 1 should be clarified. As it stands, it gives the impression that the mountain regions shown on the map correspond to areas above 800 m, but the authors actually mean that the figure displays the fire polygons that burned above 800 m within the mountain regions defined by Kapos et al. (2000). Moreover, in Figure 1b, it is somewhat confusing that the number of fires is below 1,000 while the y-axis extends up to 3,000. It would be clearer if the axis range matched the data range.

We modified the figure caption and the figure 1b to reflect better the number of fires on the x axis.

Revision: Fig. 1b and figure caption.

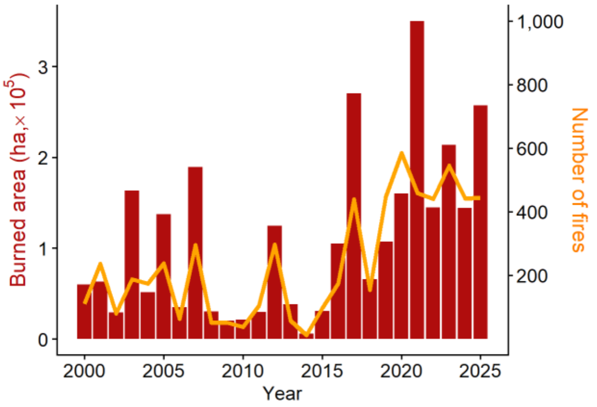

Fig. 1. a Spatial distribution of forest fires (6,225) that occurred above 800 m elevation within the eight European mountain regions as defined by Kapos et al. (2000) ***from 2000 to 2025. b Evolution of burned area and fire number within mountain regions of Europe.***

L93. Table S2 does not show a trend in fire occurrence. It shows the total number of fires by elevation class and mountain region.

Corrected.

L96. Figure 2b. The distribution of fires in the D800 dataset seems quite similar. How do you explain the significant p-value?

The significant p-value reflects the large sample size in the D800 dataset (n = 1,998 fires before 2015 and n = 4,227 after 2015), which increases statistical power and allows the detection of small distributional differences. Although the elevation distributions visually overlap, the Kolmogorov–Smirnov test detects subtle but consistent shifts between the two

periods. With large samples, the KS test becomes highly sensitive and may yield very small p-values even when differences appear modest.

To better quantify the magnitude of change, this time using the maximum elevation (Z_max), we therefore complemented this test with an analysis of median elevation shifts before and after 2015 using the three datasets (D800, Z90, T10), which provides a robust estimate of the upslope shift (Table S3ab). Z_max captures the highest elevation reached by a fire and was therefore used in the analysis, whereas Z_mean represents the center of the burned polygon and dampens extreme values. Because our objective is to quantify the upper elevational reach of fires, upslope shifts were derived from maximum fire elevation (Z_max), which retains fires that originate below 800 m but spread upslope into mountain ecosystems.

Revision: ***Tables S3ab Upslope shift in European mountain fire elevation before and after 2015.***

Abstract: L19 "Upper fire elevations increased by ~80 m per decade after 2015."

Results: L103-111

"Elevation density distributions based on mean elevation show a clear upward displacement after 2015 across the full fire footprint (Fig. 2b). Comparisons of early (2000–2014) and late (2015–2025) periods show increases in fire elevation across all datasets: the full D800 record (median rose from 1,087 m to 1,168 m, 62.69 m decade^{-1}), the 90th percentile of annual fire elevations (Z90; 1,601 m to 1,703 m, 78.83 m decade^{-1}), and the ten highest-elevation fires per year (T10; 1,725.25 m to 2,066.16 m, 262.24 m decade^{-1}, Table S3a). Linear regressions over 2000–2025 show positive but non-significant full-period trends (Table S3b), supporting a post-2015 regime shift rather than a gradual increase."

L103. This reference should probably refer to Table S2, since Tables S3 and S4 do not show fire occurrences by mountain region.

Corrected.

L119. The percentage cover of each vegetation type below and above 1400 m would help the reader to better interpret the results.

We have now included a new supplementary table (Table S5a-b) reporting vegetation composition (%) by elevation class and fire regime type. The main text has been revised accordingly to incorporate the corresponding percentages and to better support the interpretation of vegetation patterns across elevations.

Revision: ***Table S5a. Distribution of burned area (ha) and median vegetation cover (%) across elevation classes (≤1,400 m and >1,400 m).***

Results L147-155

"Transitional vegetation was prevalent across elevations but dominated above 1,400 m, accounting for 55% of burned area in high and persistent regions and 50% in moderate and reoccurring regions, compared to 41–42% below 1,400 m. In contrast, broadleaf forests were generally more represented below 1,400 m in high and persistent (17%) and low and emerging regions (43%), although patterns varied among regime types (Table 1, S5a-b).

Mixed and conifer forests remained comparatively less dominant overall, despite some elevation-specific contrasts (e.g., higher conifer and mixed shares above 1,400 m in low and emerging regions). Human footprint was significantly lower above 1,400 m."

L123. Table 1. I suggest reporting p-values as $p < 0.001$ for clarity. The parameters that are not significantly different between below and above 1400 m should not be highlighted in bold (Tmin, human footprint). Moreover, units of climatic variables are missing and should be included.

Added. We thank the reviewer for the constructive comments. Units have now been added for all climatic variables, and fuel variables are explicitly reported as percentage cover. Significance is indicated using standard notation (* $p < 0.05$; ** $p < 0.01$; *** $p < 0.001$; ns = not significant), with exact p-values retained in the table for accuracy.

Revision: Table 1.

L135. It should be "fuel type" or "type of vegetation cover". Just "fuel" can be misleading, as one could interpret it as referring to fuel load.

Corrected everywhere in the manuscript.

L147. Figure 4. Lower panels should be (d) instead of (a). Soil moisture in moderate and reoccurring fire regimes is positively correlated with burned area, any idea why?

Corrected.

We thank the reviewer for raising this point. In the moderate and reoccurring regime (Fig. 3c), soil moisture shows a positive standardized coefficient ($\beta \approx 0.27$) with confidence intervals not overlapping zero, indicating a correlation with burned area. Its inclusive R^2 is approximately 0.03–0.04, highlighting a non-negligible contribution relative to other predictors in this regime. This pattern likely reflects a fuel-limitation mechanism, whereby higher antecedent soil moisture enhances vegetation productivity and fuel accumulation, which can subsequently promote larger burned areas under episodic drought or high VPD conditions.

***Discussion**

L149. "Fires climb higher" sounds a bit awkward. A clearer alternative could be "fires shift upslope" or a similar expression. Moreover, based on your analysis, how do you know that fires are intensifying? An increase in burned area does not necessarily imply higher fire intensity.

Corrected.

L153. Delete "after 2015", already mentioned before.

Corrected.

L158. Did you mean (800-1400 m) instead of (800-1600 m)?

Corrected.

L164. Socioeconomic drivers are represented by the human footprint parameter, which did not show any significant contribution driving fires upslope. I think that claiming that this study provides a framework that links fire activity to socioeconomic drivers seems overstated.

With the updated dataset (2000–2025), human footprint shows a statistically significant effect in the elevation-based analyses (Table 1), although its relative contribution remains smaller than key climatic variables such as VPD and soil moisture. We edited a bit the sentence.

L167. Careful with terminology ("severe fires"). Fire severity refers to fire effects on the ecosystem; fire intensity is the energy released by the fire. In this study burned area and fire occurrence are analysed, and they do not always relate to intensity or severity (Keeley, 2009).

L172. "Extreme fire weather" is more commonly used than "severe fire weather"

Thank you, both corrected.

L176. The analysis shows a minor role of vegetation composition in driving fires upslope. But the analysis did not include fuel load and structure as potential predictors of burned area. It should be acknowledged in the discussion.

Yes, indeed, we added that.

Revision: Discussion L240-242.

"We represent fuels via harmonized land-cover fractions, which do not resolve fuel loads or vertical structure that may further influence mountain fire spread."

L198. Increasing fuel load/continuity/cover?

Corrected: cover.

L214. Besides silvicultural treatments, prescribed burning can also be a valuable tool to reduce fuel load and continuity and increase resistance to wildfires (e.g., Vilà-Vilardell et al., 2023).

Added.

L215. Reduce fuel continuity AND LOAD.

Added.

L216. Instead of "coordinated fuel management", a term which I believe is not much used, you could refer to "integrated fire management" (e.g., Oliveras-Menor et al., 2025).

Added.

L225. The statement that fire activity has intensified is misleading, as this study did not analyse changes in fire intensity. To avoid misinterpretation, I suggest changing it to "fire activity has increased".

Added.

L231. Fine-scale monitoring of what?

Corrected. Forest monitoring.

*****Methods***

L260. Why did you exclude the small mountain ranges from the analysis?

Our focus is on big mountain ranges. Hence, small mountain ranges (e.g., Aegean Islands, Crete, Sardinia, Corsica) were excluded due to insufficient fire counts for robust regional trend analysis.

L287. The resolution of the climate data is quite coarse (4 km, downscaled to 1 km). This should be acknowledged as a limitation in the discussion, particularly because fine-scale climatic variability is common in mountain regions with complex topography (e.g., Schuck et al., 2011).

We mentioned that in the discussion and also followed your suggestion, hence to partly address this limitation, we resampled all climate layers to 1 km and applied a DEM-based lapse-rate correction to temperature (tmax, tmin), adjusting values according to local elevation anomalies using a standard environmental lapse rate (−6.5 °C km^{-1}). A full explanation is given above.

Revision:

Methods L352-353

"*Temperature was additionally adjusted using a DEM-based lapse rate correction (−6.5 °C km^{-1}) 79 to account for sub-grid elevational variability in mountainous terrain.*"

Discussion L271-272

"Similarly, high-resolution climate products, such as easyclimate 70, would further improve the detection of fine-scale climate–fire interactions in complex mountain terrain."

L288. The authors analyse fire occurrences and burned area using average yearly climate values. This approach is appropriate, but a deeper analysis of the seasonal distribution of fires would be valuable, as fire timing is likely to influence post-fire regeneration (e.g., Tangney et al., 2022). When do most fires occur in each mountain region, and has this timing shifted over the past two decades? Including a summary table would be interesting to show.

We thank the reviewer for this valuable suggestion. We have now conducted a detailed seasonal analysis of fire occurrence and added three supplementary tables (Fig. S2ab and Tables S4abc) summarizing peak and median fire months for each mountain region and comparing two periods (2006–2014 vs 2015–2025).

The analysis reveals pronounced regional differences and clear temporal shifts in several mountain systems. In the Balkans/Southeast Europe, Carpathians, and Iberian mountains, peak fire occurrence shifted from late summer (August) during 2006–2014 to early spring (March–April) during 2015–2025, corresponding to advances of 4–5 months. These shifts are highly significant (all $p < 10^{-8}$). The Alps also exhibit a significant advance in peak timing from July to March (−4 months; $p = 2.7 \times 10^{-3}$).

By contrast, no significant seasonal shift was detected in Turkey ($p = 0.76$), and the Pyrenees show only a minor, non-significant shift (−1 month; $p = 0.38$). The Apennines exhibit a statistically significant redistribution of monthly fire occurrence ($p = 2.9 \times 10^{-4}$), although the peak month remains August in both periods, indicating increased early-season activity rather than a shift in the dominant summer peak.

Together, these results demonstrate a substantial reorganization of fire seasonality in several European mountain regions over the past two decades. Earlier peak fire activity

in multiple regions suggests a lengthening or redistribution of the fire season, which may have important implications for post-fire regeneration dynamics and ecosystem resilience. The revised manuscript now incorporates this seasonal analysis and its ecological interpretation. These findings are consistent with increasing non-summer fires in Europe (D'Alò et al., 2026)**.**

Revision:

Results L117-123

"Fire seasonality differed among mountain regions and shifted over time (Fig. S2ab; Tables S4abc). For example, in the Balkans/Southeast Europe and Carpathians, peak fire occurrence shifted from late summer (August) before 2015 to early spring (March–April) after 2015 (Fig. S2ab), corresponding to advances of up to five months. In contrast, no significant seasonal shift was detected in Turkey or the Pyrenees (Table S4c). Across regions, elevation of fire occurrence increased significantly through the calendar year (β = 13.7 m per month, t = 8.98), although region-specific trends varied (Table S4d).

Discussion L197-199 "Further, we showed a shift toward spring fire activity in several mountain regions, consistent with increasing non-summer fires in Europe."

L289. The human footprint parameter is not clearly explained in the manuscript. Which variables were used to create this map? This information is important to interpret the results. Also, in Table 2 this variable is shown as unitless, but what is its range? More details should be provided, as this is not a commonly known parameter, at least within the wildfire community.

We thank the reviewer for pointing this out. Human pressure was represented using the Human Footprint Index (HFP), which is the only globally harmonized dataset providing consistent spatial coverage across all eight mountain regions over the full study period. The Human Footprint (HFP) dataset follows the global annual Human Footprint product of Mu et al. (2022) **which integrates multiple anthropogenic pressure variables, including built environments, population density, nighttime lights, cropland, pasture, roads, railways, and navigable waterways, into a composite index. The dataset is provided at 1 km spatial resolution and ranges from 0 (no detectable human pressure) to 50 (maximum cumulative human pressure), with values <10 representing low human influence, 10–30 moderate disturbance (e.g., scattered agriculture), and >30 high-impact zones such as urbanized regions. We have expanded the Methods section to briefly describe the components and range of the HFP index.**

Revision: Methods L353-360

"Human footprint integrates multiple anthropogenic pressure variables, including built environments, population density, nighttime lights, cropland, pasture, roads, railways, and navigable waterways, into a composite index ranging from 0 (no detectable human pressure) to 50 (maximum cumulative human pressure), with values <10 representing low human influence, 10–30 moderate disturbance (e.g., scattered agriculture), and >30 high-impact zones such as urbanized regions. Annual maps from 2000 to 2018 at 1 km spatial resolution were obtained from Mu et al. (2022)***."***

L307. The significant intensification of fire activity is based on which data source? Is it derived from your dataset? This should be clearly stated.

The statement refers to the intensification observed in our compiled EFFIS-derived fire dataset (2000–2025), which shows a marked increase in fire occurrence and burned area after 2015 across multiple mountain regions. We have revised the text to explicitly state that the identified intensification is based on trends derived from our dataset.

Revision: Methods L375-379

"To test for differences in the distribution of fire elevations between the early (2000–2014) and late (2015–2025) study periods, we applied two-sample Kolmogorov–Smirnov tests. The latter period was chosen because it corresponds to a marked increase in fire occurrence and burned area in Europe."

L308. How were fires classified within the elevation classes? Based on the mean elevation of each fire or the maximum? This should be specified, especially since the 800 m threshold to include wildfire in the analysis was defined using the maximum elevation.

Fires were classified into 200 m elevation classes based on the maximum elevation (Z_max) of each fire polygon. The 800 m inclusion threshold was also defined using Z_max ≥ 800 m to ensure consistency. We have now clarified this explicitly in the Methods section.

Revision: Methods L379-380

"Temporal trends in fire occurrence across elevation classes (800–3,000 m, in 200 m intervals, based on maximum elevation per polygon) were modeled using generalized linear mixed models (GLMMs) with year as a fixed effect and elevation class as a random effect".

L309. From Table S3, I see that Year was included as a numeric predictor. This should be clearly explained and justified in the methods section. Using Year as a numeric predictor implies a linear relationship between fire occurrences and time, which may not always be the case. Thus, the choice to include Year as numeric should either be properly justified or Year should be included as a categorical factor. Also, Year was included as a fixed effect, but also as a random slope within Elevation class. This should also be explained and justified. Additionally, which GLMM family did you use? And why was "mountain range" not included as a random effect? How did you account for the dependency of the data related to location?

Year as a numeric predictor implies a linear relationship. Fig. 2c shows a smoothed regression over the entire studied period (2000-2025). However, statistical analyses were applied separately to two periods; 2000-2014 during which no clear trend was observed (values are reported as mean ± standard deviation for each elevation class; Table S3d), and 2014–2025, during which we identified an increasing, elevation-dependent trend in fire occurrence. Linear regression provided the best model fit.

Random slopes. Year was included both as a fixed effect and as a random slope within elevation class (Year | Elevation class) to allow temporal trends to vary across elevation

classes. This structure relaxes the assumption of a uniform temporal response and captures differential rates of change along the elevational gradient.

Model family and transformation. Fire occurrence was log-transformed prior to analysis because the distribution was strongly right-skewed, with a small number of years exhibiting very high fire counts. Diagnostic checks indicated that the log transformation substantially improved residual structure and homoscedasticity. The model was therefore fitted using a Gaussian error distribution. We have clarified this rationale in the table caption from the supplementary S3c.

Mountain range as random effect and spatial dependency. We used elevation class as the random grouping factor to focus on elevation-specific temporal trends. The trend was assessed at the European scale, independently of individual mountain regions. Several mountain regions had very low or zero annual fire occurrence at a given year x elevation-class, meaning that including mountain range as a random intercept would have reduced model stability and failed to ensure sufficient replication within groups. Because the response variable was aggregated at the annual × elevation-class level, spatial dependence at the individual fire level was reduced. We now clarify this modeling choice and its implications in the revised manuscript.

Revision:

Table S3c. Summary of generalized linear mixed models of the temporal trends in fire occurrence with elevation classes as a random effect. Table S3d. Temporal trends of forest fire occurrence before and after 2015.

L320. For the GAM, same as with the previous model, what is the rationale for including Year as a numeric variable? Why did you choose GAM and not GAMM with a random intercept that accounts for dependency, instead? Moreover, where are the numerical results of this model?

Year as numeric predictor. Year was included as a continuous variable to capture smooth temporal trends in fire occurrence and burned area over time. In the GAM framework, Year was modeled using a penalized spline, allowing for non-linear temporal responses rather than imposing a strictly linear trend.

Why GAM instead of GAMM. The GAM was fitted to annual aggregated data at the continental scale, where observations represent year-level summaries rather than repeated measurements from spatially nested units. Because the response variable did not retain a hierarchical or spatial grouping structure at this stage, a GAMM with random intercepts was not required.

The GAM model is shown on Fig. 2c. However, statistical analyses were applied separately to two periods; 2000-2014 during which no clear trend was observed (values are reported as mean ± standard deviation for each elevation class; Table S3d), and 2014–2025, during which we identified an increasing, elevation-dependent trend in fire occurrence. Linear regression provided the best model fit.

L334. The rationale to choose 1400 m as a threshold should be more clearly justified.

The 1,400 m threshold was informed by the elevation breakpoint analysis which we have included now in the Table S7. Across all variables, the average European cutpoint was 1,458 m and the median 1,568 m, with regime-specific medians ranging from 1,069 m (high and persistent) to 1,618 m (moderate and reoccurring). In low and emerging regimes, the median breakpoint was 1,380 m. Thus, 1,400 m represents a central tendency across multiple significant climatic and vegetation thresholds and falls within one standard deviation of the overall breakpoint distribution. We have clarified in the Methods that this threshold reflects the approximate elevation at which multiple fire–environment relationships shift, rather than an arbitrary division and we added additional tables in the supplementary with distribution of fires along with the vegetation above and below 1,400 m across mountain regions (Table S5ab).

Revision:

Table S5ab Vegetation composition across fire region types in European mountains, Table S7 Elevational breakpoints (cutpoints, m) derived from statistical change-point analyses.

Methods L405-412.

"The 1,400 m threshold reflects the central tendency of statistically derived elevation breakpoints across climatic, fuel type, and human variables (Table S7), which cluster around 1,400–1,600 m at continental scale. Vegetation composition within burned areas was quantified as the percentage cover of broadleaf, conifer, mixed, and transitional vegetation intersecting each fire polygon. Burned area and median vegetation cover were summarized by elevation class (≤1,400 m and >1,400 m) and fire regime type (Table S5a), and by mountain region and elevation class (Table S5b). Burned area distributions across regime types were examined using log-transformed burned area and kernel density estimates (Fig. S3)."

L339. Parenthesis missing after transitional.

Corrected.

L350. Why burned area was log-transformed? Did you test with other GLMM families rather than Gaussian? Log-scaling is not optimal, since it changes the relationship between the response and predictor variables (Zuur et al., 2009, 2010). And again, why not including mountain range as random intercept?

We thank the reviewer for raising this important point. Following the data exploration protocol recommended by Zuur et al. (2009, 2010), we first inspected the distribution of burned area and model residuals. Burned area showed a strongly right-skewed distribution with a small number of very large fires. Preliminary models using the untransformed response resulted in substantial heteroscedasticity and non-normal residuals. Log-transformation substantially improved residual structure and variance homogeneity. Because burned area is a continuous variable, binomial models were not appropriate. We explored alternative specifications and retained the log-transformed Gaussian GLMM because it provided the most stable residual diagnostics and interpretable model fit.

Regarding spatial structure, elevation class was included as the random grouping factor because the objective of the model was to quantify elevation-specific temporal trends across Europe. Including mountain range as an additional random intercept would have reduced model stability due to very uneven replication across regions and years, with several elevation–region combinations containing few or no observations. Moreover, because the response variable was aggregated at the annual × elevation-class level, spatial dependence among individual fires was already reduced.

L357. The term "region type" is confusing, as it suggests mountain regions. Instead, I recommend replacing it by "fire regime type".

Corrected, thanks.

****Supplementary material***

Table S1. For clarity, I suggest changing the column order of the mountain ranges to match the order used for the fire regime types (red-green-yellow; red-green-yellow).

Corrected. All the supplementary materials were updated, we included additional tables summarizing the data used, e.g. Tables S1ab.

Figure S2. Labels of figures are missing (a to f).

Corrected.

Table S4. Where do these results come from? It is not clear.

The slopes reported in Table S4 are derived from linear regressions fitted separately for each elevation class and time period (2001–2014 and 2015–2024), using annual fire counts as the response variable and Year as a continuous predictor. Slopes therefore represent the annual change in fire occurrence (fires per year) within each elevation band. Before 2015, trends were generally non-significant, and thus slopes were not emphasized in the main text.

****References***

Conedera, M., Krebs, P., Valese, E., Cocca, G., Schunk, C., Menzel, A., ... & Pezzatti, G. B. (2018). Characterizing Alpine pyrogeography from fire statistics. Applied Geography, 98, 87-99.

Keeley, J. E. (2009). Fire intensity, fire severity and burn severity: a brief review and suggested usage. International journal of wildland fire, 18(1), 116-126.

Kreider, M.R., Higuera, P.E., Parks, S.A. et al. Fire suppression makes wildfires more severe and accentuates impacts of climate change and fuel accumulation. Nat Commun 15, 2412 (2024). https://doi.org/10.1038/s41467-024-46702-0

Linley, G. D., Jolly, C. J., Doherty, T. S., Geary, W. L., Armenteras, D., Belcher, C. M., ... & Nimmo, D. G. (2022). What do you mean,'megafire'?. Global Ecology and Biogeography, 31(10), 1906-1922.

Oliveras Menor, I., Prat-Guitart, N., Spadoni, G. L., Hsu, A., Fernandes, P. M., Puig-Gironès, R., ... & Armenteras Pascual, D. (2025). Integrated fire management as an adaptation and mitigation strategy to altered fire regimes. Communications Earth & Environment, 6(1), 202.

Schunk, C., Wastl, C., Leuchner, M., Schuster, C., & Menzel, A. (2013). Forest fire danger rating in complex topography–results from a case study in the Bavarian Alps in autumn 2011. Natural hazards and earth system sciences, 13(9), 2157-2167.

Stoof, C. R., De Vries, J. R., Castellnou Ribau, M., F. Fernández, M., Flores, D., Galarza Villamar, J., ... & Fernandes, P. M. (2024). Megafire: An ambiguous and emotive term best avoided by science. Global Ecology and Biogeography, 33(2), 341-351.

Tangney, R., Paroissien, R., Le Breton, T.D. et al. Success of post-fire plant recovery strategies varies with shifting fire seasonality. Commun Earth Environ 3, 126 (2022). https://doi.org/10.1038/s43247-022-00453-2

Tedim, F., Leone, V., Amraoui, M., Bouillon, C., Coughlan, M. R., Delogu, G. M., ... & Xanthopoulos, G. (2018). Defining extreme wildfire events: Difficulties, challenges, and impacts. Fire, 1(1), 9.

Turco, M., Jerez, S., Augusto, S. et al. Climate drivers of the 2017 devastating fires in Portugal. Sci Rep 9, 13886 (2019). https://doi.org/10.1038/s41598-019-50281-2

Vilà-Vilardell, L., De Cáceres, M., Piqué, M., & Casals, P. (2023). Prescribed fire after thinning increased resistance of sub-Mediterranean pine forests to drought events and wildfires. Forest Ecology and Management, 527, 120602.

Zuur, A. F., Ieno, E. N., Walker, N. J., Saveliev, A. A., & Smith, G. M. (2009). Mixed effects models and extensions in ecology with R (Vol. 574, p. 574). New York: Springer.

Zuur, A. F., Ieno, E. N., & Elphick, C. S. (2010). A protocol for data exploration to avoid common statistical problems. Methods in ecology and evolution, 1(1), 3-14.

Thank you for providing these references, they were very useful in understanding the suggestions made and in applying those changes.

****(Remarks on code availability):***

The rgdal package has not been available since 2023 (https://r-spatial.org/r/2022/04/12/evolution.html)

The R code provided should be revised. I got several errors.

We updated the scripts and uploaded them to Zenodo.

Thank you for helping us to improve the manuscript!

2 References

Conedera, M., Krebs, P., Valese, E., Cocca, G., Schunk, C., Menzel, A., et al. (2018) Characterizing Alpine pyrogeography from fire statistics. *Applied Geography*, 98, 87–99. https://doi.or/10.1016/j.apgeog.2018.07.011

D'Alò, D., Elia, M., Fernandez, C.N., Argañaraz, J.P., Amoroso, N., Giannico, V., et al. (2026) Beyond summer fires: shifting forest fire across European biogeographical regions. *Environmental Research Letters*, 21(5), 054007. https://doi.or/10.1088/1748-9326/ae4817

Ellis, T.M., Bowman, D.M.J.S., Jain, P., Flannigan, M.D., & Williamson, G.J. (2022) Global increase in wildfire risk due to climate-driven declines in fuel moisture. *Global Change Biology*, 28(4). https://doi.or/10.1111/gcb.16006

Kapos, V., Rhind, J., Edwards, M., Price, M.F., & Ravilious, C. (2000) Developing a map of the world's mountain forests. *Forests in sustainable mountain development: a state of knowledge report for 2000. Task Force on Forests in Sustainable Mountain Development.*

Kreider, M.R., Higuera, P.E., Parks, S.A., Rice, W.L., White, N., & Larson, A.J. (2024) Fire suppression makes wildfires more severe and accentuates impacts of climate change and fuel accumulation. *Nature Communications*, 15(1), 2412. https://doi.or/10.1038/s41467-024-46702-0

Mary Petritan, A., & Beloiu Schwenke, M. (2023) Forest Functioning under Climate Warming and Future Perspectives on Forest Disturbances. *Forests*, 14(12), 2302. https://doi.or/10.3390/f14122302

Mu, H., Li, X., Wen, Y., Huang, J., Du, P., Su, W., et al. (2022) A global record of annual terrestrial Human Footprint dataset from 2000 to 2018. *Scientific Data*, 9(1). https://doi.or/10.1038/s41597-022-01284-8

San-Miguel-Ayanz, J., Durrant, T., Boca, R., Libertà, G., Branco, A., Costa, H., et al. (2018) *Forest fires in Europe, Middle East and North Africa 2017*.

San-Miguel-Ayanz, Jesús., Durrant, Tracy., Boca, Roberto., Maianti, Pieralberto., Libertá, Giorgio., Artés-Vivancos, T.Artés., et al. (2022) *Advance report on wildfires in Europe, Middle East and North Africa 2021.* Publications Office of the European Union: Luxembourg.

San-Miguel-Ayanz, Jesús., Durrant, Tracy., Boca, Roberto., Maianti, Pieralberto., Libertá, Giorgio., Oom, Duarte., et al. (2023) Advanced report on Forest Fires in Europe, Middle East and North Africa 2022. *Publications Office of the European Union*, (EUR 31028 EN).

Turco, M., Bedia, J., Di Liberto, F., Fiorucci, P., Von Hardenberg, J., Koutsias, N., et al. (2016) Decreasing fires in mediterranean Europe. *PLoS ONE*, 11(3). https://doi.or/10.1371/journal.pone.0150663

Turco, M., Jerez, S., Augusto, S., Tarín-Carrasco, P., Ratola, N., Jiménez-Guerrero, P., & Trigo, R.M. (2019) Climate drivers of the 2017 devastating fires in Portugal. *Scientific Reports*, 9(1), 13886. https://doi.or/10.1038/s41598-019-50281-2

Response to Reviewer 1

We thank Reviewer 1 for the constructive and thoughtful evaluation of our manuscript. In response, we improved methodological clarity and expanded key analyses. Specifically, we:

1. Clarified the treatment of climatic variables and added a correlation analysis (Fig. S4e);
2. Extended elevational analyses by including Z_mean (mean fire elevation) alongside Z_max and added Fig. S1b;
3. Refined the results and shortened the abstract to meet journal guidelines.

All analyses and figures were updated accordingly.

A detailed point-by-point response to all reviewer comments is provided below. Reviewer comments are shown in black, our responses in blue, and changes in the revised manuscript are indicated in red.

Thank you for helping us to improve the manuscript.

Yours sincerely

Mirela Beloiu

On behalf of the authors

1 # Reviewer 1

The authors have done an excellent work addressing the concerns raised in the previous round. The revisions substantially improve the manuscript, and the responses are thorough and convincing. In particular, I commend the careful re-evaluation of the EFFIS database, detecting the forest cover estimation inconsistences, This significantly strengthens the robustness and reliability of the analysis, and also confirms that this peak is not due to an artifact caused by the sensor change. The additional methodological clarifications, the sensitivity analysis of the Canadian Fire Weather Index, and the expanded discussion of human influence are all well integrated and enhance the overall quality of the work.

One remaining point deserves further consideration. While the implementation of a DEM-based first-order lapse-rate correction for temperature is appropriate and improves the representation of thermal gradients, limiting the downscaling procedure to temperature alone may introduce inconsistencies in mountainous environments. Other key climatic variables relevant to fire dynamics —such as precipitation, VPD, PET — also exhibit strong spatial heterogeneity driven by microtopography. In particular, precipitation gradients can critically influence fuel moisture and, consequently, fire occurrence and behavior. Moreover, the use of mixed spatial resolutions across predictors (i.e., downscaled temperature versus coarser-resolution moisture-related variables) may introduce internal inconsistencies in the analysis, potentially affecting model sensitivity and the relative importance attributed to different drivers. In this context, it would be preferable to apply a consistent downscaling strategy across all key climatic variables, or alternatively to retain all variables at their native resolution. A brief discussion acknowledging these limitations and justifying the adopted approach would further strengthen the methodological transparency of the study.

Finally, I find the composite quinquennial centroids figure to be excellent, as it greatly enhances the clarity of the spatiotemporal patterns described in the manuscript.

We thank the reviewer for this important comment. All climatic variables were first resampled from their native ~4 km resolution to 1 km using bilinear interpolation to ensure a consistent spatial grid across predictors. In addition, temperature (tmin and tmax) was further adjusted using a DEM-based lapse-rate correction to better represent sub-grid elevational variability in mountainous terrain (as was suggested by the reviewer in the first round of revisions). We limited the lapse-rate correction to temperature because its relationship with elevation is well defined and approximately linear. In contrast, other key variables such as precipitation, VPD, and PET are influenced by complex and non-linear orographic processes (e.g., wind exposure, moisture transport, and rain-shadow effects), and cannot be reliably downscaled using a simple DEM-based correction (Karger et al., 2017)**. Applying independent downscaling to these variables could introduce additional uncertainty and disrupt the internal consistency of the TerraClimate variables.**

Importantly, temperature was not retained in the final models (*Fig. 4. Predictors of burned area in European mountain regions and within fire regime types*), as it showed strong

collinearity with atmospheric dryness variables (e.g., VPD, DEF, PET), which were identified as the dominant drivers of burned area (Fig. S4e). This further limits any potential inconsistency arising from differential downscaling. We have now included a correlation matrix (Fig. S4e) to explicitly document these relationships. Temperature (Tmin, Tmax) was included in Table 1 (*Elevation-based differences in median values of climatic, fuel, and human footprint variables across fire occurrences (D800) above and below 1,400 m*) and Table S7 as an independent variable and analyzed individually, rather than jointly with other predictors, to describe elevation-based differences in thermal conditions associated with fire occurrence.

Finally, we note that our analysis focuses on continental-scale patterns across European mountain regions. While finer-scale topo-climatic variability may influence local fire dynamics, applying more complex downscaling approaches at this scale could introduce additional bias and reduce the internal consistency of the dataset. We now explicitly acknowledge this limitation and highlight the potential value of high-resolution climate products for future regional analyses.

Changes: S4e Correlation matrix included in the supplementary (see below).

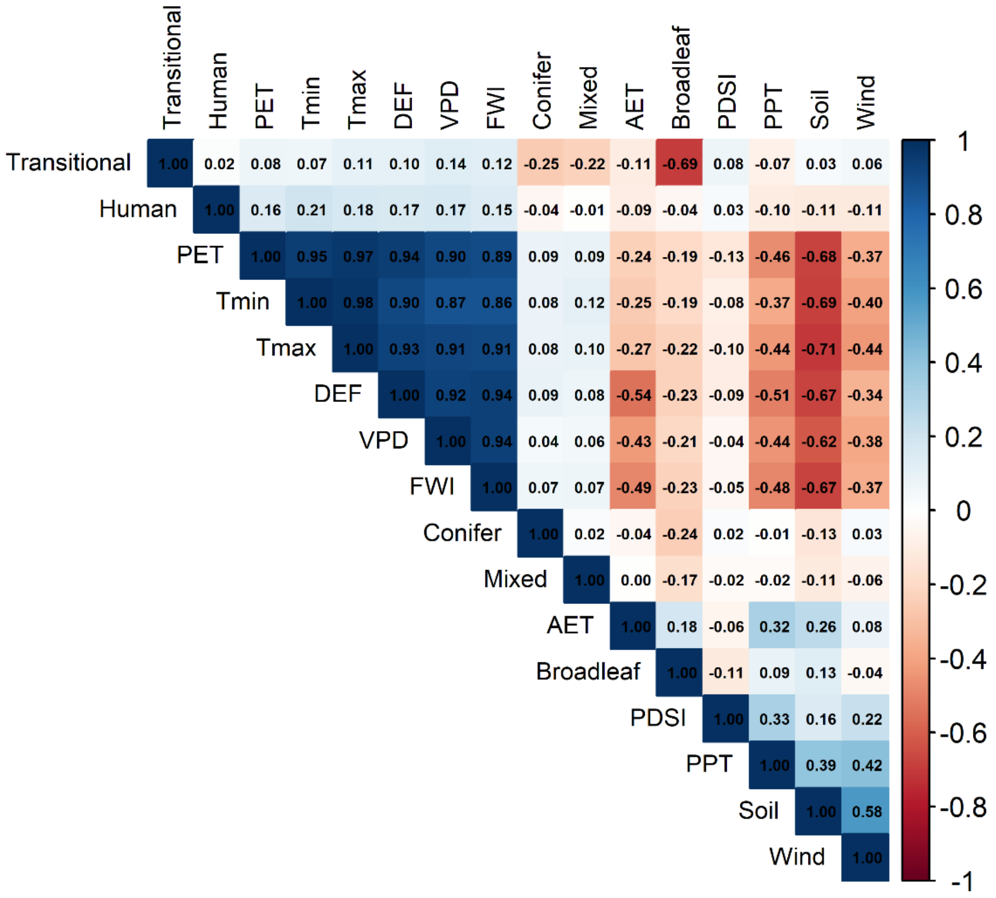

Figure S4d. Correlation matrix of climatic, fuel, and human variables across fire events. Strong positive correlations are observed among atmospheric demand variables (VPD, DEF, PET, Tmax), while soil moisture and precipitation are negatively correlated with these variables. The Fire Weather Index (FWI) shows strong positive correlations with VPD and DEF and negative correlations with soil moisture and precipitation, indicating that it integrates key aspects of climatic fire risk.

In addition, we expanded the analysis to better highlight the elevational shift in fire activity over time. We added Fig. S1b, showing the upslope shift in fire elevation before and after 2015 based on both mean (Z_mean) and maximum (Z_max) fire elevation. We also extended *Table S3a. Upslope shift in European mountain fire elevation before and after 2015* and *Table S3b. Linear regression slopes of yearly elevational fire metrics (2000–2025)* by including results based on Z_mean in addition to the previously reported Z_max metrics. This allows a more comprehensive assessment of elevational changes, capturing both average fire elevation and extremes.

Furthermore, based on *Table S3d. Temporal trends of forest fire occurrence before and after 2015,* we now explicitly report changes in fire frequency below and above 1,400 m in the Results section and Abstract. For example, before 2015, mean annual fire occurrence averaged 32.8 fires yr^{-1} below 1,400 m and 8.2 fires yr^{-1} above, indicating substantially higher fire activity at lower elevations. After 2015, fire occurrence increased across all elevation classes, but more strongly below 1,400 m (+9.5 fires yr^{-1}; ~29% yr^{-1}) than above (+1.0 fires yr^{-1}; ~12% yr^{-1}).

We thank the reviewer for the positive feedback on the composite quinquennial centroids figure. We further enhanced Fig. 1 by adding a subtle grey shading of mountain areas to improve the visual interpretation of fire distributions relative to topography.

References

Karger, D.N., Conrad, O., Böhner, J., Kawohl, T., Kreft, H., Soria-Auza, R.W., et al. (2017) Climatologies at high resolution for the earth's land surface areas. *Scientific Data*, 4, 170122. https://doi.or/10.1038/sdata.2017.122

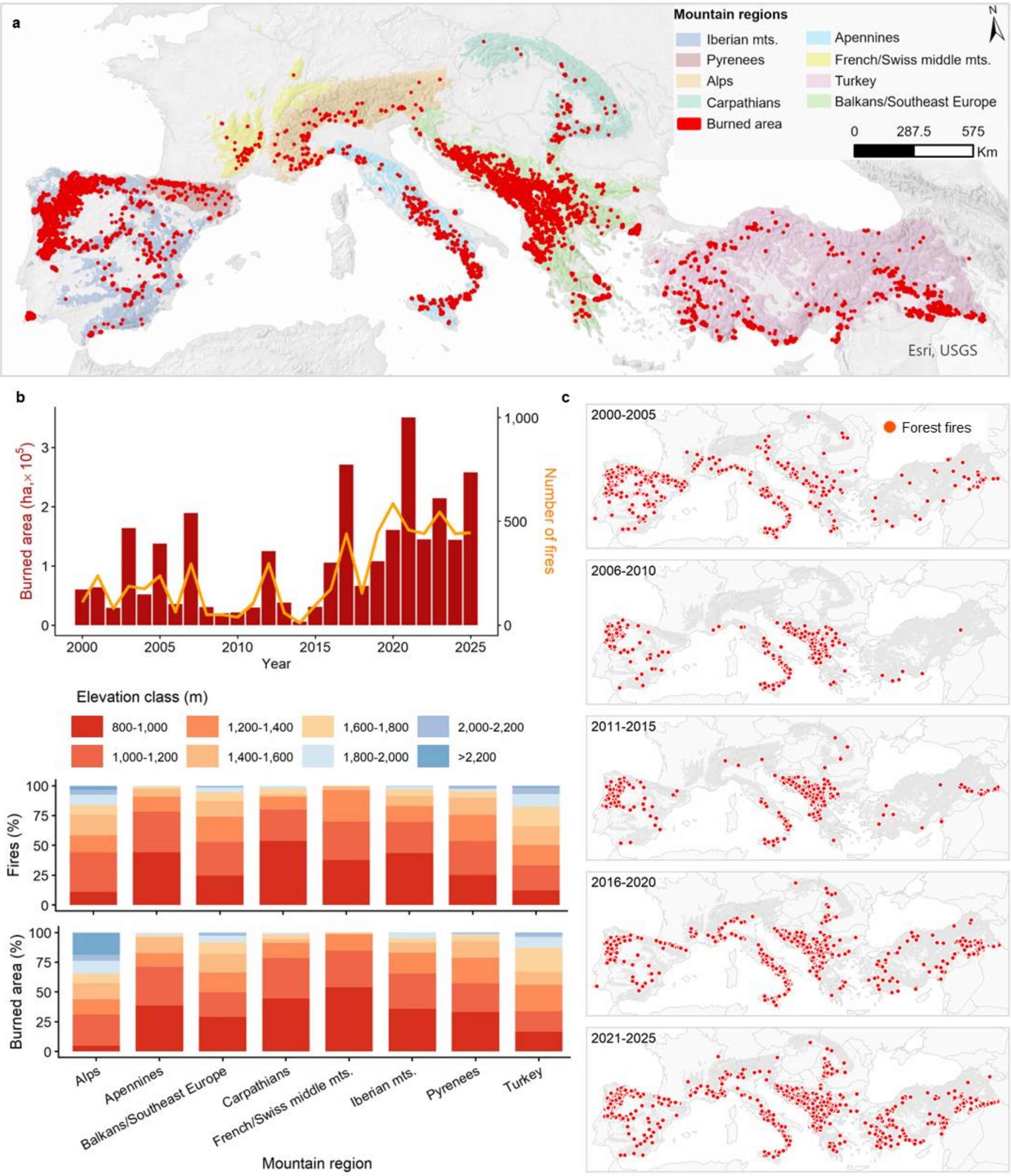

Fig. 1. a Spatial distribution of forest fires (no = 6,225) that occurred above 800 m elevation within the eight European mountain regions from 2000 to 2025. b *Evolution of burned area and number of fires within mountain regions of Europe and percentage of fire occurrences and burned area (ha) for each mountain region and elevation class.* ***c*** *Spatial distribution of forest fires across European mountain regions in five time periods (2000–2005, 2006–2010, 2011–2015, 2016–2020, and 2021–2025), illustrating the expansion and clustering of fire occurrences over time. Annual fire distributions are shown in Fig. S1. The extent of mountain regions was defined following Kapos et al.* [37].
